# Testing a computational model of causative overgeneralizations: Child judgment and production data from English, Hebrew, Hindi, Japanese and K’iche’

**DOI:** 10.12688/openreseurope.13008.1

**Published:** 2021-03-24

**Authors:** Ben Ambridge, Laura Doherty, Ramya Maitreyee, Tomoko Tatsumi, Shira Zicherman, Pedro Mateo Pedro, Ayuno Kawakami, Amy Bidgood, Clifton Pye, Bhuvana Narasimhan, Inbal Arnon, Dani Bekman, Amir Efrati, Sindy Fabiola Can Pixabaj, Mario Marroquín Pelíz, Margarita Julajuj Mendoza, Soumitra Samanta, Seth Campbell, Stewart McCauley, Ruth Berman, Dipti Misra Sharma, Rukmini Bhaya Nair, Kumiko Fukumura

**Affiliations:** 1 University of Liverpool, Liverpool, UK; 2 ESRC International Centre for Language and Communicative Development (LuCiD), Liverpool, UK; 3 Kobe University, Kobe, Japan; 4 Hebrew University of Jerusalem, Jerusalem, Israel; 5 Universidad del Valle de Guatemala, Guatemala City, Guatemala; 6 University of Salford, Salford, UK; 7 University of Kansas, Lawrence, Kansas, USA; 8 University of Colorado, Boulder, Boulder, Colorado, USA; 9 University of Calgary, Calgary, Canada; 10 University of Iowa, Iowa City, Iowa, USA; 11 Tel Aviv University, Tel Aviv, Israel; 12 Indian Institute of Information Technology, Hyderabad, India; 13 Indian Institute of Technology, Delhi, India; 14 University of Stirling, Stirling, UK

**Keywords:** child language acquisition, verb semantics, causative, English, Japanese, Hindi, Hebrew, K’iche', discriminative learning

## Abstract

How do language learners avoid the production of verb argument structure overgeneralization errors ( *The clown laughed the man c.f. *The clown made the man laugh*), while retaining the ability to apply such generalizations productively when appropriate? This question has long been seen as one that is both particularly central to acquisition research and particularly challenging. Focussing on causative overgeneralization errors of this type, a previous study reported a computational model that learns, on the basis of corpus data and human-derived verb-semantic-feature ratings, to predict adults’ by-verb preferences for less- versus more-transparent causative forms (e.g., * *The clown laughed the man *vs *The clown made the man laugh*) across English, Hebrew, Hindi, Japanese and K’iche Mayan. Here, we tested the ability of this model to explain binary grammaticality judgment data from children aged 4;0-5;0, and elicited-production data from children aged 4;0-5;0 and 5;6-6;6 ( *N*=48 per language). In general, the model successfully simulated both children’s judgment and production data, with correlations of *r*=0.5-0.6 and *r*=0.75-0.85, respectively, and also generalized to unseen verbs. Importantly, learners of all five languages showed some evidence of making the types of overgeneralization errors – in both judgments and production – previously observed in naturalistic studies of English (e.g., *I’m dancing it). Together with previous findings, the present study demonstrates that a simple discriminative learning model can explain (a) adults’ continuous judgment data, (b) children’s binary judgment data and (c) children’s production data (with no training of these datasets), and therefore constitutes a plausible mechanistic account of the retreat from overgeneralization.

## Plain language summary

When learning their native language, children often produce errors in which they use verbs in "ungrammatical" sentence types (e.g., “The clown laughed the man”, whereas an adult would say “The clown made the man laugh”). Although these examples are from English, similar errors are observed in many other languages including Hebrew, Hindi, Japanese and K’iche Mayan. A previous study reported a computer model which, when trained on an approximation of real language input, simulated the relative grammatical acceptability of these errors with different verbs as judged by child and adult raters. The aim of this study was to investigate whether the same model could explain (a) binary judgments from younger children (4–5 year-olds, who were simply asked "Is this sentence acceptable" rather than "How acceptable is this sentence?" and (b) the rates at which children learning these five languages actually produce such errors for different verbs (e.g., Someone laughed/danced/sang the boy). In general, the model performed very well on both tasks for all five languages.

## Introduction

The question of how language learners come to avoid verb argument structure overgeneralization errors such as *The clown laughed the man – in some cases after a protracted period of producing them – has been described as a “learnability paradox” ( Pinker, 1989: 415); “one of the most…difficult challenges for all students of language acquisition” ( Bowerman, 1988: 73). The problem is this: On the one hand, children need to be able to use verbs in argument structure constructions in which they have not witnessed them; this type of productivity is the hallmark of human language. On the other hand, children need to be able to constrain this generalization process in order to avoid producing ungrammatical utterances such as *The clown laughed the man. These types of errors, in which English-speaking children incorrectly mark causation using the transitive causative for verbs that prefer the periphrastic causative (e.g., *The clown made the man laugh*) are the focus of the present study; along with equivalent errors in Hebrew, Hindi, Japanese and K’iche Mayan. Further naturalistically obtained examples of this error are summarized in Table 1 below (from the diary study of Ambridge & Ambridge, 2020). Similar errors have been observed in naturalistic data for Japanese ( Nakaishi, 2016; see also the experimental study of Fukuda & Fukuda, 2001), though they have not, to our knowledge, been investigated for any of the other languages included here.

**Table 1.** Transitive causative overgeneralization errors produced by an English-speaking child (reproduced under a CC BY 4.0 license from Ambridge, 2019; also reproduced in Ambridge & Ambridge, 2020).

Age	Error
2;3	Can you reach me? (Already being held, wants lifting up higher to touch sparkly part of a sign)
2;4	Can you jump me off? (wants help jumping down off the bed)
2;4	Did you drop the letters? (="Did you make the letters drop?” Foam letters stuck to the bathroom wall have fallen into the bath)
2;6	(Dad: why are you running?) It's practising me to run like that
2;6	jump me!
2;6	Don't swim me
2;7	Run me down, jump me down (wants to run down slide)
2;7	Jump me
2;7	Drink me. drink me, Dad! (Can't reach juice in bottom of cup and wants it tipped right back)
2;7	I'm just dancing it (shaking the bent-double flap of the elephant's door in Dear Zoo, to make it dance)
2;7	I can dance it (book)
2;7	I'm dancing it
2;7	This is the boat - swim it!
2;7	Swim that aeroplane (submarine)
2;7	Stay your leg up there (holding dad's leg)
2;7	Stop jumping them (Dad is tapping rabbits in Peter Rabbit game to make them jump)
2;7	drink me a bit (wants straw held up to her mouth to drink squash in bed)
2;10	The sheet's slipping me
2;11	Jump me, Dad! x5
2;11	I jumped my legs. I hopped my legs
3;2	I stand on your feet and you walk me
3;2	(Mum: what happens to the rubbish when it goes outside?). It gets died.
3;5	(Dad, playing with Shopkins: Now what are we doing?) Chloe: Going them in. (What?) Into the bathroom
3;6	I'm try to duck her under (pushing Aurora doll under the seat belt of Barbie car)
3;6	Pens are difficult to come off the paper
3;7	Reach me up there (wants to see toys on top shelf)
3;7	It will get died [die/get killed]
3;7	That nearly feeled me like I'm nearly falling off
3;8	I'm going it faster (exercise bike at airport)
3;8	Eat it in my mouth (pez sweet that has fallen onto floor - wants Dad to pick it up and post it into her mouth)
3;8	Disappear them and disappear them (scooping up bubbles in the bath)
3;9	Your turn to dance me, Dad (i.e., swing her around by the arms)
3;10	Those guys died Maleficent (watching Sleeping Beauty)
3;10	We died (dissolved) Mummy's special soap didn't we, Dad?
3;11	Jump me up there (wants putting onto the toilet seat)
3;11	I wanna jump her in (Ariel doll into bath)
3;11	It will die you; it will make you killed
4;0	Mermaids have got special powers; they can die baddies
4;7	Jump me x 2

 This problem has attracted a great deal of research attention ( Alishahi & Stevenson, 2008; Ambridge *et al.*, 2008; Ambridge *et al.*, 2009; Brooks & Zizak, 2002; Brooks *et al.*, 1999; Gropen *et al.*, 1991; Li & MacWhinney, 1996; Perfors *et al.*, 2010; Stefanowitsch, 2008; Theakston, 2004; Wonnacott *et al.*, 2008); Ambridge, 2013; Ambridge & Ambridge, 2020; Ambridge & Blything, 2016; Ambridge & Brandt, 2013; Ambridge *et al.*, 2011; Ambridge *et al.*, 2012; Ambridge *et al.*, 2012; Ambridge *et al.*, 2013; Ambridge *et al.*, 2014; Ambridge *et al.*, 2015; Ambridge *et al.*, 2018; Barak *et al.*, 2016; Bidgood *et al.*, 2014; Boyd & Goldberg, 2011; Blything *et al.*, 2014; Goldberg, 2011; Harmon & Kapatsinski, 2017; Hsu & Chater, 2010; Irani, 2009; Perek & Goldberg, 2017; Robenalt & Goldberg, 2015; Robenalt & Goldberg, 2016; Twomey *et al.*, 2014; Twomey *et al.*, 2016), including two book-length treatments ( Goldberg, 2019; Pinker, 1989). However, until a single recent study, research on the retreat from overgeneralization had been conducted exclusively on English (and mainly on dative and locative constructions).

 This recent study ( Ambridge *et al.*, 2020), sought to explain how speakers learn to avoid not only causative errors in English, (e.g., *The clown laughed the man), but also equivalent errors in Hebrew, Hindi, Japanese and K’iche’ Mayan. It also adopted a novel theoretical approach: Previous studies had attempted to explain this phenomenon in terms of three – to some extent – competing theories: preemption, conservatism via entrenchment (both statistical-learning theories) and verb semantics. Ambridge *et al.* (2020) sought to unify these theories by building a computational model that yields all three effects in a single learning mechanism.

The model developed by Ambridge *et al.* (2020) – a simple two-layer connectionist network – is trained on input-output pairs consisting of a verb (e.g., *break*) and a causative type (e.g., for English, either the transitive causative or the *make* periphrastic causative respectively), in proportion to the frequency of each in a representative input corpus (e.g., for English, the frequency of *[CAUSER] [BREAK] [CAUSEE] vs [CAUSER] [MAKE] [CAUSEE] BREAK*). Other corpus utterances containing the relevant verb (e.g., intransitive *[ACTOR] [BREAK]*) are mapped to a catch-all “Other” output node. Crucially, the input to the model consists not only of an orthogonal (one-hot) “lexical” verb representation that uniquely identifies each verb stem, but also four “semantics” units. The (continuous) activation level of these units is set on the basis of human ratings of four semantic properties thought to be relevant to languages’ preferences for less-transparent (e.g., *X broke Y*) versus more-transparent ( *X made Y break*) causative forms respectively (e.g., Shibatani & Pardeshi, 2002) ^1^. These semantic ratings were obtained by showing native adult speakers of each language an animation depicting the action described by each verb (though they were not given the verb itself) and asking them to rate:

**Event-merge:** The extent to which the causing and caused event are two separate events or merge into a single event that happens at a single time and a single point in space

**Autonomy** of the causee

**Requires:** Whether the caused event requires a causer

**Directive:** Whether causation is directive (e.g., giving an order) or physical

It is important to note that the model was not given any information regarding human judgments of the grammatical acceptability of the more- and less-transparent causative forms of each verb (which would make its learning task trivially simple, and akin to a conventional statistically regression model conducted on participants’ grammaticality judgments). At test, the model was presented with each verb ( *N*=60) and interrogated for its prediction of a causative form (e.g., for English, transitive causative *vs* periphrastic causative with *make*; *Someone laughed the boy vs *Someone made the boy laugh*). The resulting activation level of the corresponding output units was taken as the model’s “grammaticality judgment” for that form. These judgments were then correlated against those obtained from native speakers of each language ( *N*=48 at each of ages 5–6, 9–10 and adults).

In general, the model achieved correlations of around *r*=0.75 with human judgments, showing only a small decrement in performance (i.e., slightly lower correlations) when tested on verbs that had been withheld during training, using split-half validation. This finding demonstrates that the model, like human learners, eventually reaches a point at which it is able to produce the appropriate causative form for verbs that it is encountering for the first time, on the basis of their semantics. Importantly, prior to this point, the model displays an “overgeneralization” stage analogous to that shown by children (at least for English). For example, when presented with *laugh*, the English model initially produces the transitive causative construction (e.g., *Someone laughed the boy) with considerably higher probability than the periphrastic causative (e.g., *Someone made the boy laugh*). After around 12 epochs of training (each consisting of 10,000 corpus utterances) the probabilities begin to flip, and the model asymptotes at predictions of around 0.7 *vs* 0.3 for the periphrastic- versus transitive-causative respectively (“Other” uses are around zero, since the model is interrogated for a causative form).

 While these findings constitute support for the model developed by Ambridge *et al.* (2020), this support is currently limited, since the model was assessed only on its ability to predict grammaticality judgment data obtained from older children (5–6 and 9–10 years) and adults. However, the available English data (e.g., Ambridge & Ambridge, 2020; Pinker, 1989; Bowerman, 1988) suggest that the majority of such overgeneralization errors are produced before this age. Indeed, for languages other than English, there is no more than anecdotal evidence that children produce such errors at all (either at age 5–6 or younger).

 The present study therefore has two aims. The first is to test the ability of the computational model developed by Ambridge *et al.* (2020) to explain grammaticality judgment data from younger children than those tested previously; children aged 4;0-5;0, which necessitates the use of a binary judgment task (rather than the Likert-scale task used with children aged 5;6-6;6). The second aim is to test the ability of this computational model to explain children’s production data, including possible overgeneralization errors, at ages 4;0-5;0 and 5;6-6;6 (for comparability with the present judgment study and that of Ambridge *et al.*, 2020, respectively).

### Ethics statement

For both Study 1 and Study 2, ethics approval was obtained from the University of Liverpool (approval number RETH001041), as the institution with overall responsibility for the project, and from local ethics committees at the Hebrew University of Jerusalem (22032020), the International Institute of Information Technology Hyderabad (IIITH/IEC/2016/1), and the Universidad del Valle de Guatemala (¿Cómo los niños adquieran la estructura de oraciones en K’iche’?). Japanese universities do not routinely provide ethics review for psychological or linguistic research. In lieu, we therefore obtained a review from Shunzo Majima, Associate Professor at the Center for Applied Ethics and Philosophy, Hokkaido University. Parents/caregivers gave informed written consent on behalf of their children, who provided verbal assent. Written consent included both participation in the study and inclusion of the data in an anonymized publicly-available dataset.

## Study 1: Binary grammaticality judgments (4;0-5;0)

### Methods

**Preregistration.** The sample size, materials, data collection methods and analysis plan were pre-registered at https://osf.io/qhnjk, on 15 ^th^ May 2018, before data collection began. We deviate here from our planned data analysis plan, which was designed to constitute separate tests of the preemption, entrenchment and verb semantics hypothesis. In our view, such an analysis is no longer meaningful, given that (a) Ambridge *et al.* (2020) reported extremely high levels of collinearity between the preemption and entrenchment predictors ( *r*=0.75-0.96 for difference scores, depending on the language) and (b) our goal is now to test the computational model of Ambridge *et al.* (2020) which collapses the distinction between preemption, entrenchment and verb semantics into a single learning mechanism. That said, the analyses we report are “pre-registered” in the sense that they correspond directly to those reported in the computational modeling section of Ambridge *et al.* (2020); the only difference being that the by-verb predictor variable averages across participants’ binary grammaticality judgments (Study 1) or binary production data (Study 2), rather than continuous grammaticality judgments. As such, other than the decision to switch to these analyses in the first place, we have retained no researcher degrees of freedom ( Wicherts *et al.*, 2016). To be explicit, we are not switching our analysis plan because the original plan failed to yield a particular pattern of results: We have not conducted the analyses specified in the original analysis plan.

**Computational model.** The model architecture was identical to that reported in Ambridge *et al.* (2020; see the present Introduction for a brief outline), though new model runs were conducted (48 runs for each of 50 epochs, for each language, as in Ambridge *et al.*, 2020).

**Participants.** Our preregistered analysis plan said that we would recruit 48 children aged 4;0-5;0 for each language: English, Hebrew, Hindi, Japanese and K’iche’. We achieved this target for every language except K’iche’ ( *N*=32), for which testing had to be terminated early due to the coronavirus pandemic. All children were native learners of the relevant language, although many would have had some limited exposure to English (particularly the Hindi-speakers) and – for K’iche’ speakers – Spanish. The target sample of *N*=48 per language was specified in the initial grant application, but was arrived at informally on the basis of the first author’s previous work, not a power calculation. Children were recruited via schools/nurseries in the UK, Israel, India, Japan and Guatemala. Parents/caregivers were sent an invitation letter and consent form. Parents/caregivers were asked not to volunteer if their children had any known or suspected language difficulties, or were not native learners of the relevant language.

**Stimuli and materials.** The sentences used in the grammaticality judgment task, along with the animations used to illustrate their intended meanings, were identical to those used in Ambridge *et al.* (2020), to which the reader is referred for a detailed description. The full set of sentences for each language can be viewed at https://osf.io/84qjh/, and the accompanying animations at https://osf.io/x6hyw/. Each sentence included either the more- or less-transparent causative form of one of the standardized set of 60 verbs (i.e., translational equivalents across languages) used in Ambridge *et al.* (2020), always with “Someone” as the causal subject (e.g., Someone made the boy laugh; *Someone laughed the boy). Further examples, for the verb laugh, are shown for each language in Table 2. The accompanying animations depicted the caused event, but not the causer, who was obscured using stage curtains. For example, for the sentences shown in Table 2, the animation depicted a boy alone on a stage; the curtains then closed and reopened to show the boy laughing.

**Table 2.** Less-transparent and more-transparent causative sentences for the verb LAUGH for each language. For the more-transparent causative, the overt causative marker is shown in bold type.

	Less-transparent causative	More-transparent causative
English	*Someone laughed the boy	Someone **made** the boy laugh
Hebrew	*Mishehu caxak et ha-yeled	Mishehu **hi**cx **i**k et ha-yeled
Hindi	*kisii=ne laRke=ko hããs-aa	kisii=ne laRke=ko hãs- **aa**-yaa
Japanese	Dareka ga otokonoko o warawasu	Dareka ga otokonoko o waraw **ase**ru
K’iche’	x-0-u-tze'-j le ak'al le achi	x-0-u-tze'n- **isa**-j le ak'al le achi

**Procedure.** Data were collected between January 2018 and March 2020 in schools and nurseries in the UK, Israel, India, Japan and Guatemala. Because the full set of 120 judgments would have been too onerous for young children, each child completed 60 judgments – more- and less-transparent forms for each of 30 verbs – according to one of four counterbalance lists (which can be viewed at https://osf.io/hsm3b/). These 60 judgments were split into two sessions of 30, given either on different days or on the same day with a break in between. For each child, 16 (or 14) verbs were rated in both more- and less-transparent form in the same session; the remaining 14 (or 16) verbs were rated in more-transparent form in one session and less-transparent form in the other session. A video of the procedure can be found at https://osf.io/fqyps/.

 The procedure, which involved the child placing a small animal toy on a green tick or a red cross, indicating “grammatical” and “ungrammatical”, respectively ( Theakston, 2004), is best summarized by the instructions that were given to children (in translation):

We are going to play a game. This dog is trying to learn to speak English (/Hindi etc.). So, we’re going to watch some short videos, and he’s going to tell us what’s happening. We have to help him by telling him when he says it right, and when he gets it wrong and says it a bit funny. In the game, we will watch a cartoon and the dog will tell us what happens. We have to listen to the dog and then if he says something that sounds okay we put the toy on the tick **[demonstrates to child]** and if he says something that sounds a bit silly then we put the toy on the cross **[demonstrates to child, then completes practice trials 1 (tick) and 2 (cross). Child completes practice trials 3 (tick) and 4 (cross)]**. We’re going to play the game again, but this time the cartoons are going to look a bit different **[shows still of animation]**. They’re going to have either this little boy or something else on this stage. These big red curtains are going to close, and you have to imagine that there is someone is behind the curtains and that person is going to do something to make something change, so that when the curtains reopen you can see how its changed. So, let’s see how this one changes. **[plays example animation:** ***dress*]**. So as you can see, in this cartoon the person behind the curtains has done something to help or make the boy get dressed. So, when we play the game again all the sentences our dog is going to say are going to start with someone and that is who the someone is, the person behind the curtains. But we’re going to play the game the same where we watch the cartoon, the dog says the sentence and we listen and then we put the toy on the tick if it sounds okay or the cross if it sounds a bit silly. You’ve also got this grid. To win the game you need to fill all these boxes with a sticker. You’ll get a sticker every time you hear this sound **[plays dog barking sound effect]**. Once there is a sticker in all of the boxes you win.

The practice trials referred to are (1) *The cat drank the milk*, (2) *The dog the ball played with, (3) *The frog caught the fly*, (4) *His teeth the man brushed (or sentences with equivalent word order errors in the other languages). The example animation with *dress* was created solely for use as an example, and did not appear in the main stimulus set (or in Study 2). The barking sound effect was automatically triggered by the software displaying the animations (PsychoPy 2; Peirce *et al.*, 2019), such that the child completed her grid and won the game on the final trial of each day. The experimenter also used this software to record the child’s response for each trial (grammatical, ungrammatical, equivocal/refused to answer). Responses of the latter type, which were very rare, were discarded for all statistical analyses.

**Analysis.** All analyses were conducted in *R* (version 3.6.3; R Core Team, 2020). All computational models were built using the *nnet* package (version 7.3-14; Venables & Ripley, 2002). Correlations were conducted using the *cor* function of base R. All plots were made using *ggplot2* (version 2.2.1; Wickham, 2016).

### Results: Binary grammaticality judgments (4;0-5;0)

Before proceeding to test the computational model, it is instructive to compare children’s binary judgment data against the gold-standard adult continuous judgment data reported by Ambridge *et al.* (2020) in order to determine (a) whether children aged 4;0-5;0 give meaningful judgments and (b) whether they make judgments that correspond to overgeneralization errors, rating as “acceptable” sentences that receive low acceptability ratings from adults.

**Figure 1.**
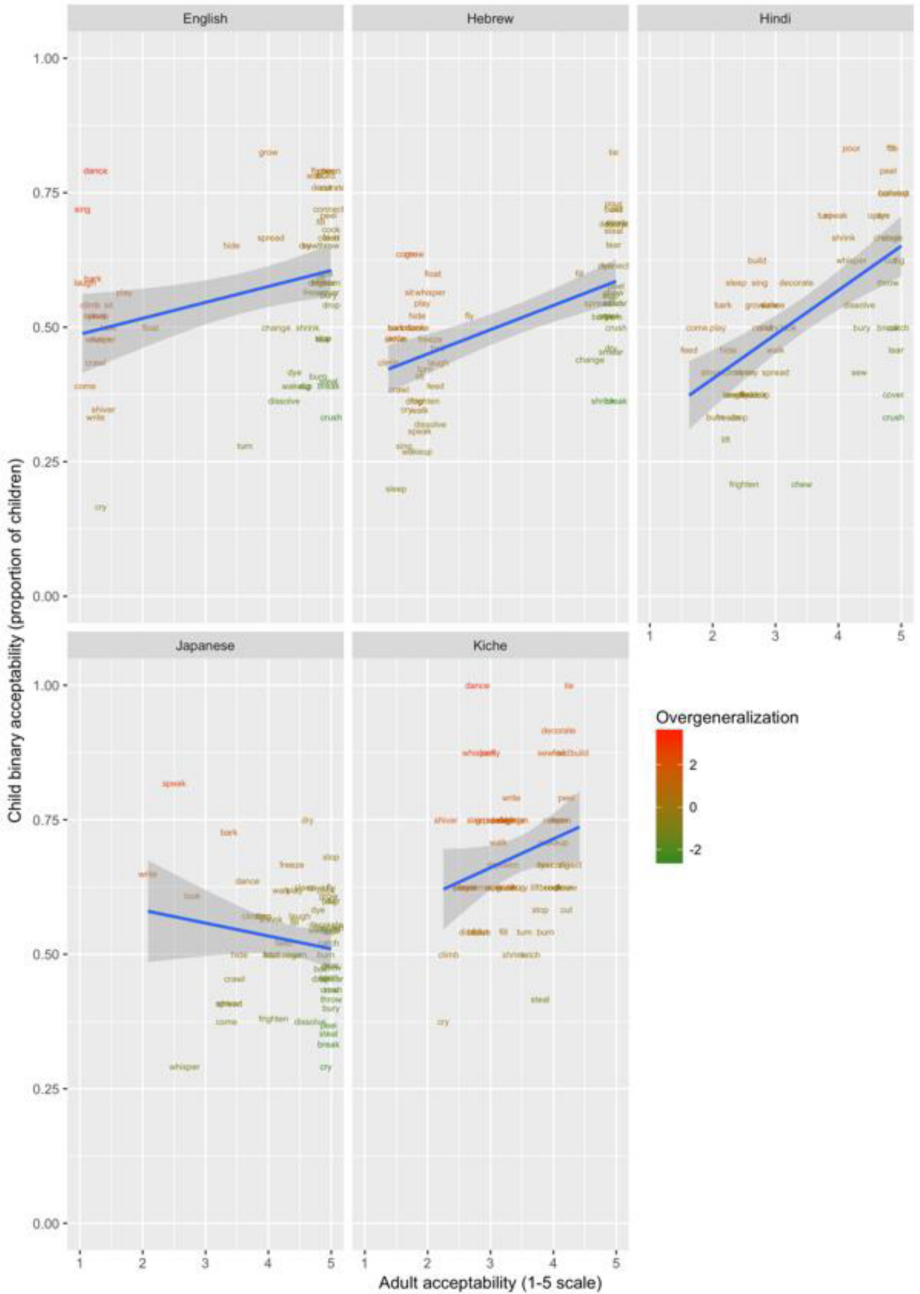
Child binary judgments (present study) versus adult continuous judgments for less-transparent forms.

 These data are plotted in Figure 1– Figure 3 for less-transparent forms (e.g., *Someone laughed the boy), more-transparent forms (e.g., *Someone made the boy laugh*) and difference scores (less- minus more-transparent forms), respectively. The x-axis shows, for each verb form, the mean acceptability rating given by adults on the five-point scale. The y-axis shows, for each verb form, the proportion of children accepting that form (recall that each child makes only a single binary acceptability judgment for each form). Forms are colour coded to indicate child judgments that correspond to “overgeneralization errors” at the group level. This was done by converting by-verb mean adult acceptability judgments and by-verb child acceptability proportions into Z-scores, and subtracting the former from the latter (or vice-versa for the difference scores, where smaller scores correspond to overgeneralization). A large positive score (red) represents overgeneralization. For example, in Figure 3 (less-transparent forms), English *dance* and *sing* are red, since around 75% of children deemed *Someone danced the boy and *Someone sang the boy to be acceptable, despite the fact that adults assigned mean acceptability ratings close to the minimum possible (1/5) for both. A large negative score (green) represents undergeneralization. For example, in Figure 3 (less-transparent forms), English *break* and *crush* are green, since only around 30–40% of children deemed *Someone broke the truck* and *Someone crushed the can to be acceptable*. Informally, the researchers who worked with the children reported that this is probably due to children rating sentences, to some extent, on the basis of the social desirability of the events described.

**Figure 2.**
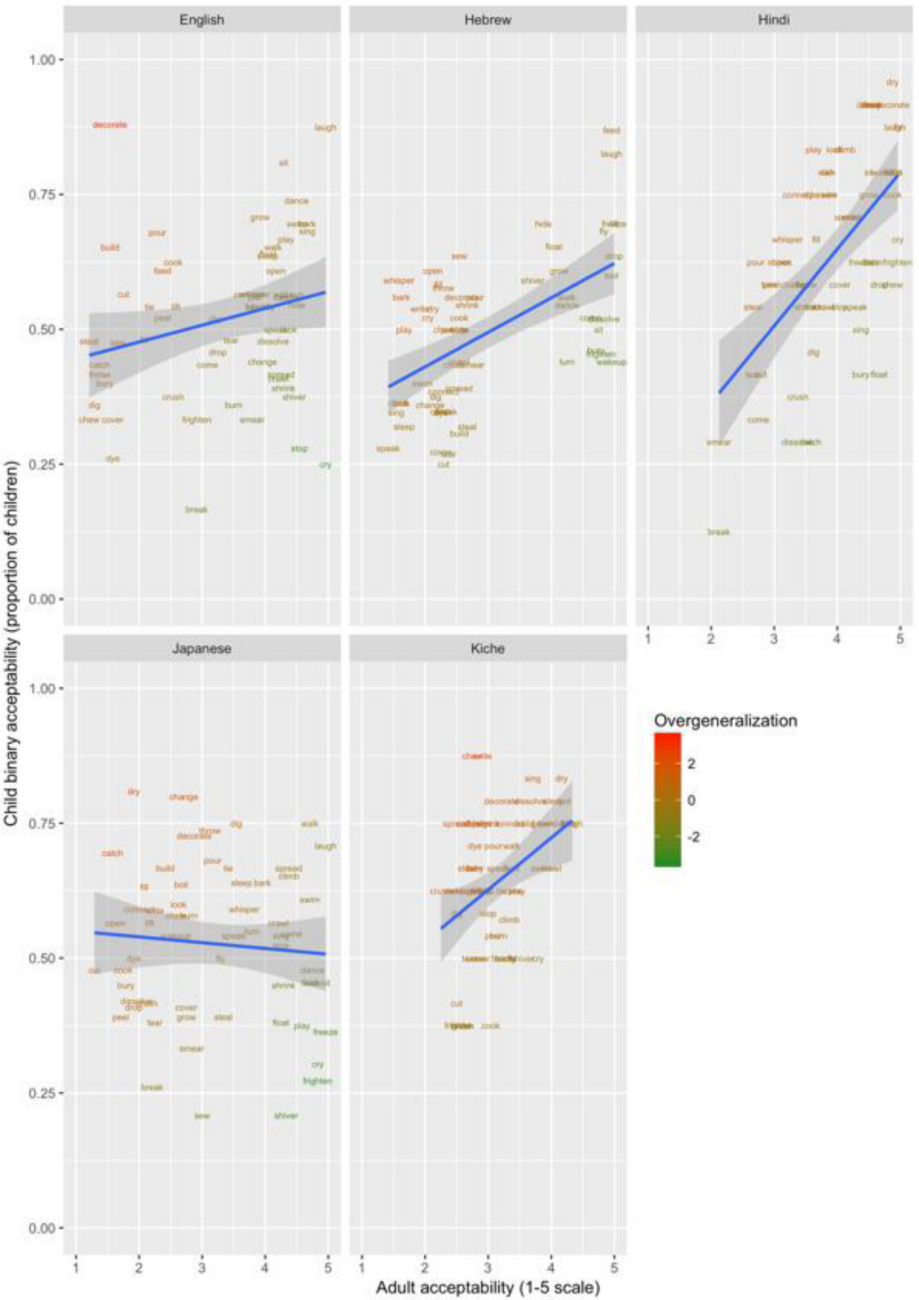
Child binary judgments (present study) versus adult continuous judgments for more-transparent forms.

**Figure 3.**
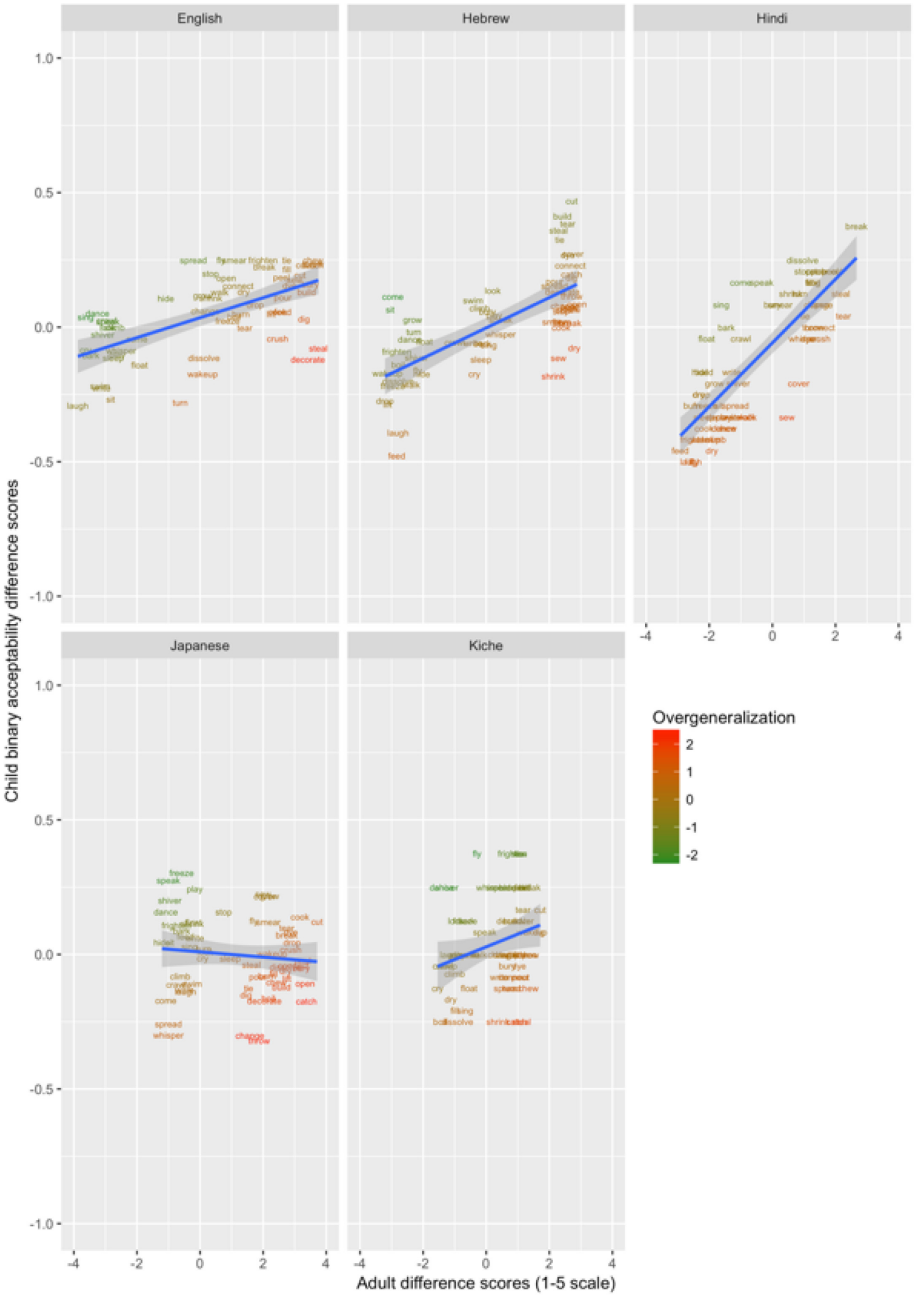
Child binary judgments (present study) versus adult continuous judgments for difference scores (less- minus more-transparent).

 Such effects – as well as any other idiosyncratic (dis)preferences for particular verbs – are washed out by the difference scores, since the less- and more-transparent forms are matched for social desirability (and for the animation illustrating the event). Inspection of these scores ( Figure 3) indicates that children’s judgments in fact mirrored adults’ judgments quite closely, with relatively few clear cases of overgeneralization (corresponding here to a smaller difference score for children than adults).

 In order to verify that, despite some evidence of over- and under-generalization errors, children’s judgments generally mirrored those of adults, we conducted Pearson correlations on the means for each verb, corresponding to those plotted in Figure 1– Figure 3 (see Table 3).

**Table 3.** By-verb correlations between child binary grammaticality judgments (mean proportion of children accepting each form) and adult continuous grammaticality judgments (rating on five-point scale; from Ambridge *et al.*, 2020). Significant correlations are shown in bold.

	English	Hebrew	Hindi	Japanese	K’iche’
Less transparent	**0.31**	**0.55**	**0.59**	-0.16	**0.24**
More transparent	**0.24**	**0.57**	**0.60**	-0.08	**0.40**
Difference scores	**0.61**	**0.68**	**0.80**	-0.10	**0.26**

These data suggest that, at least for English-, Hebrew-, Hindi- and K’iche’-speaking, children were indeed giving meaningful judgments. The Japanese-speaking children, however, displayed an anomalous pattern of judgments, rating as unacceptable many forms that are highly acceptable to both experimentally-tested adults ( Ambridge *et al.*, 2020) and, informally, to the present native-Japanese-speaking co-authors. Although the majority of child participants were tested in a different area of Japan to the adults tested by Ambridge *et al.* (2020) (Fukuyama City, Hiroshima, as opposed to Tokyo), we are not aware of any relevant dialectal differences. A possible cause of this anomalous pattern is that children are basing their ratings on social desirability, with many of the forms that they deemed unacceptable denoting undesirable actions (e.g., the less transparent forms for *cry, break, steal, dissolve, bury, throw*, see Figure 1, and the more-transparent forms for *shiver, frighten, cry* and *freeze*, see Figure 2). As noted above, informally, the experimenters observed this problem to some extent across all languages. It is possible, however, that social desirability may be particularly salient in the more collectivist Japanese culture (e.g., Johnson & Van de Vijver, 2003). However, social desirability alone cannot explain why the anomalous pattern holds even for difference scores which control for such by-verb effects.

**Figure 4.**
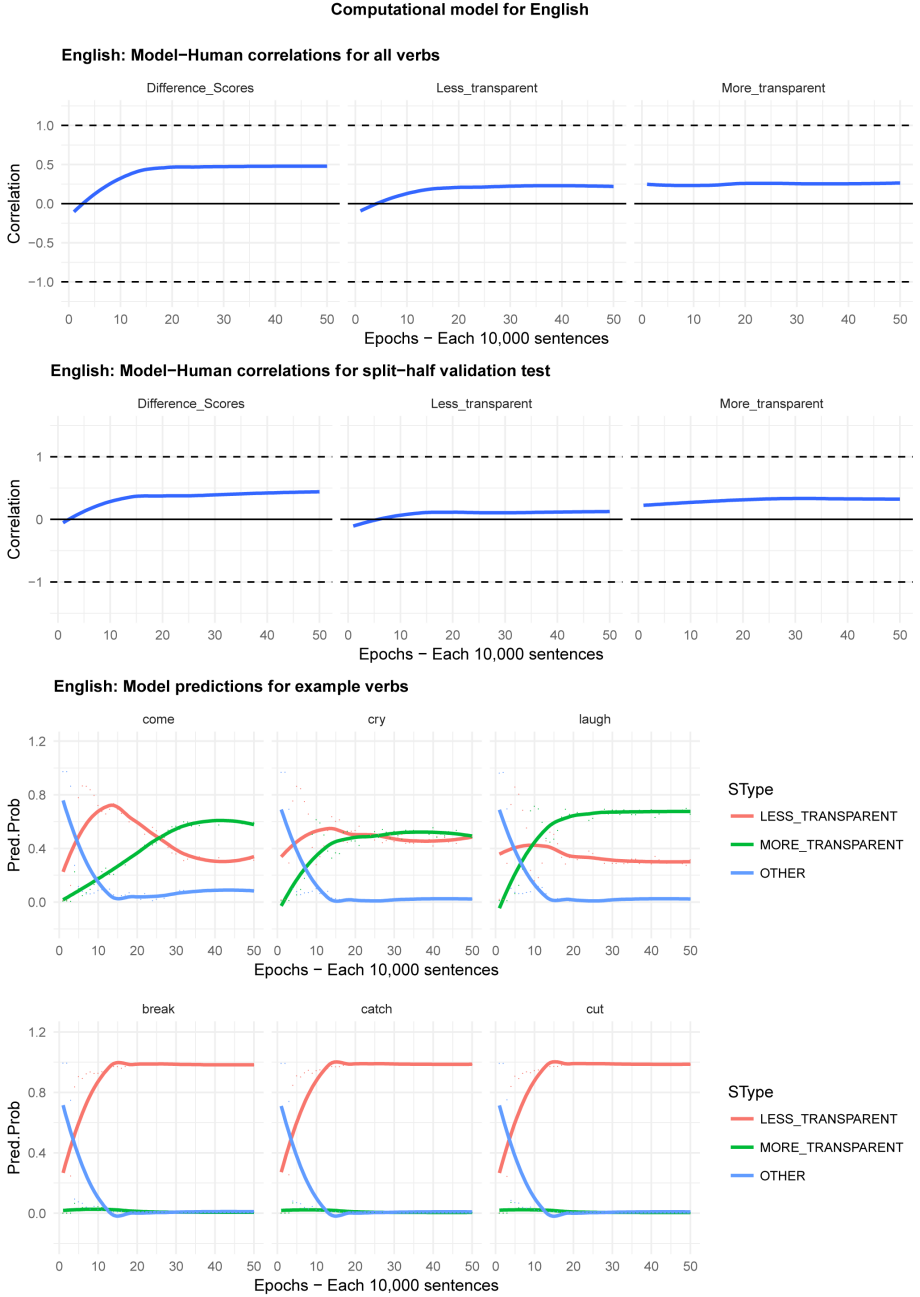
Model-child correlations for English binary judgment data.

 Moving on to the tests of the computational model, Figure 4– Figure 8 plot – for English, Hebrew, Hindi, Japanese and K’iche’, respectively – model-child correlations for (a) the full set of 60 verbs, and (b) the split-half validation test (30 verbs, randomly selected for each run), as well as the developmental pattern shown by the model for a number of example verbs. For children’s judgments, the dependent measure is again the proportion of children judging the particular verb form (more-/less-transparent) to be acceptable on the binary judgment task (or a less-minus-more-transparent difference score). The predictor variable is the mean activation level of the corresponding unit of the model (or a difference score calculated in the same way).

**Figure 5.**
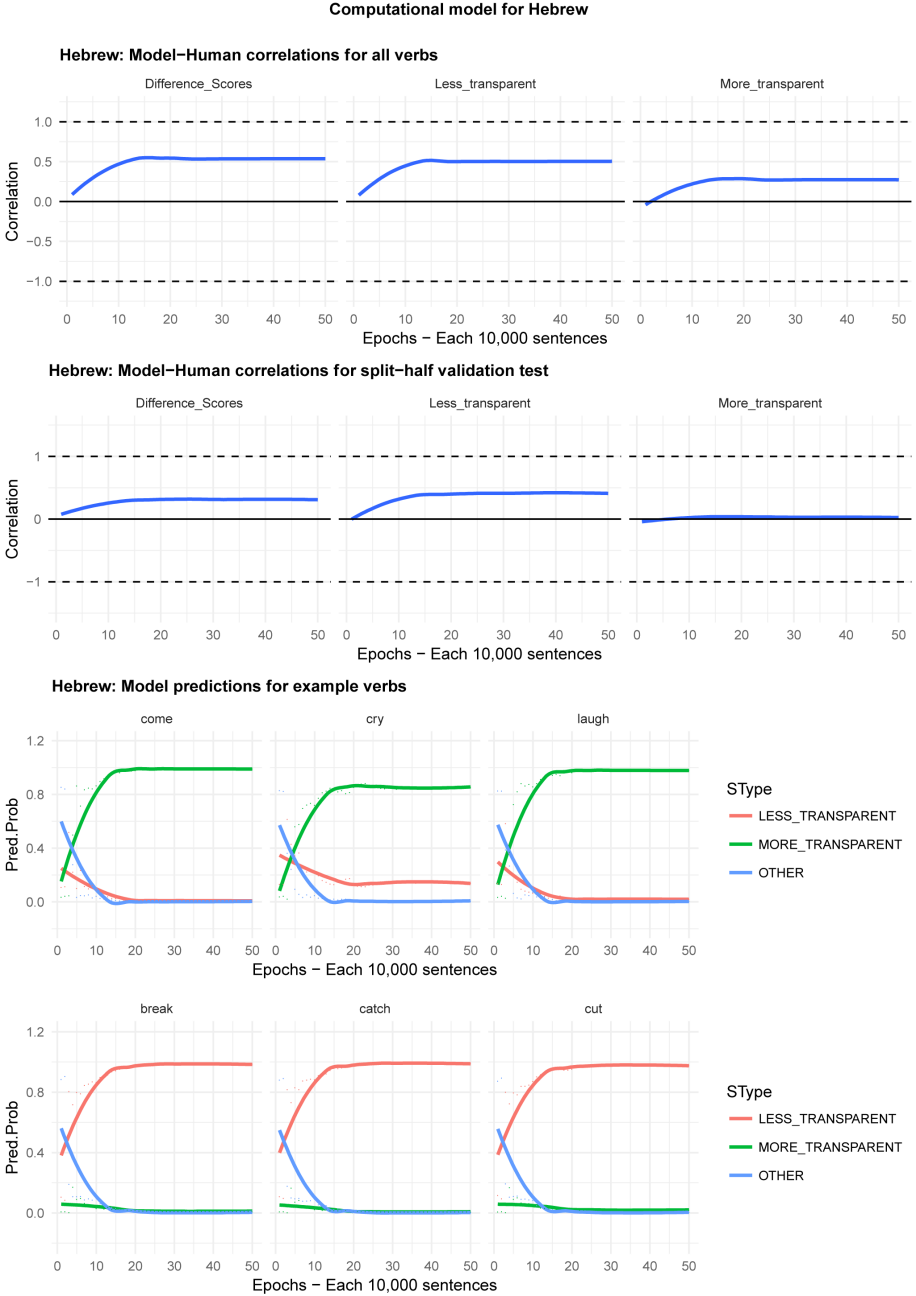
Model-child correlations for Hebrew binary judgment data.

**Figure 6.**
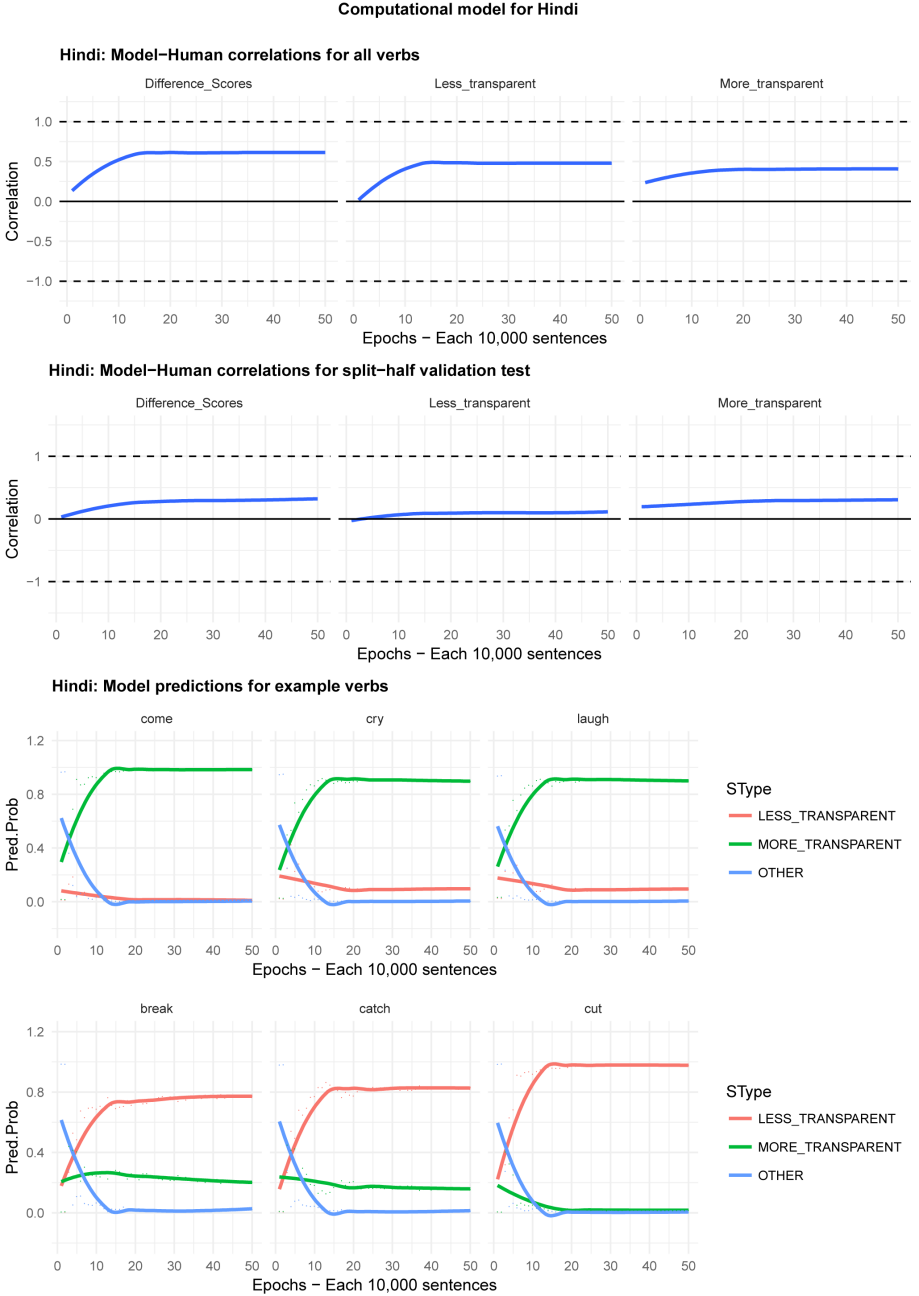
Model-child correlations for Hindi binary judgment data.

**Figure 7.**
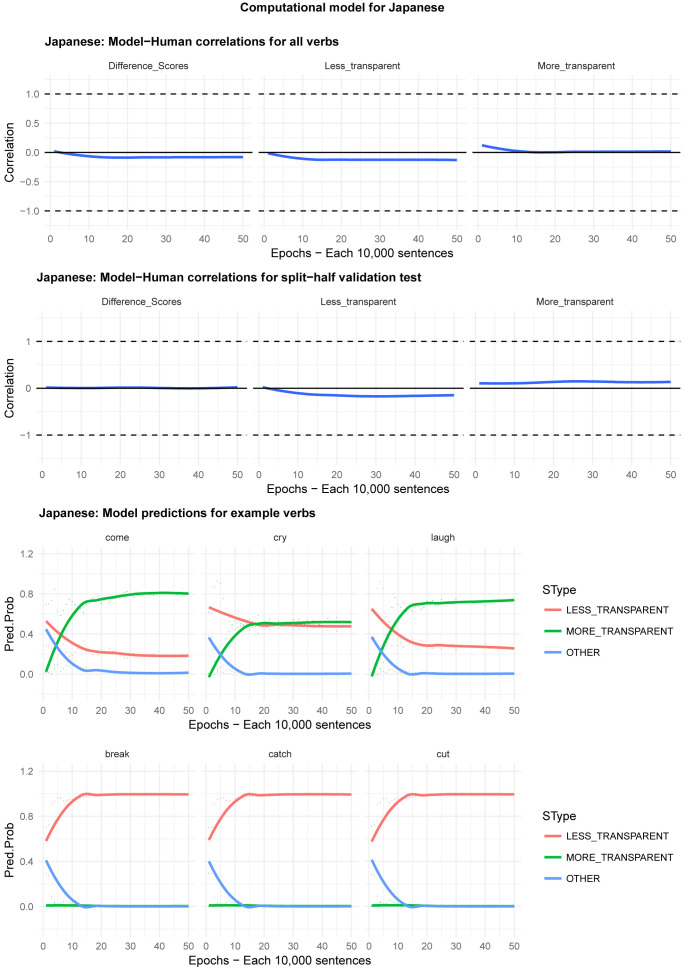
Model-child correlations for Japanese binary judgment data.

**Figure 8.**
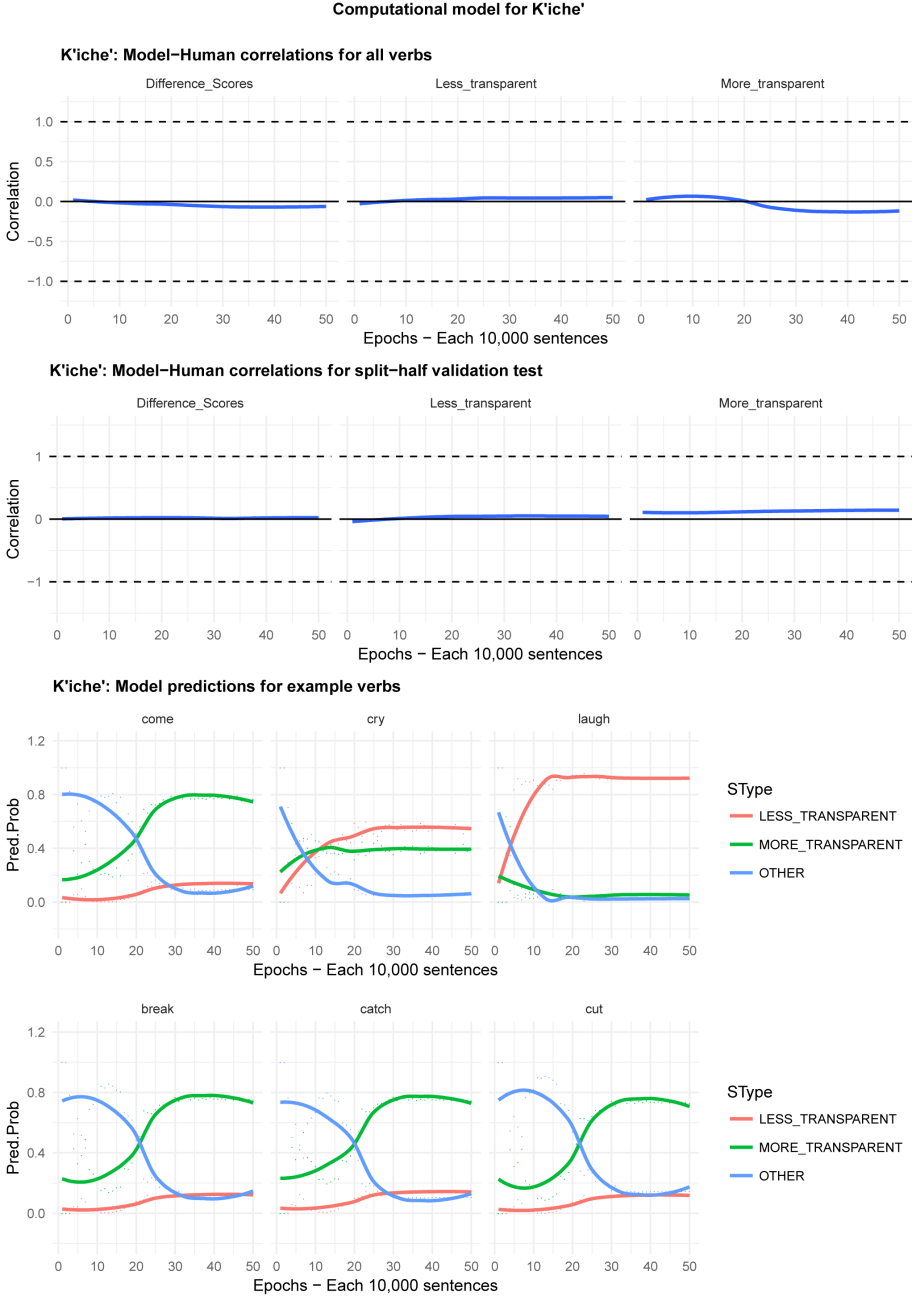
Model-child correlations for K’iche’ binary judgment data.

In general, the model does a good job of predicting children’s binary judgment data, though less so than for adults’ continuous judgment data ( Ambridge *et al.*, 2020, reported correlations mainly in the region of *r*=0.75). For the present binary judgment data, focussing on difference scores, the model achieved correlations in the region of *r*=0.5- *r*=0.6 for the English, Hebrew and Hindi child data, both for seen verbs and in the split-half validation test. All six correlations are comfortably statistically significant at *p*<0.01 (Critical *r* [df = 58] value for *p* < 0.05 = 0.21; for *p* < 0.01 = 0.30 [one tailed]). The model fares less well at predicting the raw proportions of “acceptable” judgments for less- and more- transparent causative forms; though with *r* values in the region of *r*=0.25- *r*=0.5, all twelve correlations are again statistically significant.

 For Japanese and K’iche’ the model achieves only one significant correlation, for more-transparent causative forms in Japanese. The poor performance of the K’iche’ model was to be expected on the basis of Ambridge *et al.* (2020) who found similar results for adults, which they attributed to difficulties with obtaining reliable corpus counts and semantic ratings. The poor performance of the Japanese model probably reflects the fact that – as noted above – Japanese children showed the nosiest performance on the judgment task and, indeed, no significant correlation with adult judgments (possibly due to increased social-desirability effects).

### Discussion: Binary grammaticality judgments (4;0-5;0)

Data from the binary judgment task show that, with the apparent exception of Japanese, children aged 4;0-5;0 are capable of providing meaningful grammatical acceptability judgments for sentences containing more- and less-transparent causative verb forms, though they also show some evidence of judgments that correspond to overgeneralization errors (e.g., accepting *Someone danced the boy and *Someone sang the boy). These judgment overgeneralization errors correspond to production overgeneralization errors observed in children’s spontaneous speech data (e.g., Table 1). The computational model developed by Ambridge *et al.* (2020) successfully explained children’s judgment data for English, Hebrew and Hindi. Its failure to do so for K’iche’ and Japanese appears to be attributable to noise in the predictor variables and children’s judgment data respectively. These findings raise two questions: (1) Do children learning each of these languages actually produce these types of overgeneralization errors and, if so, (2) Can the computational model developed by Ambridge *et al.* (2020) explain their by-verb patterning?

## Study 2: Elicited production (4;0-5;0 and 5;6-6;6)

### Methods

**Preregistration.** As for Study 1, the sample size, materials, data collection methods and analysis plan were pre-registered at https://osf.io/qhnjk before data collection began. Again, we depart here from our data-analysis plan in order to test the computational model of Ambridge *et al.* (2020) which we judge to supersede the single-process theories tested in our original pre-registration.

**Computational model.** As for Study 1, the model architecture was identical to that reported in Ambridge *et al.* (2020) though new model runs were conducted (again, 48 runs for each of 50 epochs, for each language).

**Participants.** As per our preregistration, we recruited 48 children at each of ages 4;0-5;0 and 5;6-6;6 for each language (including K’iche’). Children were recruited from the same populations as Study 1, though none took part in both studies. Sample size criteria, eligibility criteria, and sources and methods of participant selection were the same as for Study 1.

**Stimuli and materials**. This study used a priming methodology, in order to encourage children to attempt to produce both less- and more-transparent causative forms for each of 60 target verbs (the same set used in Study 1 and Ambridge *et al.*, 2020). For each language, a further 60 verbs – 30 each that prefer the more- and less-transparent causative form – were selected for use as prime verbs, and corresponding animations created (following the same format as the animations for the target verbs). Only 60 prime verb were necessary, because – as for Study 1 – each child completed only half of the total number of target trials: That is, for each of 30 verbs – according to eight counterbalance lists – children described a causal animation following priming with (a) a more-transparent causative and (b) a less-transparent causative. As for Study 1, children completed two separate sessions. For each child, 16 (or 14) of the verbs appeared following both more- and less-transparent causative primes in the same session; the remaining 16 (or 14) appeared following a more-transparent causative prime in one session and a less-transparent causative prime in the other.

**Procedure.** Data were collected between January 2018 and March 2020 in schools and nurseries in the UK, Israel, India, Japan and Guatemala. A video of the production priming procedure can be found at https://osf.io/hqr9p/. Again, the procedure, is best summarized by the instructions that were given to children (in translation):

We are going to play a game. We’re going to watch some short videos and take it in turns telling this dog what has happened. The dog has either my card or your card: If we hear this sound **[plays howl sound effect]** then he has mine, if we hear this **[plays bark sound effect]** then he has yours. Then we can put our card on the grid and whoever fills their grid first wins the whole game. Our videos are going to look a bit like this. There is a stage like one you would see in a theatre with big red curtains **[plays an example animation:** ***dress*]**. So, as you can see, there was a little boy on the stage and he has no top on **[shows still of the stage at the beginning]** and when the curtains reopened he had a top on **[shows still of the stage at the end**]. You must imagine that when the curtains are closed that there is someone behind the curtains **[shows the closed curtains]**. So, in this one there was someone behind the curtains that did something to get the boy dressed. Let’s start with some practice ones and I’ll help you:
**Practice trial 1 – (** ***dress*** **and** ***wrap*)**
Experimenter: “someone dressed the boy”
Experimenter: “someone wrapped the present” **[encourages child to repeat]**
**Practice trial 2 – (** ***hiccup*** **and** ***jump*)**
Experimenter: “someone made the boy hiccup”
Experimenter: “someone made the boy jump” **[encourages child to repeat]**
**Practice trial 3 – (** ***free*** **and** ***close*)**
Experimenter: “someone freed the boy” **[waits for/encourages child to produce…]**
Child: “Someone closed the door” **[experimenter corrects if necessary]**
**Practice trial 4 – (** ***burp*** **and** ***drink*)**
**Experimenter**: “someone made the boy burp” **[waits for/encourages child to produce…]**
Child: “someone made the boy drink” **[experimenter corrects if necessary]**

The child and experimenter then completed the test trials in the same way. Note that the training trials were designed to give the child practice at producing less- and more-transparent causative forms following less- and more-transparent causative primes respectively. As for Study 1, the training verbs/animations did not feature in the test trials, and the barking/howling sound effects were automatically triggered by the software displaying the animations (Processing 2; https://processing.org/), such that the child completed her grid and won the game on the final trial of each day. Children’s responses were coded as to whether they included a more-transparent or less-transparent form of the target verb, with all other responses (e.g., intransitive use of the target verb; use of a different verb; no response) treated as missing data.

**Analysis.** All analyses were conducted in *R* (version 3.6.3; R Core Team, 2020). All computational models were built using the *nnet* package (version 7.3-14; Venables & Ripley, 2002). Correlations were conducted using the *cor* function of base R. All plots were made using *ggplot2* (version 2.2.1; Wickham, 2016).

### Results: Elicited production (4;0-5;0 and 5;6-6;6)

As for Study 1, before proceeding to test the computational model, it is instructive to compare children’s data against the gold-standard adult continuous judgment data reported by Ambridge *et al.* (2020) in order to determine (a) whether children’s productions generally seem to follow the constraints of the adult grammar and (b) whether they nevertheless produce overgeneralization errors that correspond to those observed (for English) in naturalistic data.

**Figure 9.**
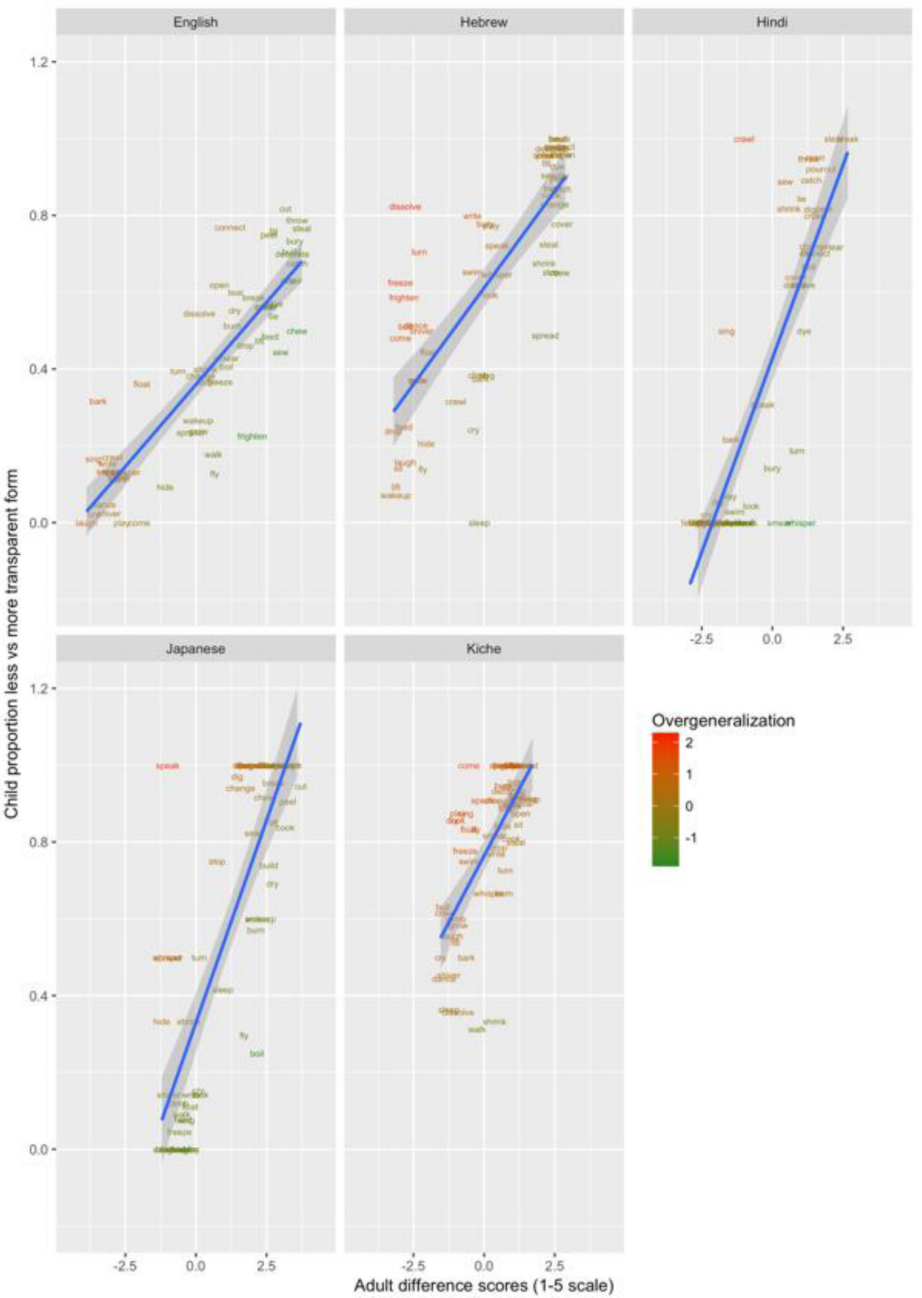
Children’s (4;0-5;0) elicited productions (present study) versus adult continuous judgments.

**Figure 10.**
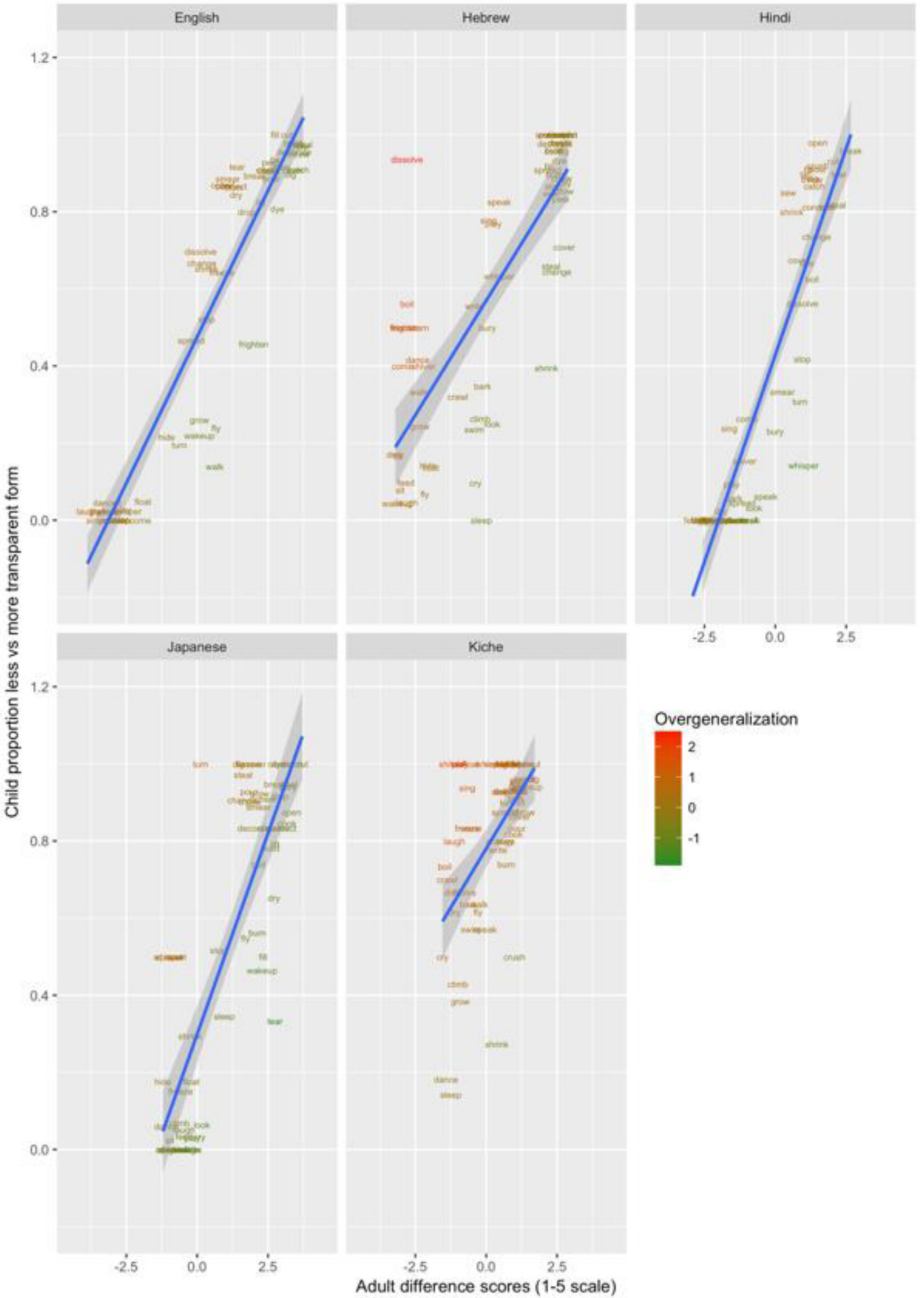
Children’s (5;6-6;6) elicited productions (present study) versus adult continuous judgments.

These data are plotted in Figure 9 (children aged 4;0-50) and Figure 10 (children aged 5;6-6;6). The x-axis shows, for each verb form, adults’ mean difference score (preference for less-over more-transparent causative forms). The y-axis shows the proportion of trials on which children, as a group, produced the less- versus more-transparent causative form of each verb (recall that all other responses were discarded as missing data).

Overgeneralization errors, this time in production, are colour coded in the same way as for Study 1. Learners of all five languages show evidence of making overgeneralization errors at relatively high rates, almost exclusively by producing less-transparent causative forms for verbs that strongly prefer more-transparent causative forms. This asymmetry is also observed for English naturalistic data (see Table 1; Bowerman, 1988) and is simulated by the computational model reported in Ambridge *et al.* (2020). The cause for the model (and, presumably, children) is that less-transparent causative forms are far more frequent in children’s input). For example, English-speaking 4–5 year-olds produced * *Someone barked the dog* and * *Someone sang / crawled / wrote / whispered / sang / slept / sat the boy* (c.f., *Someone made the boy/dog bark / sing / crawl* etc.) at rates of 10–30%. Hebrew-speaking 4–5-year-olds produced corresponding errors for *dissolve, turn, freeze, frighten, dance, shiver* and *come* at rates of 40–80%, perhaps reflecting the fact that the Hebrew binyan system is, in general, relatively productive. Hindi- and Japanese-speaking 4–5-year-olds produced considerably fewer errors of this type, though still a handful (e.g., for *crawl, sing; speak, whisper* and *hide*). K’iche-speaking 4–5 year olds produced very high rates of apparent overgeneralization errors, but note from Figure 9 that the K’iche’ speaking adults show much smaller difference scores than adult speakers of the other languages. That is, while K’iche-speaking 4–5-year-olds produce less-transparent causative forms of *come, speak, play, look* and *float* more often than would be expected on the basis of adult grammatical acceptability judgments, those same judgments suggest that these forms are not strongly unacceptable.

 Comparison of Figure 9 (4;0-5;0) and Figure 10 (5;6-6;6) indicates that, by this later age, overgeneralization errors have all but ceased for English, Hindi and Japanese, and decreased considerably for Hebrew. Only for K’iche’ do rates remain high, probably reflecting the fact that the dispreferred forms are not in fact deemed highly unacceptable by adults. Importantly, the productions of both the younger and older groups show significant correlations with adult rating data (see Table 4), suggesting that children understand the task and, despite the presence of some overgeneralization errors, are largely producing appropriate responses (Critical *r* [df = 58] value for *p* < 0.05 = 0.21; for *p* < 0.01 = 0.30 [one tailed]).

**Table 4.** By-verb correlations between children’s production (mean proportion of less- *vs* more-transparent causative forms) and adult continuous grammaticality judgments (rating on five-point scale; from Ambridge *et al.*, 2020). Significant correlations are shown in bold.

	English	Hebrew	Hindi	Japanese	Kiche
Age 4–5	**0.87**	**0.78**	**0.84**	**0.81**	**0.68**
Age 5–6	**0.94**	**0.79**	**0.91**	**0.82**	**0.55**

**Figure 11.**
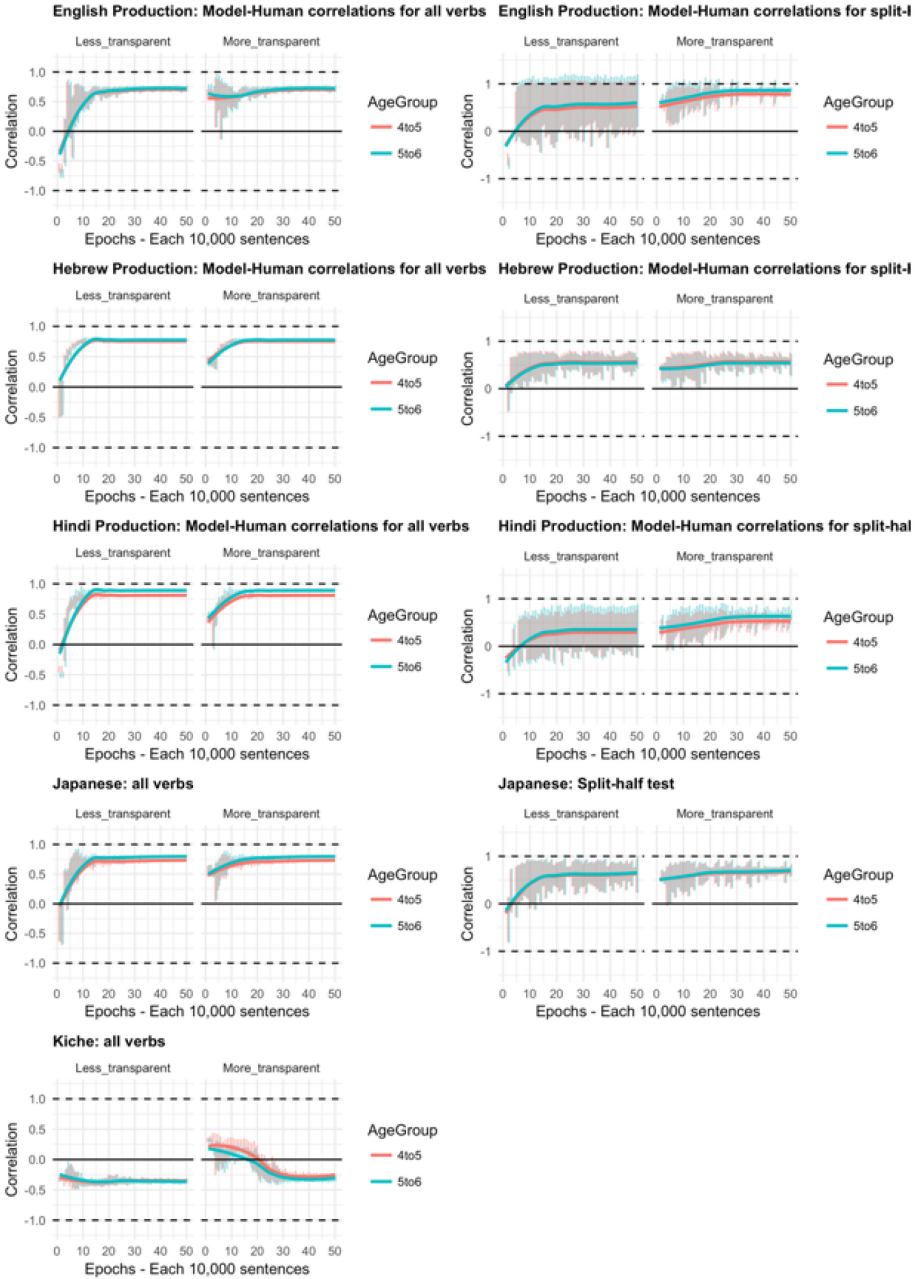
Model-child correlations for elicited production data.

Moving on to the tests of the computational model, Figure 11 plots – for English, Hebrew, Hindi, Japanese and K’iche’ respectively – model-child correlations for (a) the full set of 60 verbs, and (b) the split-half validation test (30 verbs, randomly selected for each run), as well as the developmental pattern shown by the model for a number of example verbs. Separate correlations are run for less-transparent and more-transparent causative forms because, although these sum to 1 for children (since all other responses are treated as missing data), the same is not true for the model which has three output units, corresponding to less-transparent, more-transparent and “Other”. That said, since the model rapidly learns to predict “Other” forms with very low probability when interrogated for a causative form, the correlations for less- and more- transparent forms are extremely similar.

 For all languages except K’iche’, the model does an excellent job of predicting children’s judgment data with correlations upwards of *r*=0.75 for seen verbs, and *r*=0.5 for unseen verbs. Again, its poor performance with K’iche’ is likely attributable to difficulties with obtaining reliable corpus counts and semantic ratings ( Ambridge *et al.*, 2020). For this reason, we did not proceed to the split-half validation test for K’iche’. For the four other languages, however, the model’s ability at predicting children’s production data is on a par with its ability at predicting adults’ continuous judgment data ( Ambridge *et al.*, 2020). The only notable shortcoming of the model is that although it simulates the overall generalization-then-retreat pattern shown by children (see Figure 4– Figure 8, bottom panels), it does not simulate the observed differences between the present 4;0-5;0 and 5;6-6;6 year olds (see Figure 9– Figure 10). That is, the model does not show an “immature” stage in which its predictions correspond more closely to the productions of the younger than the older children. This may be because the main difference between 4;0-5;0 and 5;6-6;6 year olds is simply an across-the-board decrease in the production of overgeneralization errors, rather than any change in their by-verb patterning. Indeed, other than for forms that show floor or ceiling effects (100% or 0% less- *vs* more-transparent forms), an across-the-board decrease in errors that applied equally to all verbs would not affect the magnitude of the correlation.

### Discussion: Elicited production (4;0-5;0 and 5;6-6;6)

Data from the elicited-production task show that, with the exception of K’iche’, children aged 4;0-5;0 and 5;6-6;6 not only produce causative overgeneralization errors (* *Someone sang / crawled / wrote / whispered / sang / slept / sat the boy*; c.f., *Someone made the boy/dog bark / sing / crawl* etc.) but do so in such a way that their by-verb patterning is well predicted by the computational model of Ambridge *et al.* (2020).

## General discussion

The question of how language learners (eventually) come to avoid the production of verb argument structure overgeneralization errors ( *The clown laughed the man) has long been seen as one that is both particularly central to acquisition research and particularly challenging ( Bowerman, 1988; Pinker, 1989). Focussing on causative overgeneralization errors of this type, Ambridge *et al.* (2020) built a computational model that learns, on the basis of corpus data and human-derived verb-semantic-feature ratings, to predict adults’ by-verb preferences for less- versus more-transparent causative forms (e.g, * *The clown laughed the man* vs *The clown made the man laugh*) across English, Hebrew, Hindi, Japanese and – to a lesser extent – K’iche. The aim of the present study was to investigate whether children learning these languages indeed produce such errors, and rate them as acceptable in a binary judgment task, and – if so – whether the computational model of Ambridge *et al.* (2020) can explain their patterning.

In general, the answer to of these questions is a resounding “yes”. For example, the English sentences *Someone danced the boy and *Someone sang the boy were deemed acceptable by a majority of children aged 4;0-5;0 in a binary judgment task (Study 1), and were even produced at rates of around 5% and 15% respectively by (different) children at this age, though not by children aged 5;0-5;6 (Study 2). The computational model developed by Ambridge *et al.* was able to predict the by-verb patterning of both children’s binary-judgment data (correlations in the region of *r*=0.5-0.6) and their elicited-production data (correlations upwards of *r*=0.75), as well as generalizing to unseen verbs in a split-half validation. Given that an identical model can predict (a) adults’ continuous judgment data, (b) children’s binary judgment data and (c) children’s production data – without having been trained on any of these datasets – the problem of how language learners come to appropriately constrain their argument structure generalization looks close to being solved.

 A number of issues, however, do remain. First, despite its overall successes, the model did not significantly predict Japanese children’s binary grammaticality judgments or any of the K’iche’ data (for adults and children alike). While it is possible to come up with an apparently-reasonable explanation in each case, future work should investigate the alternative possibility that the computational model tested here perhaps does not apply universally. For Japanese binary judgments, the model’s failure is almost certainly due to a task effect, since the model does successfully predict both adults’ continuous judgments and children’s production data. For K’iche’ it is less clear. Although, as already noted, both the corpus and semantic-rating data are questionable, we should not discount the possibility that this model – and the account of causatives that it instantiates – is not well suited to languages like K’iche’ that have both transitivizing and intransitivizing morphological processes. For example, in English, Hebrew, Hindi and Japanese, *laugh* is perhaps the single most prototypical example of a highly intransitive verb that strongly prefers the less-direct, more transparent causative (e.g., Someone made the boy laugh > *Someone laughed the boy). Yet in K’iche’, intransitive *laugh* is derived from the transitive (though not transitive-causative) verb *laugh at*, and is – broadly speaking – acceptable in both causative forms; the same is true for *look* (derived from *look at*) and *speak* (from *speak about*). Perhaps, then, the crosslinguistic typology of causatives embodied by the computational model tested here is not quite accurate.

 This relates to a second issue: While it is certainly impressive that the model can account for adult and child data across – K’iche’ aside – four unrelated languages; these four languages hardly constitute a large or representative sample of all the languages of the world. Future work using the methods here should investigate whether this model generalizes to other languages.

 Third, future work using related methods should investigate whether an account of this type can explain the retreat from overgeneralization for a wide variety of syntactic and morphological constructions. We see no particular reason to believe that it cannot (e.g., see Ambridge & Blything, 2016; Li & MacWhinney, 1996, for similar models of the English *un-* prefixation and dative constructions), but, of course, the outcomes of such investigations cannot be anticipated.

 Fourth, even for the restricted case of less- versus more-transparent causative forms, the model tested here does not solve the learning problem entirely, given that it starts from the point at which children have already acquired the relevant forms (e.g., the transitive-causative and *make* periphrastic causatives for English; lexical causatives and the –(s)ase causative marker for Japanese; the transitive and causative binyanim for Hebrew). Although the model learns a great deal about the meanings of these forms – i.e., the particular type of causation that is associated with each – the forms themselves are pre-given; and in most cases are highly abstract generalizations. In this respect, the account tested here is no different to all other accounts of the retreat from overgeneralization discussed in the Introduction. But until we have a model that can learn the generalizations in the first place, we cannot quite say that the problem of forming appropriately restricted generalizations has been solved.

 Finally, the present study has important methodological implications in that three different methods – continuous grammaticality judgments, binary grammaticality judgments and elicited production – have produced findings that are generally very highly correlated with one another. Indeed, we could – at a push – argue that five different methods have converged on similar conclusions, if we include both the diary data that first uncovered such errors (e.g., Bowerman, 1988; Table 1) and the corpus analysis used to derive the model’s training data. The methodological implications are – on the one hand – that triangulating different methods on the same set of stimuli provides a particularly detailed and robust test of a particular model; and – on the other – that where this is not possible, we can be reasonably confident that conclusions drawn on the basis of data collected using one method will generalize to another.

 In conclusion, while work remains to be done to extend this research to other constructions and other language families, the present findings that the computational model developed by Ambridge *et al.* (2020) explains both children’s binary grammaticality judgment and elicited production data across a range of languages suggest that a solution to the longstanding problem of the retreat from overgeneralization is within our grasp.

## Data availability

### Underlying data

Open Science Framework: CLASS: Cross Linguistic Acquisition of Sentence Structure. https://doi.org/10.17605/OSF.IO/ATUJF ( Ambridge, 2021).

This project contains the following underlying data:

BinaryJudgmentsAndProduction.Zip (Zip file containing each of the following)

Binary Modeling (Folder containing each of the following)

- Binary Modeling.R (R code for the computational modeling)
- ENG_Adults.csv – English grammaticality judgment data (from Ambridge *et al.*, 2020)
- ENG_Input.csv – English input file for the computational modeling
- ENG_Results.csv – English children’s binary judgment data – target for modeling
- HEB_Adults.csv – Hebrew grammaticality judgment data (from Ambridge *et al.*, 2020)
- HEB_Input.csv – Hebrew input file for the computational modeling
- HEB_Results.csv – Hebrew children’s binary judgment data – target for modeling
- HIN_Adults.csv – Hindi grammaticality judgment data (from Ambridge *et al.*, 2020)
- HIN_Input.csv – Hindi input file for the computational modeling
- HIN_Results.csv – Hindi children’s binary judgment data – target for modeling
- JAP_Adults.csv – Japanese grammaticality judgment data (from Ambridge *et al.*, 2020)
- JAP _Input.csv – Japanese input file for the computational modeling
- JAP _Results.csv – Japanese children’s binary judgment data – target for modeling
- KIC_Adults.csv – Kiche’ grammaticality judgment data (from Ambridge *et al.*, 2020)
- KIC_Input.csv – Kiche’ input file for the computational modeling
- KIC_Results.csv – Kiche’ children’s binary judgment data – target for modeling

Production Modeling (Folder containing each of the following)

- ENG_Input.csv – English input file for the computational modeling
- ENG_Results.csv – English children’s production data – target for modeling
- HEB_Input.csv – Hebrew input file for the computational modeling
- HEB_Results.csv – Hebrew children’s production t data – target for modeling
- HIN_Input.csv – Hindi input file for the computational modeling
- HIN_Results.csv – Hindi children’s production data – target for modeling
- JAP _Input.csv – Japanese input file for the computational modeling
- JAP _Results.csv – Japanese children’s production data – target for modeling
- KIC_Input.csv – Kiche’ input file for the computational modeling
- KIC_Results.csv – Kiche’ children’s production data – target for modeling
- Production Correlations with Old Paper.R (R files for creating Figures 1–3)

### Extended data

Open Science Framework: CLASS: Cross Linguistic Acquisition of Sentence Structure. https://doi.org/10.17605/OSF.IO/ATUJF ( Ambridge, 2021).

This project contains the following extended data:

- AAFinal_Sentence_Stimli(Version 2).xlsx (Final sentence stimuli)
- Binary grammaticality instructions1.docx (Full text of instructions given to children completing the binary judgment task)
- Binary Judgement.zip (Zip file containing all video and audio stimuli, blank participant record and key sheets, and the sticker grid completed by children)
- Binary Judgement procedure.mp4 (Video illustrating the binary judgment procedure)
- Practice animations (Folder containing practice animations for the judgment warm up)
- Child instructions production.docx (Full text of instructions given to children completing the production task)
- CausativeAnimations.zip (Zip file containing all video and audio stimuli for the production task)
- JudgmentLists.zip (Zip file containing the different counterbalance lists for each language)
- Production procedure.mp4 (Video illustrating the elicited production procedure)
- Prereg Production and Binary Judgments.pdf (Preregistration of the methods used)

Data are available under the terms of the Creative Commons Zero "No rights reserved" data waiver (CC0 1.0 Public domain dedication).

## Footnotes

^1^ The periphrastic causative form is termed the more-transparent form because it includes an overt causative marker ( *make*). More- and less-transparent causative forms for Hebrew, Hindi, Japanese and K’iche’ are set out in the Methods section.
