# Testing a computational model of causative overgeneralizations: Child judgment and production data from English, Hebrew, Hindi, Japanese and K’iche’

**DOI:** 10.12688/openreseurope.13008.2

**Published:** 2022-01-12

**Authors:** Ben Ambridge, Laura Doherty, Ramya Maitreyee, Tomoko Tatsumi, Shira Zicherman, Pedro Mateo Pedro, Ayuno Kawakami, Amy Bidgood, Clifton Pye, Bhuvana Narasimhan, Inbal Arnon, Dani Bekman, Amir Efrati, Sindy Fabiola Can Pixabaj, Mario Marroquín Pelíz, Margarita Julajuj Mendoza, Soumitra Samanta, Seth Campbell, Stewart McCauley, Ruth Berman, Dipti Misra Sharma, Rukmini Bhaya Nair, Kumiko Fukumura

**Affiliations:** 1 University of Liverpool, Liverpool, UK; 2 ESRC International Centre for Language and Communicative Development (LuCiD), Liverpool, UK; 3 Kobe University, Kobe, Japan; 4 Hebrew University of Jerusalem, Jerusalem, Israel; 5 Universidad del Valle de Guatemala, Guatemala City, Guatemala; 6 University of Salford, Salford, UK; 7 University of Kansas, Lawrence, Kansas, USA; 8 University of Colorado, Boulder, Boulder, Colorado, USA; 9 University of Calgary, Calgary, Canada; 10 University of Iowa, Iowa City, Iowa, USA; 11 Tel Aviv University, Tel Aviv, Israel; 12 Indian Institute of Information Technology, Hyderabad, India; 13 Indian Institute of Technology, Delhi, India; 14 University of Stirling, Stirling, UK

**Keywords:** child language acquisition, verb semantics, causative, English, Japanese, Hindi, Hebrew, K’iche', discriminative learning

## Abstract

How do language learners avoid the production of verb argument structure overgeneralization errors ( *The clown laughed the man c.f. *The clown made the man laugh*), while retaining the ability to apply such generalizations productively when appropriate? This question has long been seen as one that is both particularly central to acquisition research and particularly challenging. Focussing on causative overgeneralization errors of this type, a previous study reported a computational model that learns, on the basis of corpus data and human-derived verb-semantic-feature ratings, to predict adults’ by-verb preferences for less- versus more-transparent causative forms (e.g., * *The clown laughed the man *vs *The clown made the man laugh*) across English, Hebrew, Hindi, Japanese and K’iche Mayan. Here, we tested the ability of this model (and an expanded version with multiple hidden layers) to explain binary grammaticality judgment data from children aged 4;0-5;0, and elicited-production data from children aged 4;0-5;0 and 5;6-6;6 ( *N*=48 per language). In general, the model successfully simulated both children’s judgment and production data, with correlations of *r*=0.5-0.6 and *r*=0.75-0.85, respectively, and also generalized to unseen verbs. Importantly, learners of all five languages showed some evidence of making the types of overgeneralization errors – in both judgments and production – previously observed in naturalistic studies of English (e.g., *I’m dancing it). Together with previous findings, the present study demonstrates that a simple learning model can explain (a) adults’ continuous judgment data, (b) children’s binary judgment data and (c) children’s production data (with no training of these datasets), and therefore constitutes a plausible mechanistic account of the acquisition of verbs’ argument structure restrictions.

## Plain language summary

When learning their native language, children often produce errors in which they use verbs in "ungrammatical" sentence types (e.g., “*The clown laughed the man”, whereas an adult would say “The clown made the man laugh”). Although these examples are from English, similar errors are observed in many other languages, including Hebrew, Hindi, Japanese and K’iche Mayan. A previous study reported a computer model which, when trained on an approximation of real language input, simulated the relative grammatical acceptability of these errors with different verbs as judged by child and adult raters. The aim of this study was to investigate whether the same model (and a new, slightly more sophisticated version) could explain (a) binary judgments from younger children (4–5 year-olds, who were simply asked "Is this sentence acceptable" rather than "How acceptable is this sentence?" and (b) the rates at which children learning these five languages actually produce such errors for different verbs (e.g., *Someone laughed/danced/sang the boy). In general, the model performed very well on both tasks for all languages except K’iche’.

## Introduction

The question of how language learners come to avoid verb argument structure overgeneralization errors such as *The clown laughed the man – in some cases after a protracted period of producing them – has been described as a “learnability paradox” ( Pinker, 1989: 415); “one of the most…difficult challenges for all students of language acquisition” ( Bowerman, 1988: 73). The problem is this: On the one hand, children need to be able to use verbs in argument structure constructions in which they have not witnessed them; this type of productivity is the hallmark of human language. On the other hand, children need to be able to constrain this generalization process in order to avoid producing ungrammatical utterances such as *The clown laughed the man. These types of errors, in which English-speaking children incorrectly mark causation using the transitive causative for verbs that prefer the periphrastic causative (e.g., *The clown made the man laugh*) are the focus of the present study; along with equivalent errors in Hebrew, Hindi, Japanese and K’iche Mayan. Further naturalistically obtained examples of this error are summarized in Table 1 below (from the diary study of Ambridge & Ambridge, 2020). Similar errors have been observed in naturalistic data for Japanese ( Nakaishi, 2016; see also the experimental study of Fukuda & Fukuda, 2001), though they have not, to our knowledge, been investigated for any of the other languages included here.

**Table 1.** Transitive causative overgeneralization errors produced by an English-speaking child (reproduced under a CC BY 4.0 license from Ambridge, 2019; also reproduced in Ambridge & Ambridge, 2020).

Age	Error
2;3	Can you reach me? (Already being held, wants lifting up higher to touch sparkly part of a sign)
2;4	Can you jump me off? (wants help jumping down off the bed)
2;4	Did you drop the letters? (="Did you make the letters drop?” Foam letters stuck to the bathroom wall have fallen into the bath)
2;6	(Dad: why are you running?) It's practising me to run like that
2;6	jump me!
2;6	Don't swim me
2;7	Run me down, jump me down (wants to run down slide)
2;7	Jump me
2;7	Drink me. drink me, Dad! (Can't reach juice in bottom of cup and wants it tipped right back)
2;7	I'm just dancing it (shaking the bent-double flap of the elephant's door in Dear Zoo, to make it dance)
2;7	I can dance it (book)
2;7	I'm dancing it
2;7	This is the boat - swim it!
2;7	Swim that aeroplane (submarine)
2;7	Stay your leg up there (holding dad's leg)
2;7	Stop jumping them (Dad is tapping rabbits in Peter Rabbit game to make them jump)
2;7	drink me a bit (wants straw held up to her mouth to drink squash in bed)
2;10	The sheet's slipping me
2;11	Jump me, Dad! x5
2;11	I jumped my legs. I hopped my legs
3;2	I stand on your feet and you walk me
3;2	(Mum: what happens to the rubbish when it goes outside?). It gets died.
3;5	(Dad, playing with Shopkins: Now what are we doing?) Chloe: Going them in. (What?) Into the bathroom
3;6	I'm try to duck her under (pushing Aurora doll under the seat belt of Barbie car)
3;6	Pens are difficult to come off the paper
3;7	Reach me up there (wants to see toys on top shelf)
3;7	It will get died [die/get killed]
3;7	That nearly feeled me like I'm nearly falling off
3;8	I'm going it faster (exercise bike at airport)
3;8	Eat it in my mouth (pez sweet that has fallen onto floor - wants Dad to pick it up and post it into her mouth)
3;8	Disappear them and disappear them (scooping up bubbles in the bath)
3;9	Your turn to dance me, Dad (i.e., swing her around by the arms)
3;10	Those guys died Maleficent (watching Sleeping Beauty)
3;10	We died (dissolved) Mummy's special soap didn't we, Dad?
3;11	Jump me up there (wants putting onto the toilet seat)
3;11	I wanna jump her in (Ariel doll into bath)
3;11	It will die you; it will make you killed
4;0	Mermaids have got special powers; they can die baddies
4;7	Jump me x 2

 This problem has attracted a great deal of research attention ( Alishahi & Stevenson, 2008; Ambridge *et al.*, 2008; Ambridge *et al.*, 2009; Brooks & Zizak, 2002; Brooks *et al.*, 1999; Gropen *et al.*, 1991; Li & MacWhinney, 1996; Perfors *et al.*, 2010; Stefanowitsch, 2008; Theakston, 2004; Wonnacott *et al.*, 2008); Ambridge, 2013; Ambridge & Ambridge, 2020; Ambridge & Blything, 2016; Ambridge & Brandt, 2013; Ambridge *et al.*, 2011; Ambridge *et al.*, 2012a; Ambridge *et al.*, 2012b; Ambridge *et al.*, 2013; Ambridge *et al.*, 2014; Ambridge *et al.*, 2015; Ambridge *et al.*, 2018; Barak *et al.*, 2016; Bidgood *et al.*, 2014; Boyd & Goldberg, 2011; Blything *et al.*, 2014; Goldberg, 2011; Harmon & Kapatsinski, 2017; Hsu & Chater, 2010; Irani, 2009; Perek & Goldberg, 2017; Robenalt & Goldberg, 2015; Robenalt & Goldberg, 2016; Twomey *et al.*, 2014; Twomey *et al.*, 2016), including two book-length treatments ( Goldberg, 2019; Pinker, 1989). However, until a single recent study, research on the retreat from overgeneralization had been conducted exclusively on English (and mainly on dative and locative constructions).

 This recent study ( Ambridge *et al.*, 2020), sought to explain how speakers learn to avoid not only causative errors in English, (e.g., *The clown laughed the man), but also equivalent errors in Hebrew, Hindi, Japanese and K’iche’ Mayan. It also adopted a novel theoretical approach: Previous studies had attempted to explain this phenomenon in terms of three – to some extent – competing theories: preemption, conservatism *via* entrenchment (both statistical-learning theories) and verb semantics (from Ambridge *et al*., 2020: 2–4):

- Under the preemption hypothesis ( Goldberg, 1995), the use of a particular verb in a particular target structure (e.g., laugh in the less-transparent structure, as in *Someone laughed the boy) is deemed increasingly ungrammatical on the basis of occurrences of this verb in a nearly synonymous competing structure (e.g., the more-transparent structure, as in X made Y laugh). This account predicts a negative correlation between the acceptability of a particular error (e.g, *The clown laughed the man) and the corpus frequency of the relevant verb root in a competing structure (e.g., *X made Y laugh*); a prediction supported, for English, by the corpus and judgment studies of Goldberg (2011) and Robenalt & Goldberg (2015); Robenalt & Goldberg (2016).
- Under the [conservatism *via*] entrenchment hypothesis ( Braine & Brooks, 1995), repeated occurrences of a particular verb root (e.g., laugh) contribute to an ever-strengthening probabilistic inference that it cannot be used grammatically in structures in which it has not yet appeared (e.g., *The clown laughed the man; the transitive-causative); a kind of rational Bayesian inference from absence (e.g., Hsu *et al.*, 2017). Intuitively, one way to interpret entrenchment is the inference that “given how often I’ve heard this verb root in general, if it were permitted in this structure, I would have heard it by now”. This account predicts a negative correlation between the acceptability of a particular error (e.g, *The clown laughed the man) and the overall corpus frequency of the relevant verb root, regardless of the structure in which it occurs; a prediction supported, for English, by the corpus-judgment study of Stefanowitsch (2008).
- The ***verb-semantics*** hypothesis ( Pinker, 1989; Shibatani & Pardeshi, 2002) starts from the assumption that the distinction between verbs that allow (or prefer) *less-* versus *more-transparent* causation (e.g., *break, move, roll, spin* versus *laugh, cry, fall, disappear*) is not arbitrary, but reflects the semantics of those verbs. The most straightforward characterization is that actions of the latter type (e.g., *laugh*) “have internal causes that would make any external prodding indirect” ( Pinker, 1989: 302), meaning that causation can be expressed only via a dedicated, transparent causative marker ( *make, -(s)ase, -aa, hiCCiC* or *–isa-j*); and even this causation is often rather indirect (e.g., Bowerman, 1988:91 points out that *John made the baby stand up* could imply simply giving an order). In contrast, verbs of the former type (e.g., *break*) are more amenable to external causation, particularly direct, physical causation ( Smith, 1970). Thus, for these verbs, causation does not require a dedicated surface marker (hence “less-transparent”). Because causation is inherent in the meaning of the verb itself (e.g., *break* already means ‘cause to become broken’), this meaning comes “for free” in a basic transitive sentence.

While each of these mechanisms enjoys considerable empirical support independently (see the reference list in the previous paragraph), Ambridge *et al.* (2020) sought to unify these theories by building a computational model that yields all three effects in a single learning mechanism.

The model developed by Ambridge *et al.* (2020) – a simple two-layer connectionist network – is trained on input-output pairs consisting of a verb (e.g., *break*) and a causative type (e.g., for English, either the transitive causative or the *make* periphrastic causative respectively), in proportion to the frequency of each in a representative input corpus (e.g., for English, the frequency of *[CAUSER] [BREAK] [CAUSEE] vs [CAUSER] [MAKE] [CAUSEE] BREAK*). Other corpus utterances containing the relevant verb (e.g., intransitive *[ACTOR] [BREAK]*) are mapped to a catch-all “Other” output node. Crucially, the input to the model consists not only of an orthogonal (one-hot) “lexical” verb representation that uniquely identifies each verb stem, but also four “semantics” units. The (continuous) activation level of these units is set on the basis of human ratings of four semantic properties thought to be relevant to languages’ preferences for less-transparent (e.g., *X broke Y*) versus more-transparent ( *X made Y break*) causative forms respectively (e.g., Shibatani & Pardeshi, 2002) ^1^. These semantic ratings were obtained by showing native adult speakers of each language an animation depicting the action described by each verb (though they were not given the verb itself) and asking them to rate:

**Event-merge:** The extent to which the causing and caused event are two separate events or merge into a single event that happens at a single time and a single point in space

**Autonomy** of the causee

**Requires:** Whether the caused event requires a causer

**Directive:** Whether causation is directive (e.g., giving an order) or physical

It is important to note that the model was not given any information regarding human judgments of the grammatical acceptability of the more- and less-transparent causative forms of each verb (which would make its learning task trivially simple, and akin to a conventional statistically regression model conducted on participants’ grammaticality judgments). Rather, the model was trained to “predict” the forms that occurred in a suitable corpus for each language. For example, if the English corpus contained the utterance *You broke it*, the target output was [1,0,0] for the less-transparent, more-transparent and “other” output nodes respectively; the corresponding input node values were [0,0,1,0,0,0….] on the lexical nodes (indicating “break”), [1] on the causative node (indicating causative), and [0.90, 0.90, 0.87, 0.85] for the semantic units corresponding to event-merge, autonomy, requires and directive.

Having learned the input-output mappings for the corpus, the model was – at test – presented with each verb ( *N*=60) and interrogated for its prediction of a causative form (e.g., for English, transitive causative *vs* periphrastic causative with *make*; *Someone laughed the boy vs *Someone made the boy laugh*). The resulting activation level of the corresponding output units was taken as the model’s “grammaticality judgment” for that form. These judgments were then correlated against those obtained from native speakers of each language ( *N*=48 at each of ages 5–6, 9–10 and adults). Note, again, that the model never saw these judgments, having been trained only a suitable input corpus for that language.

Because we adopt the same model in the present article, it is important to fully set out here the details of its architecture. In fact, although Ambridge *et al.* (2020) described their model as a discriminative-learning model that used the Widrow-Hoff learning rule (p.17), this was an error. While the model resembled discriminative learning models in its absence of any hidden layers, it actually used the Broyden-Fletcher-Goldfarb-Shano (BFGS) learning rule. Because the model’s task was to choose, on each learning trial, between a set of mutually exclusive output nodes, the softmax activation function was used. The model did not have a learning-rate parameter; the only free parameters were range, which specifies the range of the initial random weights, and weight decay, both set to 0.5 for all simulations. These settings were not varied systematically, although informal experimentation revealed (a) that (near-) zero settings for decay harmed the model’s performance (presumably by causing the model to over-fit the training data) but (b) changes to the initial random weights made no difference (presumably because the results are always averaged across 48 runs with different initial weights).

In general, the model reported in Ambridge *et al.* (2020) achieved correlations of around *r*=0.75 with human judgments, showing only a small decrement in performance (i.e., slightly lower correlations) when tested on verbs that had been withheld during training, using split-half validation. (Though note that this “split half validation” did NOT consist of training the model on half of the participants’ grammaticality judgments and having it predict the held-out half. Rather, it consisted of withholding half of the verbs from the corpus-derived training set, before interrogating it for its predictions for the held-out verbs). This finding demonstrates that the model, like human learners, eventually reaches a point at which it is able to produce the appropriate causative form for verbs that it is encountering for the first time, on the basis of their semantics. Importantly, prior to this point, the model displays an “overgeneralization” stage analogous to that shown by children (at least for English). For example, when presented with *laugh*, the English model initially produces the transitive causative construction (e.g., *Someone laughed the boy) with considerably higher probability than the periphrastic causative (e.g., *Someone made the boy laugh*). After around 12 epochs of training (each consisting of 10,000 corpus utterances) the probabilities begin to flip, and the model asymptotes at predictions of around 0.7 *vs* 0.3 for the periphrastic- versus transitive-causative respectively (“Other” uses are around zero, since the model is interrogated for a causative form).

While these findings constitute support for the model developed by Ambridge *et al.* (2020), this support is currently limited in three ways.

First, the model was assessed only on its ability to predict grammaticality judgment data obtained from older children (5–6 and 9–10 years) and adults. However, the available English data (e.g., Ambridge & Ambridge, 2020; Bowerman, 1988; Pinker, 1989) suggest that the majority of such overgeneralization errors are produced before this age. Indeed, for languages other than English, there is no more than anecdotal evidence that children produce such errors at all (either at age 5–6 or younger). Thus, while the model does well at explaining which forms are ungrammatical for adults and older children, it remains to be seen whether it really explains the *retreat* from overgeneralization, which is well underway – and perhaps largely completed – by age 5.

Second, and relatedly, although the model of Ambridge *et al*. (2020) simulated the retreat from overgeneralization in a macro sense (e.g., initially predicting *Someone laughed the boy over *Someone made the boy laugh*), at no stage does it predict children’s verb-by-verb acceptability judgments better than adults’. Again, this calls into question the extent to which it is truly simulating the *retreat* from overgeneralization.

Third, and again relatedly, Ambridge *et al*. (2020) used only a single model architecture with a single set of parameter values. Thus, it remains to be seen whether other architectures and settings – including those designed to more closely reflect children’s memory and processing limitations – might better simulate the child data; and perhaps even the developmental changes observed between childhood and adulthood.

The present study therefore has three aims. The first is to test the ability of the computational model developed by Ambridge *et al.* (2020) to explain (Study 1) grammaticality judgment data from younger children than those tested previously; children aged 4;0-5;0, which necessitates the use of a binary judgment task (rather than the Likert-scale task used with children aged 5;6-6;6) and (Study 2) children’s production data, including possible overgeneralization errors, at ages 4;0-5;0 and 5;6-6;6. The second aim (Studies 1–2) is to investigate, with these data from younger children in hand, whether the model of Ambridge *et al.* (2020) can explain development, i.e., the retreat from overgeneralization from childhood to adulthood. The third aim (Study 3) is to investigate whether other model architectures, including more advanced multiple-layer models, explain both these cross-sectional and developmental patterns of judgment and production data.

### Ethics statement

For both Study 1 and Study 2, ethics approval was obtained from the University of Liverpool (approval number RETH001041), as the institution with overall responsibility for the project, and from local ethics committees at the Hebrew University of Jerusalem (22032020), the International Institute of Information Technology Hyderabad (IIITH/IEC/2016/1), and the Universidad del Valle de Guatemala (¿Cómo los niños adquieran la estructura de oraciones en K’iche’?). Japanese universities do not routinely provide ethics review for psychological or linguistic research. In lieu, we therefore obtained a review from Shunzo Majima, Associate Professor at the Center for Applied Ethics and Philosophy, Hokkaido University. Parents/caregivers gave informed written consent on behalf of their children, who provided verbal assent. Written consent included both participation in the study and inclusion of the data in an anonymized publicly-available dataset.

## Study 1: Binary grammaticality judgments (4;0-5;0)

### Methods

**Preregistration.** The sample size, materials, data collection methods and analysis plan were pre-registered at https://osf.io/qhnjk, on 15 ^th^ May 2018, before data collection began. We deviate here from our planned data analysis plan, which was designed to constitute separate tests of the preemption, entrenchment and verb semantics hypothesis. In our view, such an analysis is no longer meaningful, given that (a) Ambridge *et al.* (2020) reported extremely high levels of collinearity between the preemption and entrenchment predictors ( *r*=0.75-0.96 for difference scores, depending on the language) and (b) our goal is now to test the computational model of Ambridge *et al.* (2020) which collapses the distinction between preemption, entrenchment and verb semantics into a single learning mechanism. That said, the analyses we report are “pre-registered” in the sense that they correspond directly to those reported in the computational modeling section of Ambridge *et al.* (2020); the only difference being that the by-verb predictor variable averages across participants’ binary grammaticality judgments (Study 1) or binary production data (Study 2), rather than continuous grammaticality judgments. As such, other than the decision to switch to these analyses in the first place, we have retained no researcher degrees of freedom ( Wicherts *et al.*, 2016). To be explicit, we are not switching our analysis plan because the original plan failed to yield a particular pattern of results: We have not conducted the analyses specified in the original analysis plan.

**Computational model.** The model architecture was identical to that reported in Ambridge *et al.* (2020; see the present Introduction for a brief outline), though new model runs were conducted (48 runs for each of 50 epochs, for each language, as in Ambridge *et al.*, 2020).

**Participants.** Our preregistered analysis plan said that we would recruit 48 children aged 4;0-5;0 for each language: English, Hebrew, Hindi, Japanese and K’iche’. We achieved this target for every language except K’iche’ ( *N*=32), for which testing had to be terminated early due to the coronavirus pandemic. All children were native learners of the relevant language, although many would have had some limited exposure to English (particularly the Hindi-speakers) and – for K’iche’ speakers – Spanish. The target sample of *N*=48 per language was specified in the initial grant application, but was arrived at informally on the basis of the first author’s previous work, not a power calculation. Children were recruited via schools/nurseries in the UK, Israel, India, Japan and Guatemala. Parents/caregivers were sent an invitation letter and consent form. Parents/caregivers were asked not to volunteer if their children had any known or suspected language difficulties, or were not native learners of the relevant language.

**Stimuli and materials.** The sentences used in the grammaticality judgment task, along with the animations used to illustrate their intended meanings, were identical to those used in Ambridge *et al.* (2020), to which the reader is referred for a detailed description. The full set of sentences for each language can be viewed at https://osf.io/84qjh/, and the accompanying animations at https://osf.io/x6hyw/. Each sentence included either the more- or less-transparent causative form of one of the standardized set of 60 verbs (i.e., translational equivalents across languages) used in Ambridge *et al.* (2020), always with “Someone” as the causal subject (e.g., Someone made the boy laugh; *Someone laughed the boy). Further examples, for the verb laugh, are shown for each language in Table 2. The accompanying animations depicted the caused event, but not the causer, who was obscured using stage curtains. For example, for the sentences shown in Table 2, the animation depicted a boy alone on a stage; the curtains then closed and reopened to show the boy laughing.

**Table 2.** Less-transparent and more-transparent causative sentences for the verb LAUGH for each language. For the more-transparent causative, the overt causative marker is shown in bold type.

	Less-transparent causative	More-transparent causative
English	*Someone laughed the boy	Someone **made** the boy laugh
Hebrew	*Mishehu caxak et ha-yeled	Mishehu **hi**cx **i**k et ha-yeled
Hindi	*kisii=ne laRke=ko hããs-aa	kisii=ne laRke=ko hãs- **aa**-yaa
Japanese	Dareka ga otokonoko o warawasu	Dareka ga otokonoko o waraw **ase**ru
K’iche’	x-0-u-tze'-j le ak'al le achi	x-0-u-tze'n- **isa**-j le ak'al le achi

**Procedure.** Data were collected between January 2018 and March 2020 in schools and nurseries in the UK, Israel, India, Japan and Guatemala. Because the full set of 120 judgments would have been too onerous for young children, each child completed 60 judgments – more- and less-transparent forms for each of 30 verbs – according to one of four counterbalance lists (which can be viewed at https://osf.io/hsm3b/). These 60 judgments were split into two sessions of 30, given either on different days or on the same day with a break in between. For each child, 16 (or 14) verbs were rated in both more- and less-transparent form in the same session; the remaining 14 (or 16) verbs were rated in more-transparent form in one session and less-transparent form in the other session. A video of the procedure can be found at https://osf.io/fqyps/.

 The procedure, which involved the child placing a small animal toy on a green tick or a red cross, indicating “grammatical” and “ungrammatical”, respectively ( Theakston, 2004), is best summarized by the instructions that were given to children (in translation):

We are going to play a game. This dog is trying to learn to speak English (/Hindi etc.). So, we’re going to watch some short videos, and he’s going to tell us what’s happening. We have to help him by telling him when he says it right, and when he gets it wrong and says it a bit funny. In the game, we will watch a cartoon and the dog will tell us what happens. We have to listen to the dog and then if he says something that sounds okay we put the toy on the tick **[demonstrates to child]** and if he says something that sounds a bit silly then we put the toy on the cross **[demonstrates to child, then completes practice trials 1 (tick) and 2 (cross). Child completes practice trials 3 (tick) and 4 (cross)]**. We’re going to play the game again, but this time the cartoons are going to look a bit different **[shows still of animation]**. They’re going to have either this little boy or something else on this stage. These big red curtains are going to close, and you have to imagine that there is someone is behind the curtains and that person is going to do something to make something change, so that when the curtains reopen you can see how its changed. So, let’s see how this one changes. **[plays example animation:** ***dress*]**. So as you can see, in this cartoon the person behind the curtains has done something to help or make the boy get dressed. So, when we play the game again all the sentences our dog is going to say are going to start with someone and that is who the someone is, the person behind the curtains. But we’re going to play the game the same where we watch the cartoon, the dog says the sentence and we listen and then we put the toy on the tick if it sounds okay or the cross if it sounds a bit silly. You’ve also got this grid. To win the game you need to fill all these boxes with a sticker. You’ll get a sticker every time you hear this sound **[plays dog barking sound effect]**. Once there is a sticker in all of the boxes you win.

The practice trials referred to are (1) *The cat drank the milk*, (2) *The dog the ball played with, (3) *The frog caught the fly*, (4) *His teeth the man brushed (or sentences with equivalent word order errors in the other languages). The example animation with *dress* was created solely for use as an example, and did not appear in the main stimulus set (or in Study 2). The barking sound effect was automatically triggered by the software displaying the animations (PsychoPy 2; Peirce *et al.*, 2019), such that the child completed her grid and won the game on the final trial of each day. The experimenter also used this software to record the child’s response for each trial (grammatical, ungrammatical, equivocal/refused to answer). Responses of the latter type, which were very rare, were discarded for all statistical analyses.

**Analysis.** All analyses were conducted in *R* (version 3.6.3; R Core Team, 2020). All computational models were built using the *nnet* package (version 7.3-14; Venables & Ripley, 2002). Correlations were conducted using the *cor* function of base R. All plots were made using *ggplot2* (version 2.2.1; Wickham, 2016).

### Results: Binary grammaticality judgments (4;0-5;0)

Before proceeding to test the computational model, it is instructive to compare children’s binary judgment data against the gold-standard adult continuous judgment data reported by Ambridge *et al.* (2020) in order to determine (a) whether children aged 4;0-5;0 give meaningful judgments and (b) whether they make judgments that correspond to overgeneralization errors, rating as “acceptable” sentences that receive low acceptability ratings from adults.

**Figure 1.**
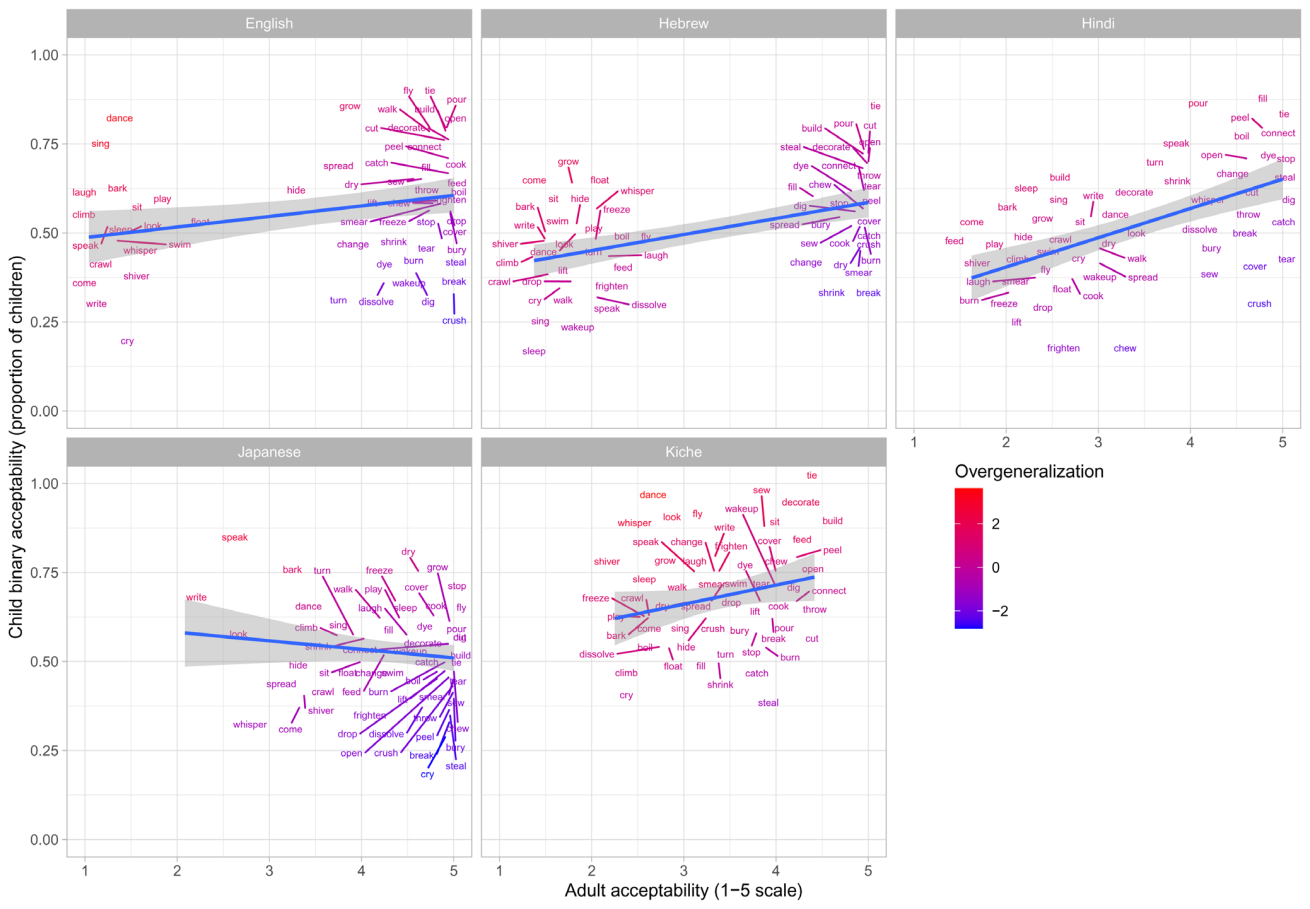
Child binary judgments (present study) versus adult continuous judgments for less-transparent forms.

**Figure 2.**
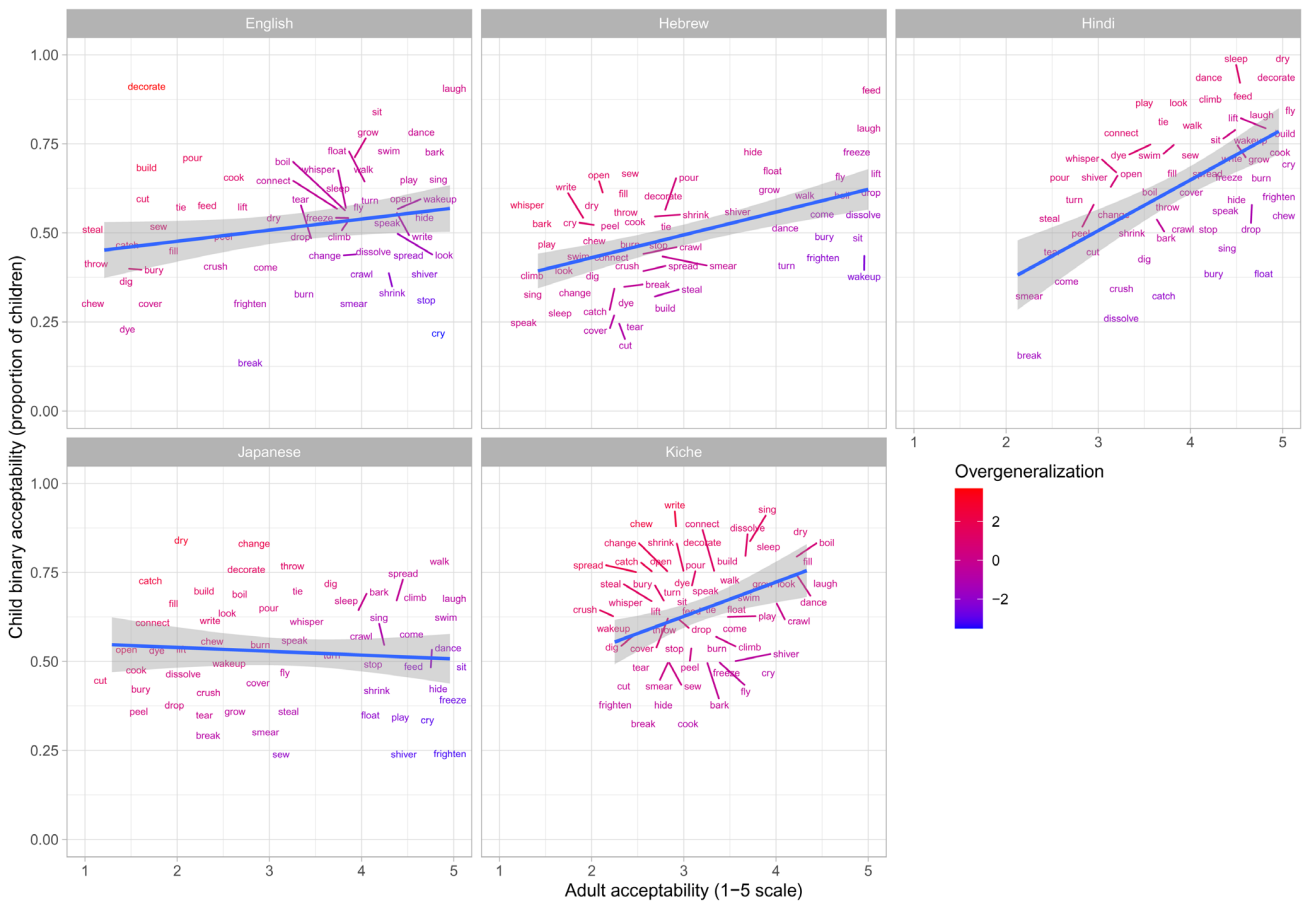
Child binary judgments (present study) versus adult continuous judgments for more-transparent forms.

 These data are plotted in Figure 1– Figure 2 for less-transparent forms (e.g., *Someone laughed the boy) and more-transparent forms (e.g., *Someone made the boy laugh*) respectively. The x-axis shows, for each verb form, the mean acceptability rating given by adults on the five-point scale. The y-axis shows, for each verb form, the proportion of children accepting that form (recall that each child makes only a single binary acceptability judgment for each form). Forms are colour coded to indicate child judgments that correspond to “overgeneralization errors” at the group level. This was done by converting by-verb mean adult acceptability judgments and by-verb child acceptability proportions into Z-scores, and subtracting the former from the latter. A large positive score (red) represents overgeneralization. For example, in Figure 1 (less-transparent forms), English *dance* and *sing* are red, since around 75% of children deemed *Someone danced the boy and *Someone sang the boy to be acceptable, despite the fact that adults assigned mean acceptability ratings close to the minimum possible (1/5) for both. A large negative score (blue) represents “undergeneralization” (i.e., children considering a form to be *less* acceptable than it is for adults). For example, in Figure 1 (less-transparent forms), English *break* and *crush* are blue, since only around 30–40% of children deemed *Someone broke the truck* and *Someone crushed the can to be acceptable* (close to 5/5 for adults).

**Figure 3.**
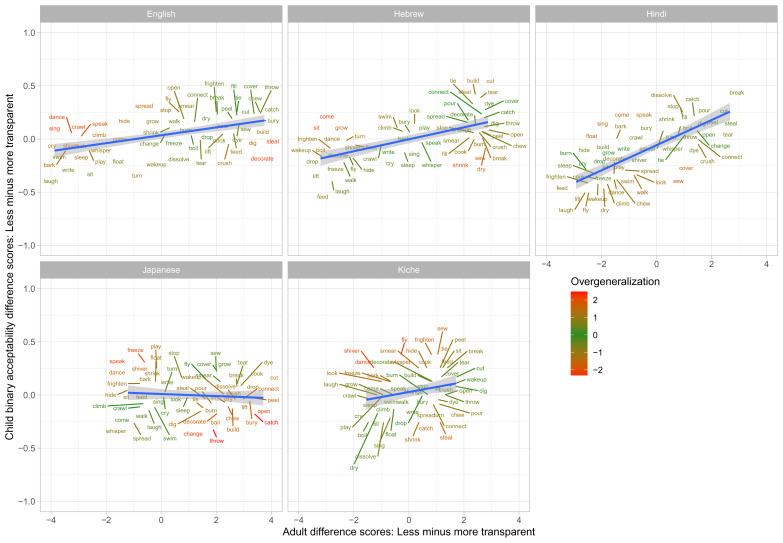
Child binary judgments (present study) versus adult continuous judgments for difference scores (less- minus more-transparent).

Figure 3 shows the corresponding data for difference scores (calculated as less- minus more- transparent). This figure is colour coded such that, for green verbs, children’s (binary) difference scores effectively match adults’ (continuous) judgment scores, while red verbs constitute child “overgeneralization” errors in either direction. For example, English *dance* is shown in red because children show very close to zero preference for *Someone made the boy dance* over * *Someone danced the boy*, while adults show a preference of around 3 points on the 5-point scale. Conversely, English *steal* is also shown in *red* because children show very close to zero preference for *Someone stole the jewellery* over * *Someone made the jewellery steal*, while adults show a difference of around 4 points on the 5-point scale. English *freeze* on the other hand is shown in green because children – just like adults – show essentially no preference for *Someone froze the water* over *Someone made the water freeze*; i.e., both children and adults deem both forms to be more-or-less equally acceptable.

Comparison of Figure 1– Figure 2 against Figure 3 suggests an intriguing and important pattern. At first glance – i.e., looking only at their raw judgments ( Figure 1– Figure 2) – children, for all five languages, seem to make a considerable number of “overgeneralization errors”; i.e., accepting forms that adults deem ungrammatical. When we look at difference scores ( Figure 3), however, quite a different pattern emerges: for all five languages, the vast majority of verbs are coloured green, showing that children’s judgments generally mirror those of adults. What is giving rise to this apparently paradoxical pattern? In fact, there is no paradox: the overall pattern can be explained by assuming that, as a group, (a) children’s underlying grammatical knowledge is essentially adultlike by this age, but (b) children are more tolerant than adults of forms that deviate from that underlying grammar. As an example, consider the English verb *laugh*. As a group, English-speaking 4–5-year olds know that * *Someone laughed the boy* is considerably less acceptable than *Someone made the boy laugh* (with a difference score of around -0.4; see Figure 3). Nevertheless, in absolute terms, English-speaking 4–5-year olds are relatively tolerant of * *Someone laughed the boy* with around 60% judging it as acceptable (see Figure 1; the difference sore of −0.4 arises because close to 100% of children accept *Someone made the boy laugh*; Figure 2).

In order to verify this pattern statistically, we ran a series of mixed-effects models using the lme4 package ( Bates *et al*., 2015) with the following (example) syntax:

English_Less=glmer(Rating ~ Adult_Less_Transparent + Valence +(1||verb) + (1+Adult_Less_Transparent+Valence||participant), data=subset(English, type=="Less_Transparent"), family="binomial"(link="logit"), control=glmerControl(optimizer="bobyqa",optCtrl=list(maxfun=2e5)))

In order to ensure a consistent model structure across languages and analyses (raw/difference scores), we did not construct the maximal converging model in each case, but instead adopted a near-maximal structure with random intercepts for verb and participant, and by-participant random slopes for adult-ratings and verb valance ratings. Verb valence ratings (from Warriner *et al*., 2013) were included as a control predictor, since the researchers who worked with the children expressed concern that children’s ratings seemed to be affected by the social desirability of the actions (particularly for crosslinguistically less-transparent-preferring verbs like *break, steal, crush, burn* etc.). All predictors were scaled and centered such that the intercept represents the adult acceptability rating for a (hypothetical) verb with the mean raw acceptability rating/difference score, and mean valence (i.e., neither particularly desirable or undesirable in terms of the action described). For the raw binary-acceptability models (corresponding to Figure 1– Figure 2), binomial models were used, as per the syntax above (which automatically generates *p* values via the *z* distribution). For difference-score models, where the possible responses for a given verb pair (less-/more-transparent form) are 1, 0 and -1, linear models were used, and *p* values calculated via the *t* distribution (lmerTest package; Kuznetsova *et al*., 2017).

The models are summarized in Table 3– Table 5. Focussing on difference scores ( Table 5), the adult continuous judgments are highly significantly predictive of children’s binary judgments (at *p*=0.001 or better) for English, Hebrew and Hindi; but not for Japanese and K’iche’, where children are heavily influenced by valance (also significant for Hindi): the less acceptable the action, the more children prefer the more transparent form; (e.g., *making something break*, which hints at unintentionality, is more acceptable than *breaking something*, which suggests a more intentional act). Similarly for raw ratings ( Table 3– Table 4), for English, Hebrew and Hindi the adult continuous judgments are significantly predictive of children’s binary judgments (at *p*=0.001 or better) for less-transparent, more-transparent or both forms. Notice however that, for raw ratings, the intercept is always positive – for four out of 10 models significantly so – indicating that, as suggested by Figure 1– Figure 2, children are more lenient in their acceptability judgments than are adults.

**Table 3.** Binary judgment task. Mixed effects models for less-transparent forms.

	*Est*	*SE*	*Z*	*p(z)*
**English**				
(Intercept)	0.31	0.12	2.66	**0.008**
Adult_Less_Transparent	0.35	0.11	3.19	**0.001**
Valence	0.38	0.10	3.68	**0.000**
**Hebrew**				
(Intercept)	0.03	0.19	0.15	0.883
Adult_Less_Transparent	0.46	0.16	2.91	**0.004**
Valence	0.09	0.10	0.88	0.379
**Hindi**				
(Intercept)	0.10	0.12	0.81	0.417
Adult_Less_Transparent	0.42	0.08	5.02	**0.000**
Valence	0.16	0.08	1.89	0.059
**Japanese**				
(Intercept)	0.16	0.15	1.07	0.284
Adult_Less_Transparent	-0.05	0.06	-0.81	0.421
Valence	0.21	0.06	3.24	**0.001**
**K'iche**				
(Intercept)	0.90	0.20	4.55	**0.000**
Adult_Less_Transparent	0.00	0.10	-0.01	0.992
Valence	0.26	0.08	3.18	**0.001**

**Table 4.** Binary judgment task. Mixed effects models for more-transparent forms.

	*Est*	*SE*	*Z*	*p(z)*
**English**				
(Intercept)	0.06	0.11	0.56	0.572
Adult_More_Transparent	0.09	0.10	0.97	0.334
Valence	0.43	0.08	5.06	**0.000**
**Hebrew**				
(Intercept)	-0.02	0.19	-0.08	0.934
Adult_More_Transparent	0.40	0.09	4.46	**0.000**
Valence	0.15	0.08	1.80	0.072
**Hindi**				
(Intercept)	0.70	0.14	4.86	**0.000**
Adult_More_Transparent	0.45	0.11	3.94	**0.000**
Valence	0.36	0.11	3.28	**0.001**
**Japanese**				
(Intercept)	0.13	0.16	0.82	0.414
Adult_More_Transparent	-0.06	0.08	-0.75	0.455
Valence	0.25	0.09	2.61	**0.009**
**K'iche**				
(Intercept)	0.96	0.25	3.86	**0.000**
Adult_More_Transparent	0.09	0.09	0.93	0.354
Valence	0.27	0.13	2.02	**0.043**

**Table 5.** Binary judgment task. Mixed effects models for difference scores (less minus more transparent).

	*Est*	*SE*	*df*	*t*	*p(z)*
(Intercept)	0.06	0.02	45.47	2.95	**0.005**	
Adult_Difference_Score	0.09	0.03	52.52	3.49	**0.001**	
Valence	-0.01	0.02	33.17	-0.57	0.572	
(Intercept)	0.51	0.04	52.58	14.24	**0.000**	
Adult_Difference_Score	0.06	0.02	33.20	3.79	**0.001**	
Valence	-0.01	0.02	43.56	-0.74	0.464	
(Intercept)	0.37	0.03	67.74	13.25	**0.000**	
Adult_Difference_Score	0.08	0.02	48.78	3.90	**0.000**	
Valence	-0.06	0.02	57.58	-3.08	**0.003**	
(Intercept)	0.48	0.03	58.16	15.66	**0.000**	
Adult_Difference_Score	0.00	0.02	38.37	-0.08	0.934	
Valence	-0.06	0.02	47.40	-2.92	**0.005**	
(Intercept)	0.33	0.04	32.44	8.39	**0.000**	
Adult_Difference_Score	0.00	0.02	28.67	-0.09	0.931	
Valence	-0.05	0.02	24.54	-2.24	**0.035**	

To sum up, then, at least for English, Hebrew and Hindi (for Japanese and K’iche’, children were overly affected by valence) 4–5-year-old children seem to have generally adultlike grammatical knowledge (i.e., children’s acceptability judgments are very well predicted by adults’) but also – sitting atop that knowledge – a tendency to over-accept forms that adults reject. Why?

One possibility is that children’s over-acceptance of ungrammatical forms (relative to adults) results from the use of a meta-linguistic task. For example, in a categorization task Kapatsinski, Olejarczuk and Redford (2017) found that 9–10-year-old children are more accepting than are adults of new exemplars that deviate from previous exemplars of the categories. Focussing specifically on the present task, children might be reluctant to “correct” or “hurt the feelings of” the talking dog who produced the forms. A second possibility is that. at least beyond age 4–5, children’s underlying grammatical knowledge (at least in this particular domain) is essentially adultlike, and the solution to the no-negative-evidence problem lies not with grammatical learning, but with increasing meta-linguistic and/or meta-cognitive abilities. These might take the form of, for example, an increasing willingness to judge others’ utterances as unacceptable, or improvements in executive function that allow children to inhibit their own tendency to overgeneralization (whether in judgments or production). Anecdotally at least, children do sometimes correct their errors spontaneously, particularly when adults repeat children’s errors back to them. The production and computational modeling studies reported later in this paper are key to teasing apart these possibilities.

**Figure 4.**
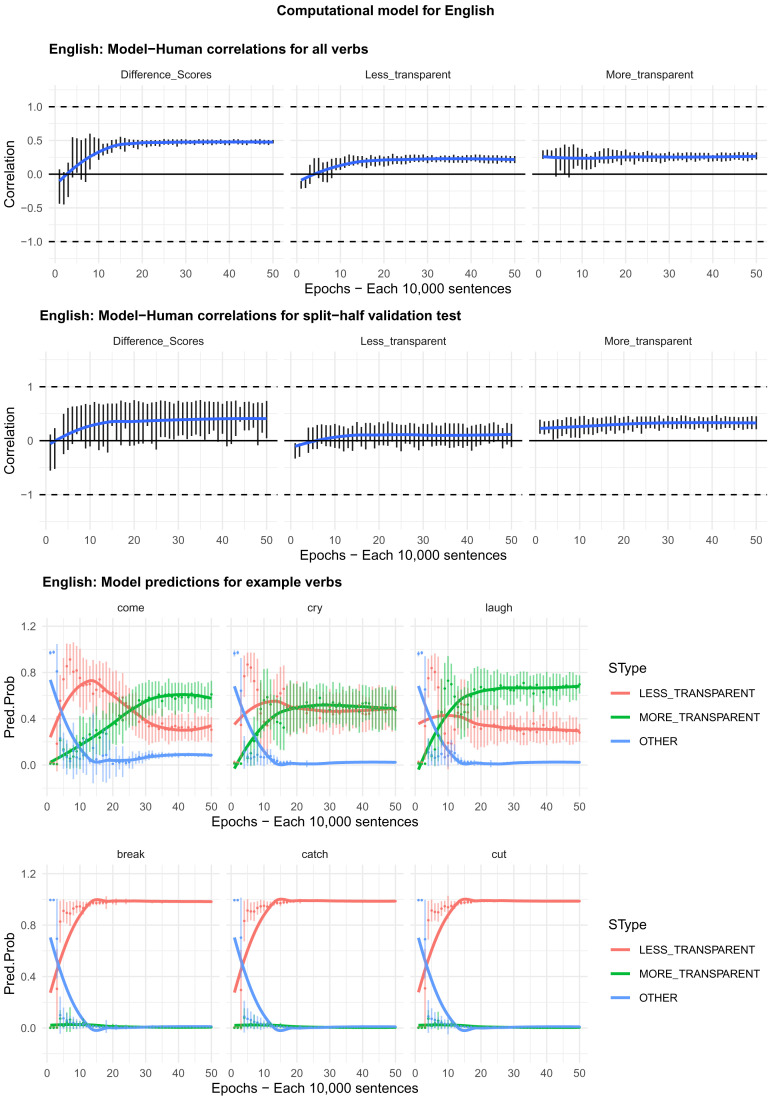
Model-child correlations for English binary judgment data.

Moving on to the tests of the computational model, Figure 4– Figure 8 plot – for English, Hebrew, Hindi, Japanese and K’iche’, respectively – model-child correlations for (a) the full set of 60 verbs, and (b) the split-half validation test (30 verbs, randomly selected for each run). Again, it is important to stress that the split-half validation test did NOT consist of training the model on half of the participants’ grammaticality judgments and having it predict the held-out half. Rather, it consisted of withholding half of the verbs from the corpus-derived training set, before interrogating it for its predictions for the held-out verbs. The figures also plot the developmental pattern shown by the model for a number of example verbs. For children’s judgments, the dependent measure is again the proportion of children judging the particular verb form (more-/less-transparent) to be acceptable on the binary judgment task (or a less-minus-more-transparent difference score). The predictor variable is the mean activation level of the corresponding unit of the model (or a difference score calculated in the same way).

**Figure 5.**
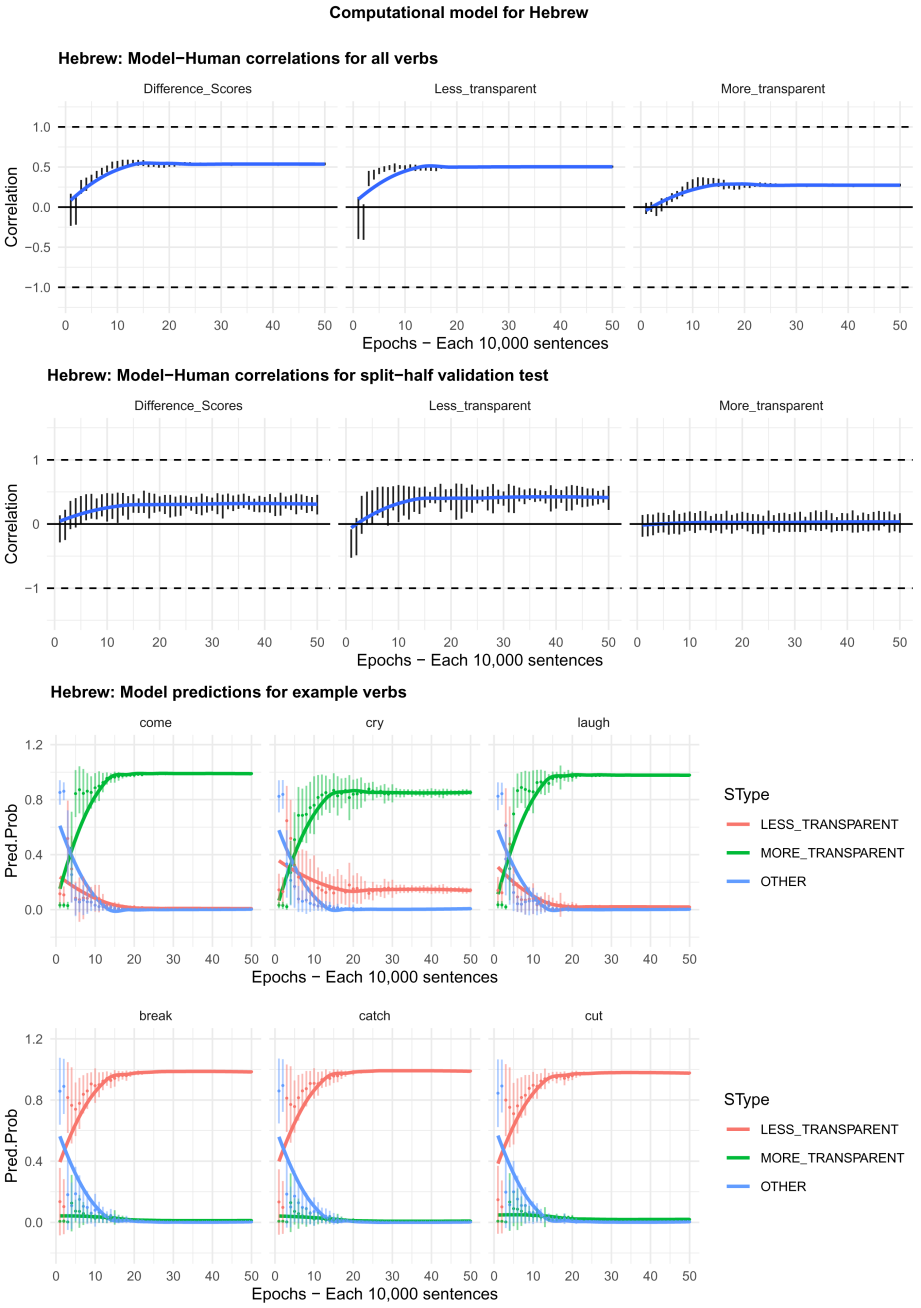
Model-child correlations for Hebrew binary judgment data.

**Figure 6.**
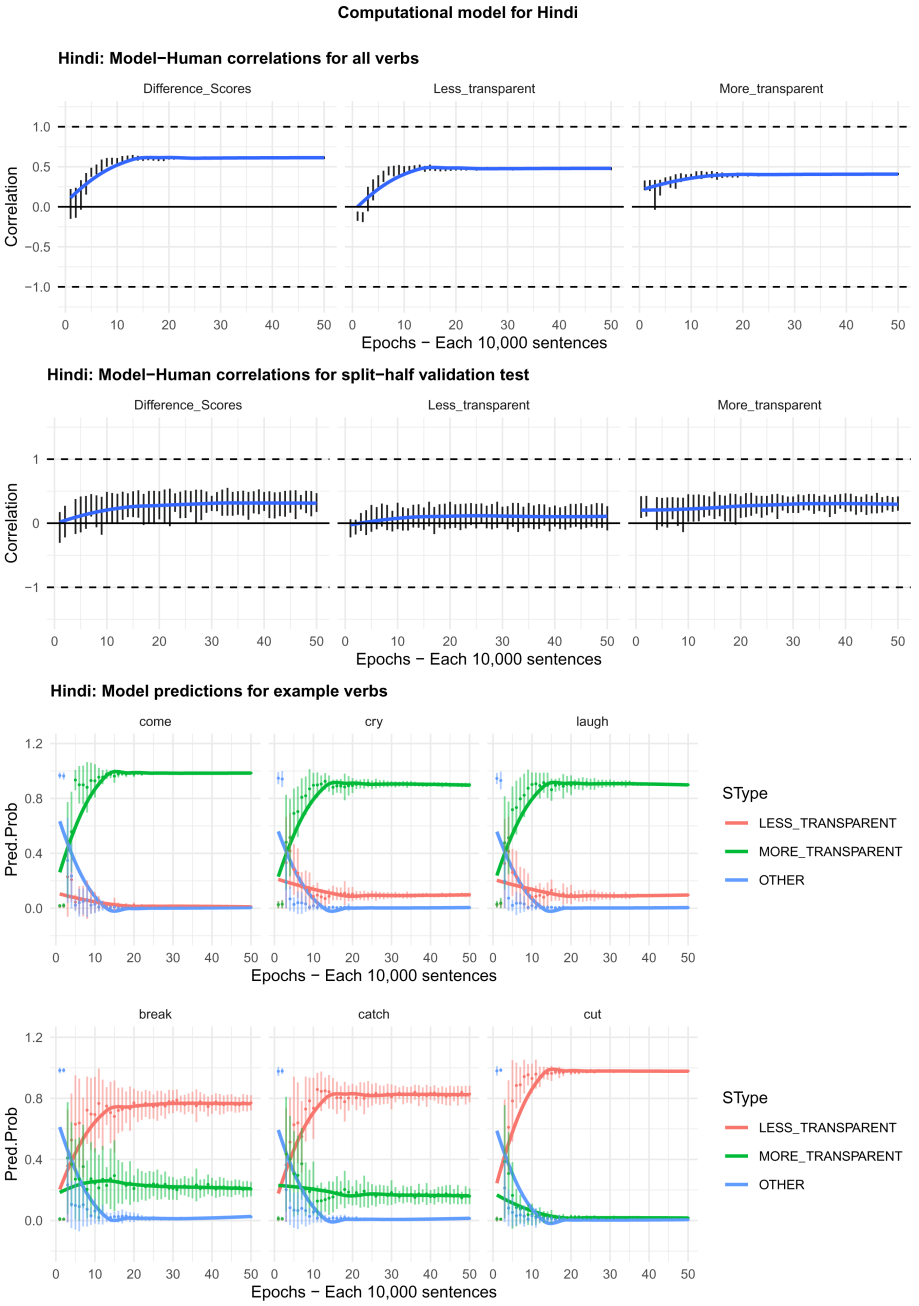
Model-child correlations for Hindi binary judgment data.

**Figure 7.**
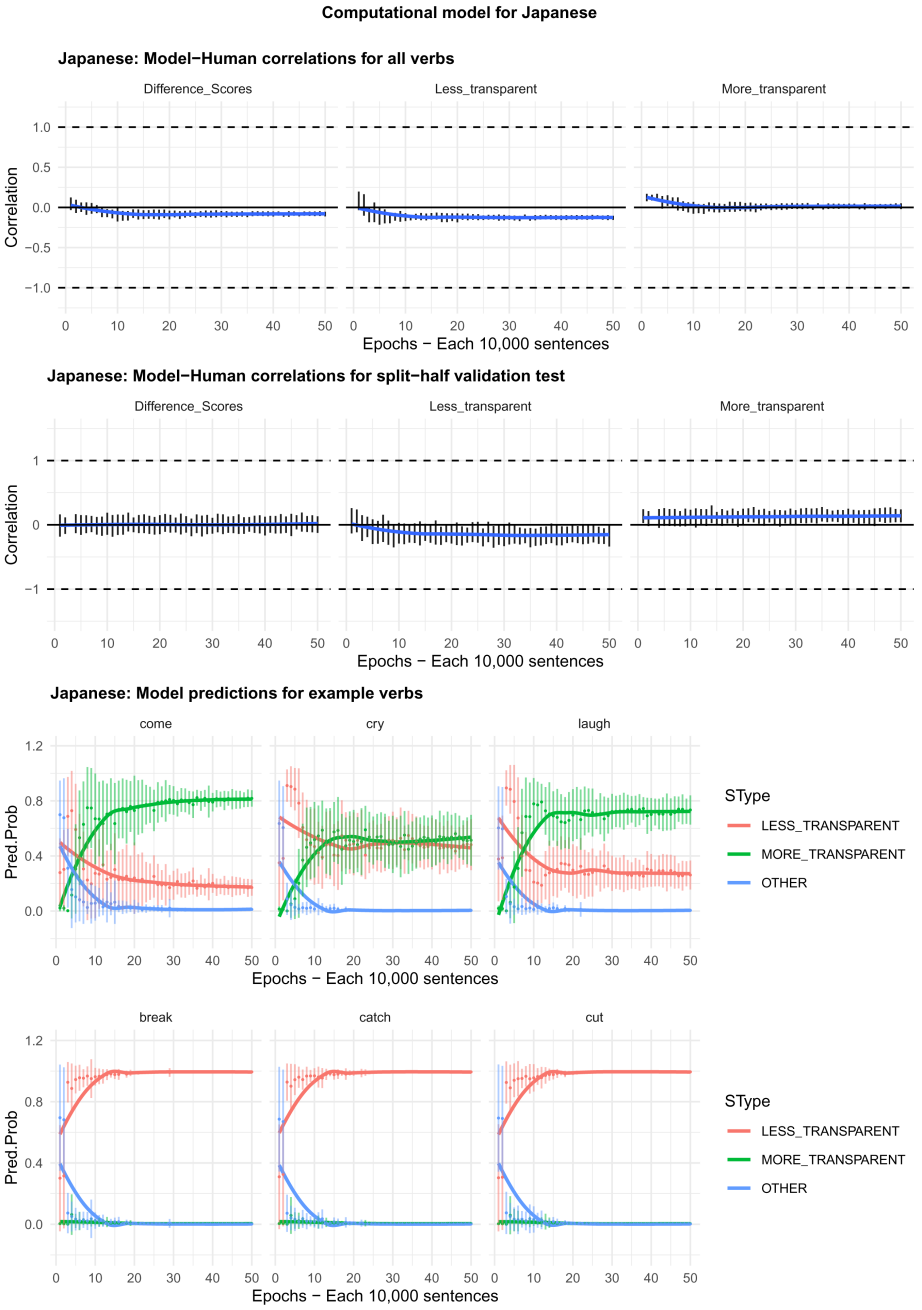
Model-child correlations for Japanese binary judgment data.

**Figure 8.**
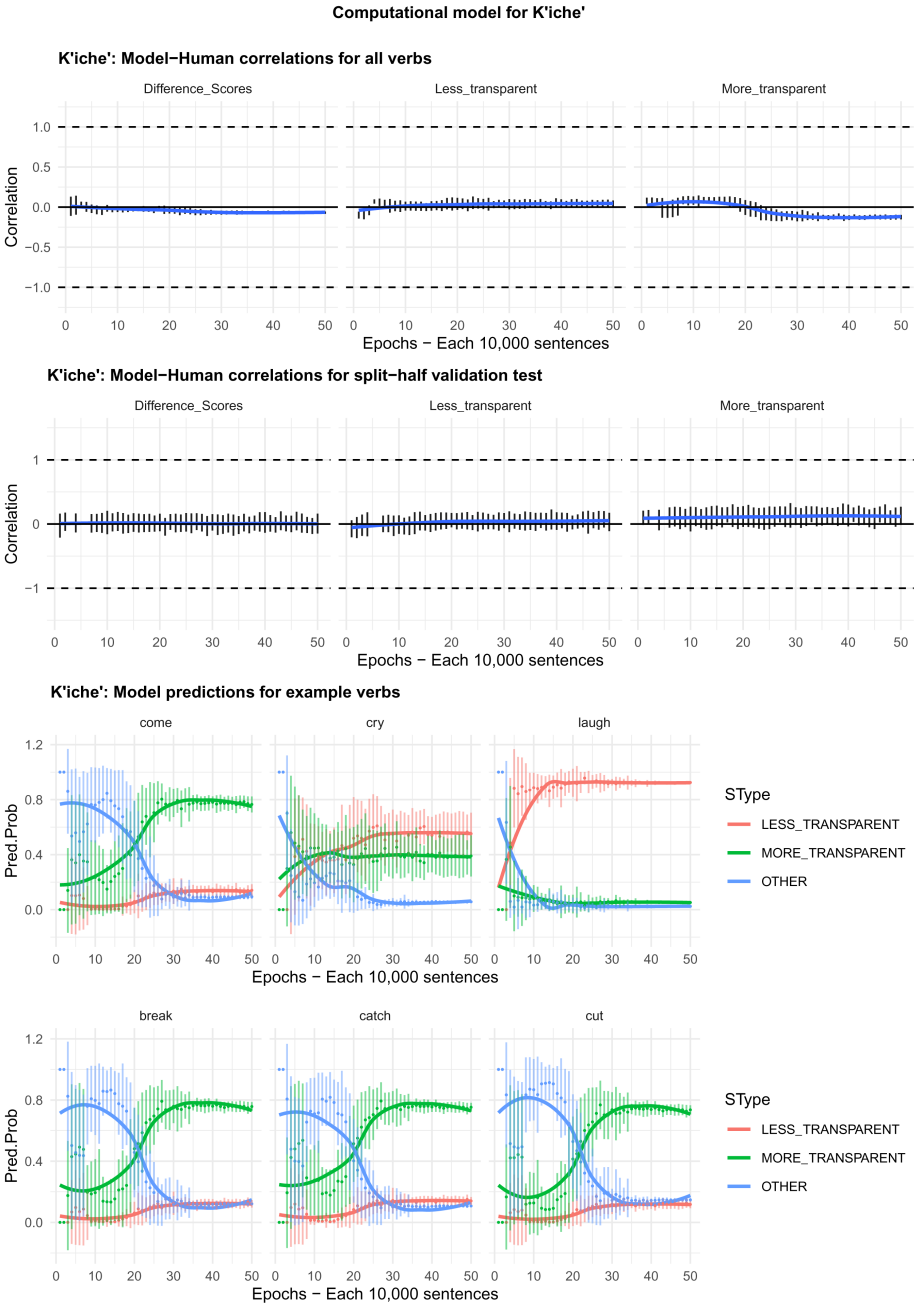
Model-child correlations for K’iche’ binary judgment data.

In general, the model does a good job of predicting children’s binary judgment data, though less so than for adults’ continuous judgment data ( Ambridge *et al.*, 2020, reported correlations mainly in the region of *r*=0.75). For the present binary judgment data, focussing on difference scores, the model achieved correlations in the region of *r*=0.5- *r*=0.6 for the English, Hebrew and Hindi child data, both for seen verbs and in the split-half validation test. All six correlations are comfortably statistically significant at *p*<0.01 (Critical *r* [df = 58] value for *p* < 0.05 = 0.21; for *p* < 0.01 = 0.30 [one tailed]). The model fares less well at predicting the raw proportions of “acceptable” judgments for less- and more- transparent causative forms; though with *r* values in the region of *r*=0.25- *r*=0.5, all twelve correlations are again statistically significant.

 For Japanese and K’iche’ the model achieves only one significant correlation, for more-transparent causative forms in Japanese. The poor performance of the K’iche’ model was to be expected on the basis of Ambridge *et al.* (2020) who found similar results for adults, which they attributed to difficulties with obtaining reliable corpus counts and semantic ratings. Additionally the poor performance of both the Japanese and K’iche’ models probably reflects the fact that – as noted above – both the Japanese- and K’iche’ speaking children seemed to be overly affected by valance when giving acceptability judgments.

Despite the apparent success of the computational model (at least for English, Hebrew and Hindi), it is important to note that it does not in fact explain development, or the retreat from overgeneralization, at least at the verb-by-verb level. At the macro level, the model does incorrectly predict the less- over more-transparent causative form for some verbs that prefer the latter (e.g., English *come, cry* and *laugh*) before correctly flipping its preference (see the bottom panels of Figure 4– Figure 8). However, at no stage does the model predict children’s judgments better than it does adults’ judgments. Indeed, if anything, it is a better model of the adult end state than it is of a child in the throes of overgeneralization (recall that Ambridge *et al.*, 2020, reported model-human correlations mainly in the region of *r*=0.75 for adults as compared to only around *r*=0.5 here). Then again, given the close correlation between adult and child judgments reported above, it may be that, at least by age 4–5, there is very little true overgeneralization – as opposed to across-the-board acceptance in a judgment task – for the model to explain.

### Discussion: Binary grammaticality judgments (4;0-5;0)

Data from the binary judgment task show that, with the apparent exception of Japanese, children aged 4;0-5;0 are capable of providing meaningful grammatical acceptability judgments for sentences containing more- and less-transparent causative verb forms, though they also show some evidence of judgments that correspond to overgeneralization errors. The computational model developed by Ambridge *et al.* (2020) successfully explained children’s judgment data for English, Hebrew and Hindi. Its failure to do so for K’iche’ and Japanese appears to be largely attributable to valance effects in children’s judgment data. However, these findings leave unanswered three questions: (1) Do children learning each of these languages actually produce these types of overgeneralization errors and, if so, (2) Can the computational model developed by Ambridge *et al.* (2020) explain their by-verb patterning and – crucially – (3) their development (i.e., the retreat from overgeneralization)?

## Study 2: Elicited production (4;0-5;0 and 5;6-6;6)

### Methods

**Preregistration.** As for Study 1, the sample size, materials, data collection methods and analysis plan were pre-registered at https://osf.io/qhnjk before data collection began. Again, we depart here from our data-analysis plan in order to test the computational model of Ambridge *et al.* (2020) which we judge to supersede the single-process theories tested in our original pre-registration.

**Computational model.** As for Study 1, the model architecture was identical to that reported in Ambridge *et al.* (2020) though new model runs were conducted (again, 48 runs for each of 50 epochs, for each language).

**Participants.** As per our preregistration, we recruited 48 children at each of ages 4;0-5;0 and 5;6-6;6 for each language (including K’iche’). Children were recruited from the same populations as Study 1, though none took part in both studies. Sample size criteria, eligibility criteria, and sources and methods of participant selection were the same as for Study 1.

**Stimuli and materials**. This study used a priming methodology, in order to encourage children to attempt to produce both less- and more-transparent causative forms for each of 60 target verbs (the same set used in Study 1 and Ambridge *et al.*, 2020). For each language, a further 60 verbs – 30 each that prefer the more- and less-transparent causative form – were selected for use as prime verbs, and corresponding animations created (following the same format as the animations for the target verbs). Only 60 prime verb were necessary, because – as for Study 1 – each child completed only half of the total number of target trials: That is, for each of 30 verbs – according to eight counterbalance lists – children described a causal animation following priming with (a) a more-transparent causative and (b) a less-transparent causative. As for Study 1, children completed two separate sessions. For each child, 16 (or 14) of the verbs appeared following both more- and less-transparent causative primes in the same session; the remaining 16 (or 14) appeared following a more-transparent causative prime in one session and a less-transparent causative prime in the other.

**Procedure.** Data were collected between January 2018 and March 2020 in schools and nurseries in the UK, Israel, India, Japan and Guatemala. A video of the production priming procedure can be found at https://osf.io/hqr9p/. Again, the procedure, is best summarized by the instructions that were given to children (in translation):

We are going to play a game. We’re going to watch some short videos and take it in turns telling this dog what has happened. The dog has either my card or your card: If we hear this sound **[plays howl sound effect]** then he has mine, if we hear this **[plays bark sound effect]** then he has yours. Then we can put our card on the grid and whoever fills their grid first wins the whole game. Our videos are going to look a bit like this. There is a stage like one you would see in a theatre with big red curtains **[plays an example animation:** ***dress*]**. So, as you can see, there was a little boy on the stage and he has no top on **[shows still of the stage at the beginning]** and when the curtains reopened he had a top on **[shows still of the stage at the end**]. You must imagine that when the curtains are closed that there is someone behind the curtains **[shows the closed curtains]**. So, in this one there was someone behind the curtains that did something to get the boy dressed. Let’s start with some practice ones and I’ll help you:
**Practice trial 1 – (** ***dress*** **and** ***wrap*)**
Experimenter: “someone dressed the boy”
Experimenter: “someone wrapped the present” **[encourages child to repeat]**
**Practice trial 2 – (** ***hiccup*** **and** ***jump*)**
Experimenter: “someone made the boy hiccup”
Experimenter: “someone made the boy jump” **[encourages child to repeat]**
**Practice trial 3 – (** ***free*** **and** ***close*)**
Experimenter: “someone freed the boy” **[waits for/encourages child to produce…]**
Child: “Someone closed the door” **[experimenter corrects if necessary]**
**Practice trial 4 – (** ***burp*** **and** ***drink*)**
**Experimenter**: “someone made the boy burp” **[waits for/encourages child to produce…]**
Child: “someone made the boy drink” **[experimenter corrects if necessary]**

The child and experimenter then completed the test trials in the same way. Note that the training trials were designed to give the child practice at producing less- and more-transparent causative forms following less- and more-transparent causative primes respectively. As for Study 1, the training verbs/animations did not feature in the test trials, and the barking/howling sound effects were automatically triggered by the software displaying the animations (Processing 2; https://processing.org/), such that the child completed her grid and won the game on the final trial of each day. Children’s responses were coded as to whether they included a more-transparent or less-transparent form of the target verb, with all other responses (e.g., intransitive use of the target verb; use of a different verb; no response) treated as missing data.

**Analysis.** All analyses were conducted in *R* (version 3.6.3; R Core Team, 2020). All computational models were built using the *nnet* package (version 7.3-14; Venables & Ripley, 2002). Correlations were conducted using the *cor* function of base R. All plots were made using *ggplot2* (version 2.2.1; Wickham, 2016).

### Results: Elicited production (4;0-5;0 and 5;6-6;6)

As for Study 1, before proceeding to test the computational model, it is instructive to compare children’s data against the gold-standard adult continuous judgment data reported by Ambridge *et al.* (2020) in order to determine (a) whether children’s productions generally seem to follow the constraints of the adult grammar and (b) whether they nevertheless produce overgeneralization errors that correspond to those observed (for English) in naturalistic data.

**Figure 9.**
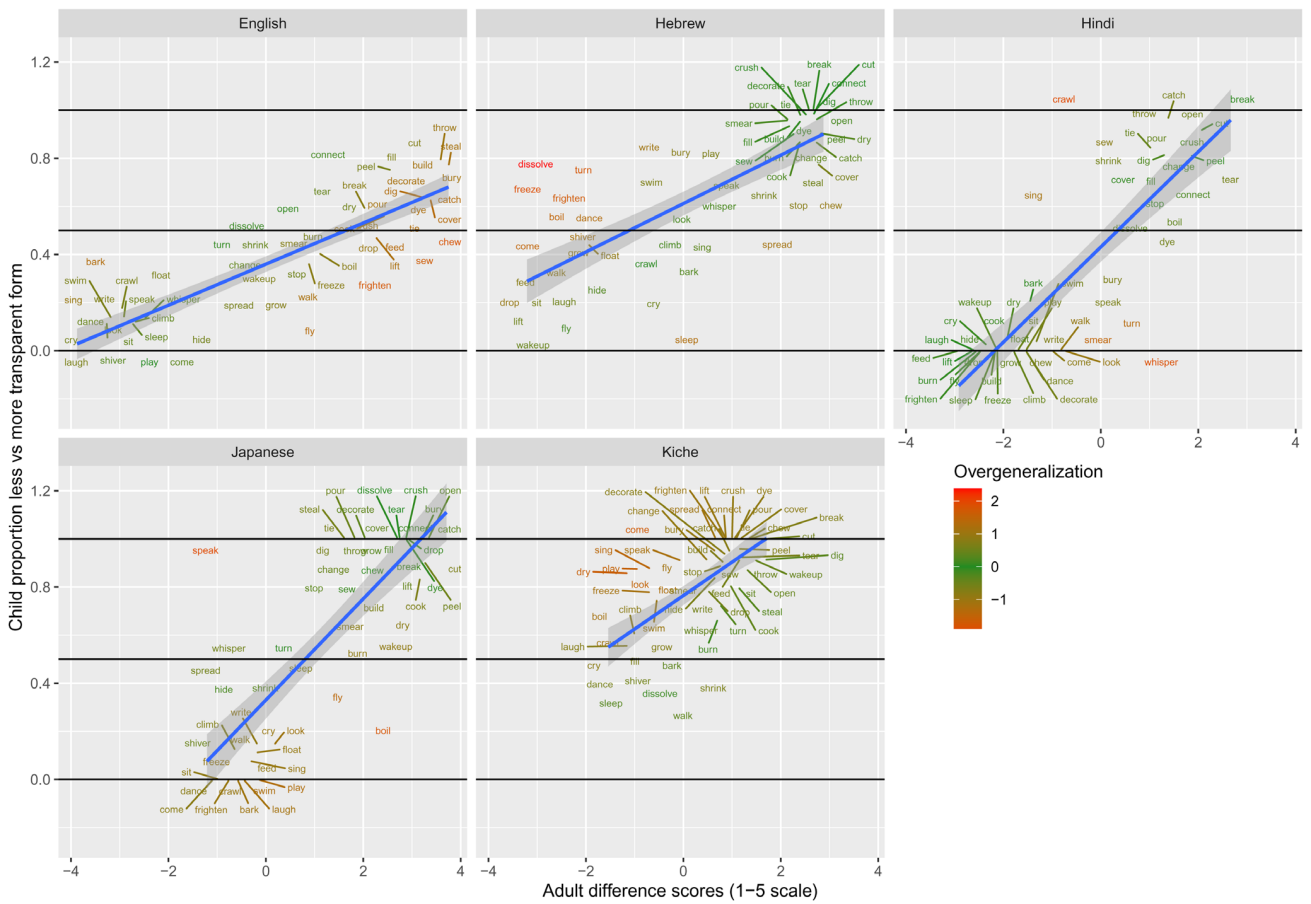
Children’s (4;0-5;0) elicited productions (present study) versus adult continuous judgments.

**Figure 10.**
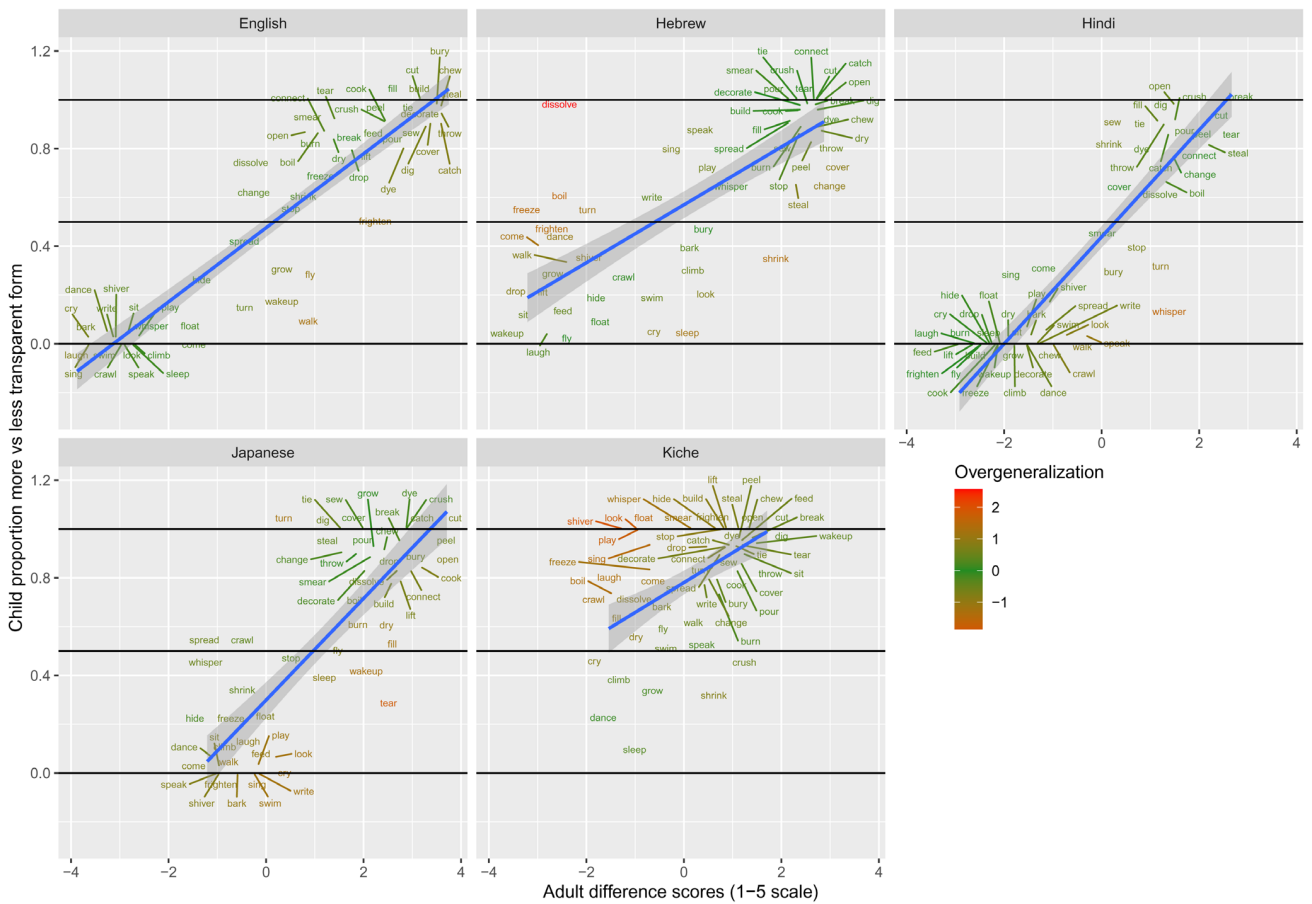
Children’s (5;6-6;6) elicited productions (present study) versus adult continuous judgments.

These data are plotted in Figure 9 (children aged 4;0-50) and Figure 10 (children aged 5;6-6;6). The x-axis shows, for each verb form, adults’ mean difference score (preference for less-over more-transparent causative forms). The y-axis shows the proportion of trials on which children, as a group, produced the less- versus more-transparent causative form of each verb (recall that all other responses were discarded as missing data).

Overgeneralization errors, this time in production, are colour coded in the same way as for Study 1 (i.e., green=adultlike, red=overgeneralization in either direction). As for the binary judgment data difference-scores analysis, overgeneralization errors are – on the whole – notable mainly by their absence, particularly for the older children. Such errors do occur. For example, around 20% of English 4–5-year olds’ causative forms with *sing* used the less transparent form (e.g., *Someone sang the boy), which is highly dispreferred for adults (c.f., *Someone made the boy sing*). Conversely around 20% of English 4–5-year olds’ causative forms with *throw* used the more transparent form (e.g., *Someone made the ball throw), which is highly dispreferred for adults (c.f. *Someone threw the ball*). Nevertheless, even for these more error-prone verbs, performance was largely adultlike, with around 80% of 4–5-year-olds’ responses using the preferred adult form. The picture was similar across languages with only a handful of verbs (e.g., *dissolve* for Japanese, *crawl* and *whisper* for Hindi, *speak* and *boil* for Japanese, *come, play* and *sing* for K’iche’) dramatically deviating from the adult reference point, when aggregating across 4–5-year-old children. By age 5–6, the picture looks even more adultlike, with Hebrew *dissolve* the only real exception.

These findings suggest that, as we tentatively concluded on the basis of the binary judgment data, children’s underlying grammatical knowledge in this domain is essentially adultlike by age 4-5, although at least some children have a higher tolerance than do adults – in production as well as judgments – for forms that deviate from this underlying grammar.

In order to verify this pattern statistically, we again ran a series of mixed-effects models in lme4, this time with the following (example) syntax:

(DV ~ Adult_Less_Transparent*AgeGroup +prime_type+ Valence +(0+AgeGroup|verb) + (1+Adult_Less_Transparent||participant)+ (1+prime_type||participant) , data=English, family="binomial"(link="logit"), control=glmerControl(optimizer = "bobyqa", optCtrl = list(maxfun = 2e5)))

The binary dependent variable is whether the child produced a less-transparent (1) or more-transparent (0) form of the target verb on each trial, with all other responses excluded as missing data. The most important fixed-effect predictor is the mean adult rating (scaled and centred) for (a) less-transparent causative forms, (b) more-transparent causative forms or (c) difference scores (three separate models for each language, with the dependent variable identical in each case). We also included the interaction of this predictor by age group (4–5, 5–6) in order to investigate whether, as it appears from Figure 9– Figure 10, the adult judgement scores predict the production data of the older children better than those of the younger children (i.e., whether older children are more closely aligned with the adult standard). Prime type (reflecting whether the experimenter primed the child with a less-transparent or more-transparent causative form on the relevant trial) and verb valence (as per the binary judgment study) were included as control predictors, but as main effects only, since we have no particular predictions regarding possible interactions between these predictors and age group and/or adult ratings; and the interactions would over-complicate the model. The binary predictors of age group and prime type were coded as −0.5/+0.5 (effect/sum/deviation/contrast coding; as opposed to the R default of dummy/treatment/baseline coding) in order to allow them to be interpreted as “ANOVA-style” main effects.

With regard to random effects, our goal was again to strike a balance between maximal and overly complex model structures. The model did not include a random intercept for verb, since valance (already included as a main effect) is already unique for each verb. A by-verb random slope for age group was included, as it is plausible that the effect of age group differed by verb. A by-verb random slope for prime type was tried, but ultimately excluded on the basis that it explained almost no variance (i.e., the effect of prime type does not appear to differ by verb). The model included random intercepts for participant, and by-participant random slopes for (a) the adult rating (less-transparent/more-transparent/difference scores, depending on the model) and (b) prime type, as it is important to account for possible by-participant variation here (especially for the key predictor of adult rating). In the interests of parsimony, the slope-intercept correlation was not included in either case.

The models are summarized in Table 6– Table 8. Focussing on difference scores ( Table 8), the adult continuous judgments are highly significantly predictive of children’s production data for all languages (with the adult difference score predictor significant at *p*=0.001 or better in each case). However, only for English ( *p*<0.001) and Hebrew ( *p*=0.02) was there any evidence of an interaction such that the adult continuous judgments are more predictive of older than younger children’s production data.

**Table 6.** Production task. Mixed effects models for less-transparent forms.

	*Est*	*SE*	*Z*	*p(z)*
**English**				
(Intercept)	-0.88	0.24	-3.75	**0.000**
Adult_Less_Transparent	2.87	0.23	12.35	**0.000**
AgeGroup1	0.61	0.35	1.74	0.081
prime_type1	-1.73	0.12	-14.88	**0.000**
Valence	-0.07	0.14	-0.52	0.604
Adult_Less_Transparent: AgeGroup1	2.32	0.30	7.60	**0.000**
**Hebrew**				
(Intercept)	1.20	0.28	4.31	**0.000**
Adult_Less_Transparent	1.01	0.25	4.01	**0.000**
AgeGroup1	-0.07	0.19	-0.37	0.710
prime_type1	0.06	0.09	0.74	0.459
Valence	-0.20	0.24	-0.82	0.412
Adult_Less_Transparent: AgeGroup1	0.41	0.16	2.63	**0.008**
**Hindi**	-3.07	0.62	-4.92	**0.000**
Adult_Less_Transparent	1.80	0.62	2.91	**0.004**
AgeGroup1	-0.37	0.38	-0.97	0.330
prime_type1	-0.68	0.15	-4.41	**0.000**
Valence	-1.12	0.59	-1.90	0.058
Adult_Less_Transparent: AgeGroup1	0.49	0.34	1.45	0.148
**Japanese**	0.40	0.31	1.28	0.199
Adult_Less_Transparent	2.50	0.35	7.17	**0.000**
AgeGroup1	-0.20	0.25	-0.78	0.434
prime_type1	-0.37	0.16	-2.36	**0.018**
Valence	-0.08	0.30	-0.27	0.787
Adult_Less_Transparent: AgeGroup1	0.13	0.26	0.49	0.627
**K'iche'**	2.23	0.21	10.79	**0.000**
Adult_Less_Transparent	0.56	0.16	3.49	**0.000**
AgeGroup1	-0.01	0.26	-0.04	0.964
prime_type1	-0.04	0.17	-0.23	0.818
Valence	0.06	0.17	0.34	0.733
Adult_Less_Transparent: AgeGroup1	0.05	0.16	0.31	0.757

**Table 7.** Production task. Mixed effects models for more-transparent forms.

	*Est*	*SE*	*Z*	*p(z)*
**English**				
(Intercept)	-0.49	0.27	-1.79	0.073
Adult_More_Transparent	-2.23	0.24	-9.30	**0.000**
AgeGroup1	1.09	0.36	3.02	**0.003**
prime_type1	-1.76	0.12	-14.54	**0.000**
Valence	-0.10	0.14	-0.69	0.491
Adult_More_Transparent: AgeGroup1	-1.73	0.27	-6.41	**0.000**
**Hebrew**				
(Intercept)	1.17	0.27	4.28	**0.000**
Adult_More_Transparent	-1.18	0.28	-4.27	**0.000**
AgeGroup1	-0.09	0.20	-0.44	0.656
prime_type1	0.07	0.09	0.80	0.423
Valence	-0.20	0.22	-0.89	0.375
Adult_More_Transparent: AgeGroup1	-0.28	0.18	-1.56	0.119
**Hindi**				
(Intercept)	-2.99	0.63	-4.76	**0.000**
Adult_More_Transparent	-1.60	0.57	-2.81	**0.005**
AgeGroup1	-0.36	0.40	-0.89	0.372
prime_type1	-0.66	0.15	-4.31	**0.000**
Valence	-1.15	0.59	-1.96	**0.050**
Adult_More_Transparent: AgeGroup1	-0.24	0.23	-1.02	0.306
**Japanese**				
(Intercept)	0.63	0.33	1.93	0.054
Adult_More_Transparent	-2.38	0.36	-6.62	**0.000**
AgeGroup1	-0.08	0.25	-0.33	0.739
prime_type1	-0.36	0.15	-2.36	**0.018**
Valence	-0.34	0.31	-1.12	0.263
Adult_More_Transparent: AgeGroup1	-0.14	0.25	-0.58	0.565
**K''iche'**				
(Intercept)	2.28	0.21	10.91	**0.000**
Adult_More_Transparent	-0.79	0.18	-4.38	**0.000**
AgeGroup1	-0.01	0.28	-0.03	0.978
prime_type1	-0.04	0.19	-0.21	0.834
Valence	0.03	0.16	0.18	0.855
Adult_More_Transparent: AgeGroup1	0.22	0.21	1.01	0.314

**Table 8.** Production task. Mixed effects models for difference scores.

	*Est*	*SE*	*Z*	*p(z)*
**English**				
(Intercept)	-0.69	0.20	-3.43	**0.001**
Adult_Difference_Score	2.85	0.20	14.61	**0.000**
AgeGroup1	0.93	0.32	2.90	**0.004**
prime_type1	-1.76	0.12	-15.02	**0.000**
Valence	-0.11	0.12	-0.93	0.352
Adult_Difference_Score: AgeGroup1	2.26	0.30	7.65	**0.000**
**Hebrew**				
(Intercept)	1.23	0.27	4.59	**0.000**
Adult_Difference_Score	1.20	0.25	4.76	**0.000**
AgeGroup1	-0.07	0.19	-0.38	0.703
prime_type1	0.06	0.09	0.74	0.458
Valence	-0.13	0.23	-0.56	0.575
Adult_Difference_Score: AgeGroup1	0.38	0.17	2.30	**0.022**
**Hindi**				
(Intercept)	-3.05	0.62	-4.94	**0.000**
Adult_Difference_Score	1.95	0.60	3.26	**0.001**
AgeGroup1	-0.29	0.38	-0.75	0.455
prime_type1	-0.68	0.16	-4.41	**0.000**
Valence	-1.06	0.58	-1.82	0.068
Adult_Difference_Score: AgeGroup1	0.38	0.31	1.24	0.216
**Japanese**				
(Intercept)	0.53	0.28	1.91	0.056
Adult_Difference_Score	2.71	0.32	8.52	**0.000**
AgeGroup1	-0.15	0.25	-0.63	0.532
prime_type1	-0.37	0.16	-2.36	**0.018**
Valence	-0.04	0.26	-0.14	0.891
Adult_Difference_Score: AgeGroup1	0.12	0.26	0.48	0.629
**K'iche'**				
(Intercept)	2.28	0.21	10.96	**0.000**
Adult_Difference_Score	0.71	0.17	4.27	**0.000**
AgeGroup1	-0.01	0.28	-0.04	0.970
prime_type1	-0.05	0.19	-0.27	0.788
Valence	0.09	0.17	0.55	0.581
Adult_Difference_Score: AgeGroup1	-0.05	0.19	-0.24	0.808

Interestingly, and unlike for some languages with regard to the binary judgment data, the valance predictor was never significant, except for a single model (the Hindi model predicting on the basis of more-transparent forms, and even then, only marginally so – *p*=0.05 – and with no correction for multiple comparisons).

Overall, then, the production findings mirror those of the binary judgment data: As a group, children’s production data seem to reflect generally adultlike knowledge (i.e., children’s production data are very well predicted by adults’ grammaticality judgment data). Although overgeneralization errors do occur, these are relatively rare and generally restricted to a handful of verbs. Again, echoing the binary judgment data, these findings suggest that – at least from around age 4–5 – such errors reflect not so much a deficit in the grammar, but more a deficit in inhibiting the production of overgeneralized forms.

**Figure 11.**
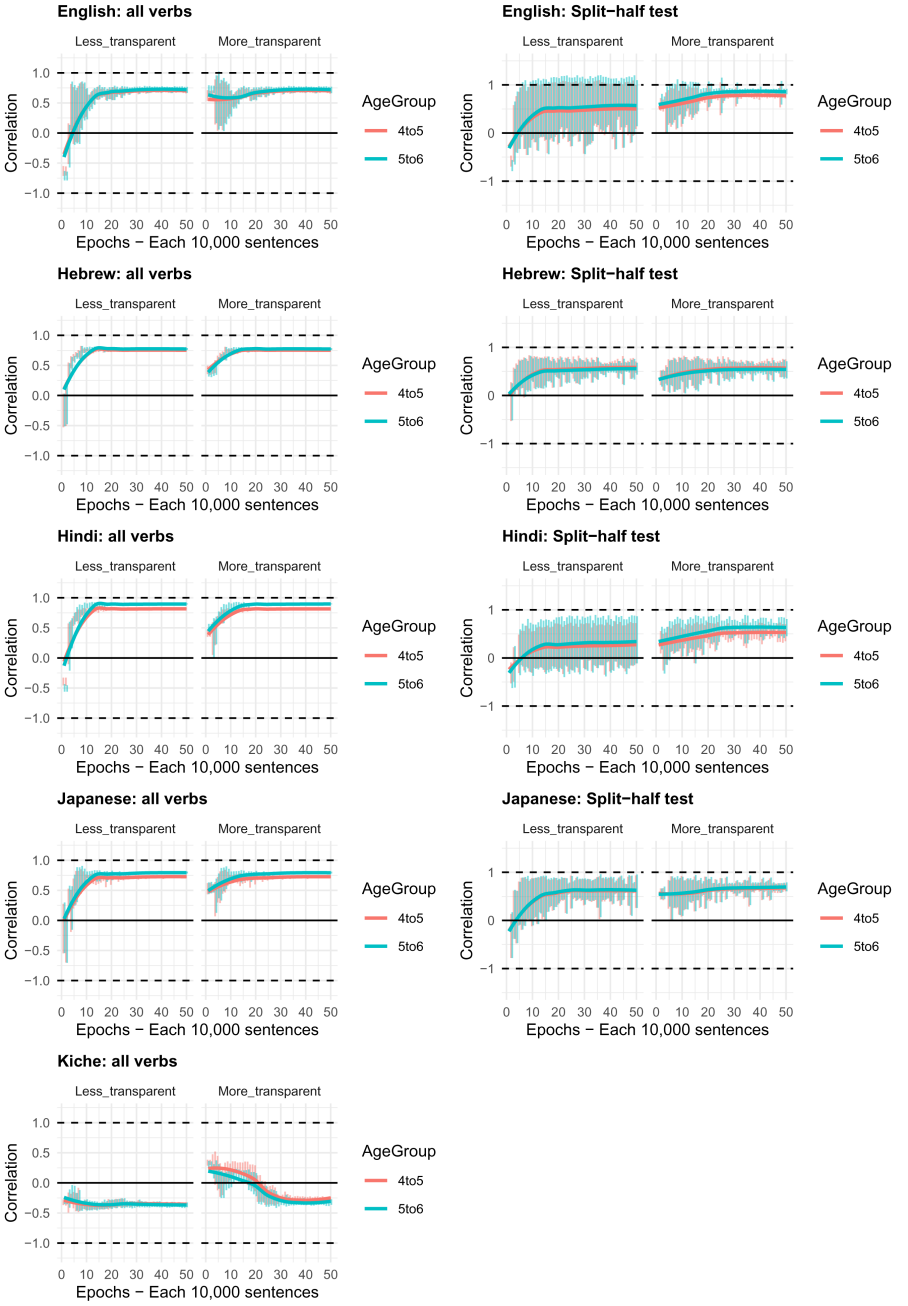
Model-child correlations for elicited production data.

Moving on to the tests of the computational model, Figure 11 plots – for English, Hebrew, Hindi, Japanese and K’iche’ respectively – model-child correlations for (a) the full set of 60 verbs, and (b) the split-half validation test (30 verbs, randomly selected for each run), as well as the developmental pattern shown by the model for a number of example verbs (again, recall that split-half test does NOT consist of training the model on half of the participants’ grammaticality judgments; no model is ever given access to these judgments). Separate correlations are run for less-transparent and more-transparent causative forms because, although these sum to 1 for children (since all other responses are treated as missing data), the same is not true for the model which has three output units, corresponding to less-transparent, more-transparent and “Other”. That said, since the model rapidly learns to predict “Other” forms with very low probability when interrogated for a causative form, the correlations for less- and more- transparent forms are extremely similar.

 For all languages except K’iche’, the model does an excellent job of predicting children’s judgment data with correlations upwards of *r*=0.75 for seen verbs, and *r*=0.5 for unseen verbs. Again, its poor performance with K’iche’ is likely attributable to difficulties with obtaining reliable corpus counts and semantic ratings ( Ambridge *et al.*, 2020). For this reason, we did not proceed to the split-half validation test for K’iche’. For the four other languages, however, the model’s ability at predicting children’s production data is on a par with its ability at predicting adults’ continuous judgment data ( Ambridge *et al.*, 2020).

The only notable shortcoming of the model is that although it simulates the overall generalization-then-retreat pattern shown by children (see Figure 4– Figure 8, bottom panels), it does not simulate the observed differences between the present 4;0-5;0 and 5;6-6;6 year olds (see Figure 9– Figure 10). That is, the model does not show an “immature” stage in which its predictions correspond more closely to the productions of the younger than the older children. This echoes the failure of the model to simulate the differences between children and adults observed in the grammatical acceptability judgment study above, and in Ambridge *et al.* (2020). Again, the most likely explanation seems to be that, at least by age 4–-5, there is very little true overgeneralization for the model to explain. Rather, children’s grammatical knowledge in this domain is largely adultlike; they are simply somewhat less reluctant than adults to accept or produce forms that deviate from that grammar.

### Discussion: Elicited production (4;0-5;0 and 5;6-6;6)

Data from the elicited-production task show that, children aged 4;0-5;0 and 5;6-6;6 not only produce causative overgeneralization errors (* *Someone sang / crawled / wrote / whispered / sang / slept / sat the boy*; c.f., *Someone made the boy/dog bark / sing / crawl* etc.) but do so in such a way that their by-verb patterning – except for K’iche’ – is well predicted by the computational model of Ambridge *et al.* (2020). At the same time, the model in its present form does not explain the retreat from overgeneralization *per se*, given that its verb-by-verb predictions are always a better fit for data from adults and older children (from Ambridge *et al.*, 2020) than for younger children (Studies 1- and 2 above). What is the reason for this failure?

One possibility, already discussed above, is that – at least in this domain – the grammatical knowledge of 4–5-year olds is (near-) adultlike, with non-adultlike performance a consequence of extralinguistic factors. For example, reduced executive function – as compared to adults – could result in a reduced ability to resist “tempting” errors, whether in judgments or production. An alternative possibility is that these errors really do reflect a non-adultlike grammar, but that the model – in its current form – does not simulate this deficit. In order to explore this possibility, we ran a series of new models with various limitations that might correspond to those experienced by real language learners.

## Study 3: Further computational modeling

Study 3 investigated the ability of a wide variety of models to simulate the binary grammatical acceptability judgment data from Study 1 (age 4;0–5;0), the elicited production data from Study 2 (ages 4;0–5;0 and 5;6–6;6) and the grammatical acceptability judgment data from Ambridge *et al.* (2020) (ages 5–6, 9–10 and adults). In particular, our goal was to investigate whether, by instantiating various limitations that correspond to those facing children, it might be possible to build a model whose verb-by-verb predictions correlate *better* with the judgment and production data of younger children than those of older children and adults. If so, this constitutes preliminary evidence that children’s retreat from overgeneralization is a consequence of the reduction of the relevant limitation. If not, this constitutes further evidence that children’s retreat from overgeneralization is a consequence of changes outside of the purview of this modeling, such as increasing executive function, which allow for better rejection and inhibition of ungrammatical forms.

Because the *nnet* package does not allow for automated exploration of model parameters – which is key to the present investigation – we switched to the Deep Learning packing of h2o.ai, running in the R environment (see Aiello, Eckstrand, Fu, Landry, Aboyoun, 2018, for a tutorial). Despite its name, the Deep Learning package allows for simple connectionist architectures similar to the *nnet* networks described above (although a minimum of one hidden layer is required). The main advantage of this package for our purposes is its grid search function, which trains a model for every combination of hyperparameter values specified by the user. This allows us to rapidly explore possible constraints on learning that might be similar to those present for real child learners. The basic task of the *h2o* models was the same as the *nnet* models described above: to learn verb-construction mappings based on a suitable input corpus, and then generate verb-construction predictions that can then be correlated with the relevant judgment and production data from children and adults. As for the two-layer models above, the results reported below are always averaged across 48 runs of each model with different initial weights (corresponding to 48 adult/child participants per human task).

### Methods

The hyperparameters explored were as follows (L1 and L2 regularization, and learning rate were all fixed at 0.01):

- **Architecture.** Two sets of models had a single hidden-unit layer, with 4 and 10 nodes respectively. Two sets of models had two hidden-unit layers, with (4,4) and (8,8) nodes respectively. Having fewer hidden units/layers reduces the ability of the model to memorize the training set (analogous to worse memory in children), forcing it to generalize more beyond the input.
- **Dropout.** Dropout also simulates memory and or processing limitations in children by randomly dropping a proportion of hidden units on a given trial. It is also known to aid generalization in both models and (perhaps especially when dreaming) humans ( Hoel, 2021). Two settings were used: 0 (i.e., no dropout) and 0.75.
- **Annealing.** Children’s learning starts out rapid and gradually slows, as they near the adult state. This is simulated by learning-rate annealing. Two settings were used: 0 (i.e., no annealing) and 0.75.
- **Epochs.** One obvious difference between children and adults is that the former have simply been exposed to less input. This is simulated by varying the number of epochs (each corresponding to 300,000 utterances): 2, 5, 15, and 50.

The following parameters were varied by hand:

- **Split-half.** As for the previous simulations, models were either (a) trained and tested on all 60 verbs or (b) trained on 30 verbs and tested on a held-out half, assessing their ability to generalize to unseen items (though, again, recall that verbs were never trained on human acceptability judgment or production data).
- **Semantics.** A question that remains unanswered by the previous simulations is the extent to which the models were making use of semantic similarity between verbs that tend to appear in similar constructions, as opposed to simply rote learning the training set. The equivalent question holds for children too: Although adult linguists can spot semantically based patterns, it might be that children learn verbs’ construction privileges on a verb-by-verb basis, without making semantically based generalizations. To explore this possibility, models were either (a) trained on human-supplied semantic ratings (as above) or (b) trained on randomized semantic ratings that removed all semantic systematicity in verb+construction cooccurrences, while maintain identical architecture and an otherwise-identical training set.
- **Adult- versus child-directed speech.** The previous models used frequency obtained from adult corpora (mainly subtitle and internet corpora), simply because these were the largest available. However, it may be that the speech children hear differs systematically in important respects. To explore this possibility, training sets were based on either (a) the original adult frequency counts (of more-transparent, less-transparent and “other” forms) or (b) equivalent counts taken from child-directed speech. This was possible only for the languages with corpora available on CHILDES ( MacWhinney, 2000): English, Hebrew and Japanese. In all cases, we combined all available CHILDES corpora, counting only child-directed (not child) speech. Nevertheless, the resulting combined corpora were relatively impoverished in terms of the relevant forms. For Hebrew, around 50% of verbs did not occur at all in either the more- or less-transparent causative form. For Japanese, the corresponding figure was 25%, though for the much larger English dataset, it was just 5% (three verbs: *dissolve*, *shiver* and *shrink*). Although the original child-directed corpora are much smaller than the adult-directed equivalents, the training sets were normalized, such that one epoch consists of 300,000 utterances, whether of child- or adult-directed speech. The lack of suitable child-directed corpora – and, in particular, corpora that are sufficiently dense as to capture any age-by-age variation in the relevant caregiver speech – is a limitation that should ideally be addressed in future research.

### Results

**Figure 12.**
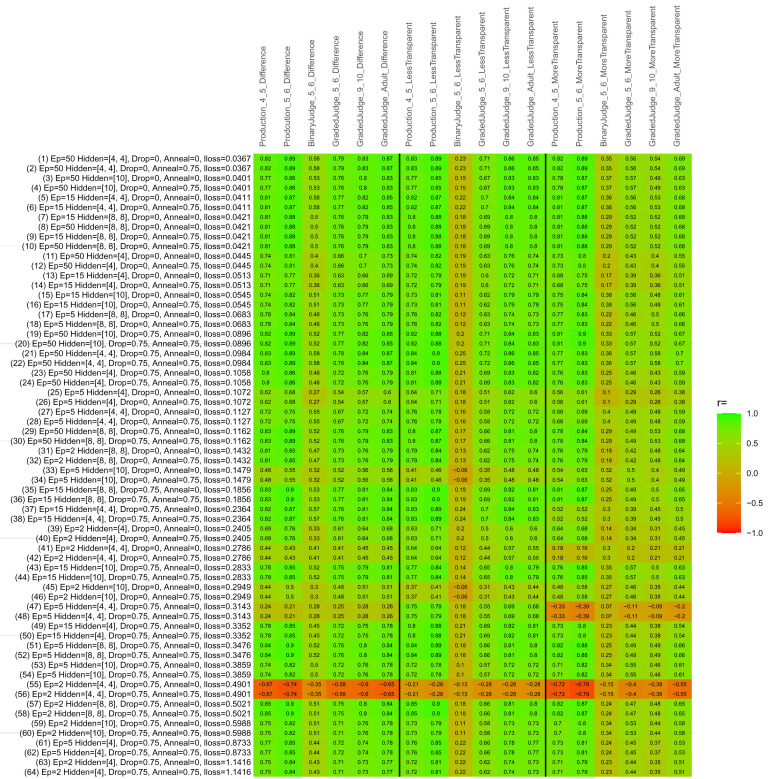
English: Model-human correlations (all verbs, adult-directed corpora).

The results for the models based on adult-directed corpora are summarized in Figure 12– Figure 16 (English, Hebrew, Hindi, Japanese, K’iche’ for all verbs), 17– 21 (split-half test), 22– 26 (no-semantics models, all verbs), 27– 31 (no-semantics models, split-half test). The results for the models based on child-directed corpora are summarized in Figure 32– Figure 34 (English, Hebrew, Japanese, for all verbs), 35– 37 (split-half test), 38– 40 (no-semantics models, all verbs), 41– 43 (no-semantics models, split-half test). The values shown are simple Pearson correlations between the model’s predictions and the adult/child production/judgment data labelled. The models are shown in order from best (top) to worst (bottom), according to log loss, though it is important to remember that this measure is calculated on the model’s learning of the corpus data, NOT participants’ judgment or production data, which it is never shown. Thus the “best” model in terms of log loss, is not necessarily the model that shows the highest correlation with human data (indeed, the models with lowest log-loss are at greatest risk of over-fitting the training corpora, potentially lowering their fit to human judgment and production data). Although these figures contain a huge amount of information, the main conclusions can be summarized quite simply.

**Figure 13.**
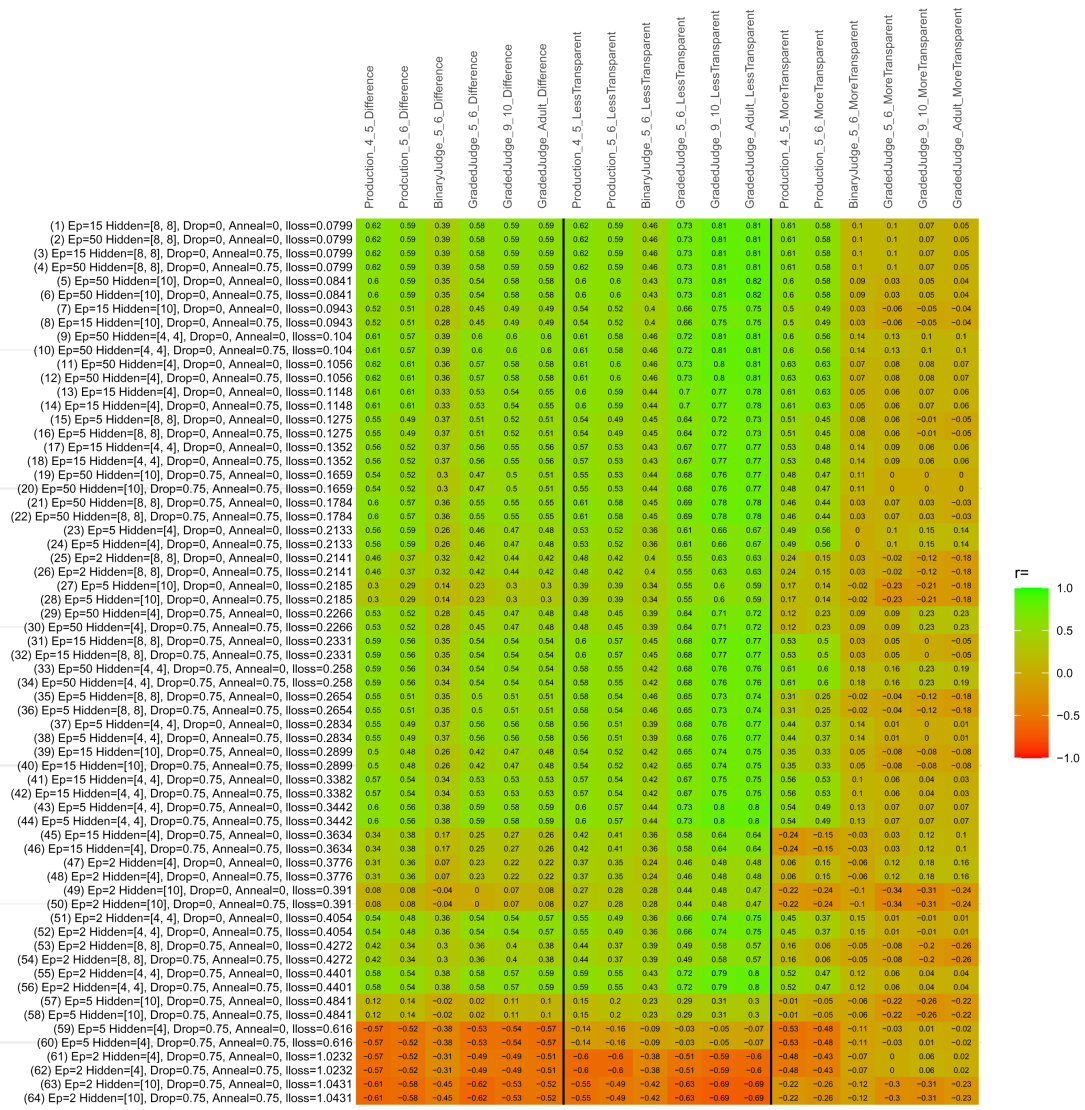
Hebrew: Model-human correlations (all verbs, adult-directed corpora).

**Figure 14.**
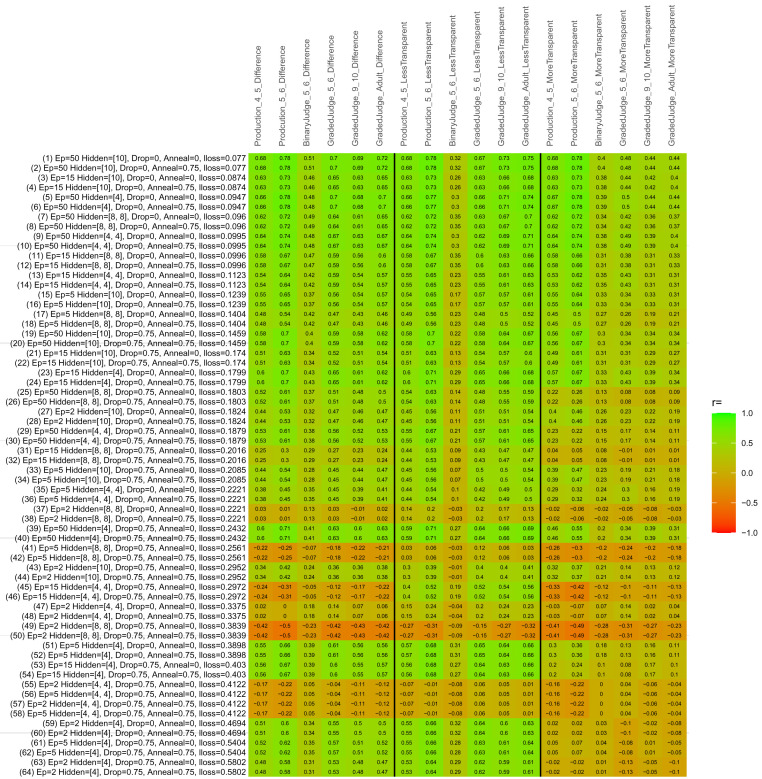
Hindi: Model-human correlations (all verbs, adult-directed corpora).

**Figure 15.**
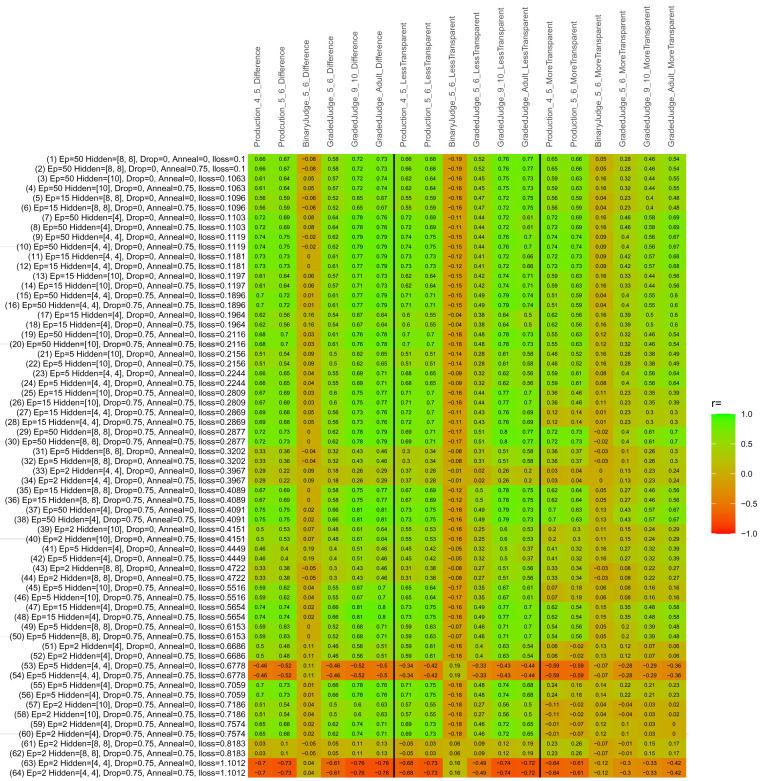
Japanese: Model-human correlations (all verbs, adult-directed corpora).

**Figure 16.**
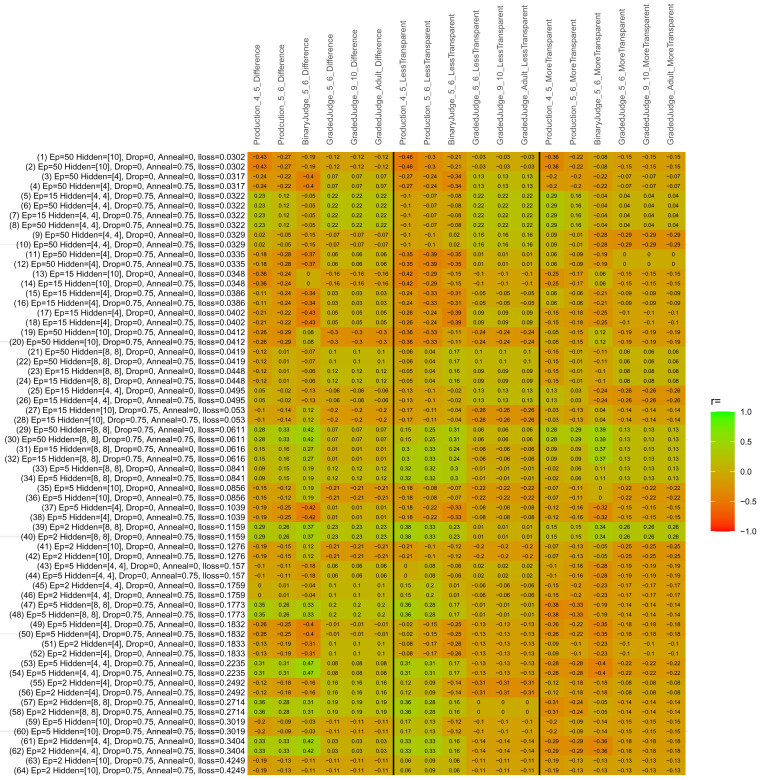
K'iche’: Model-human correlations (all verbs, adult-directed corpora).

**Figure 17.**
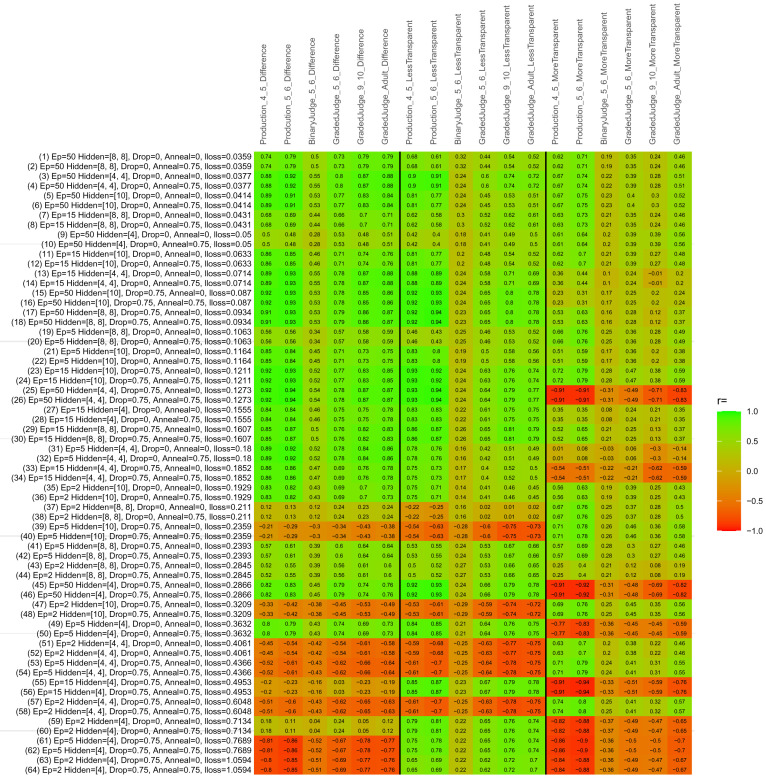
English: Model-human correlations (split-half test, adult-directed corpora).

**Figure 18.**
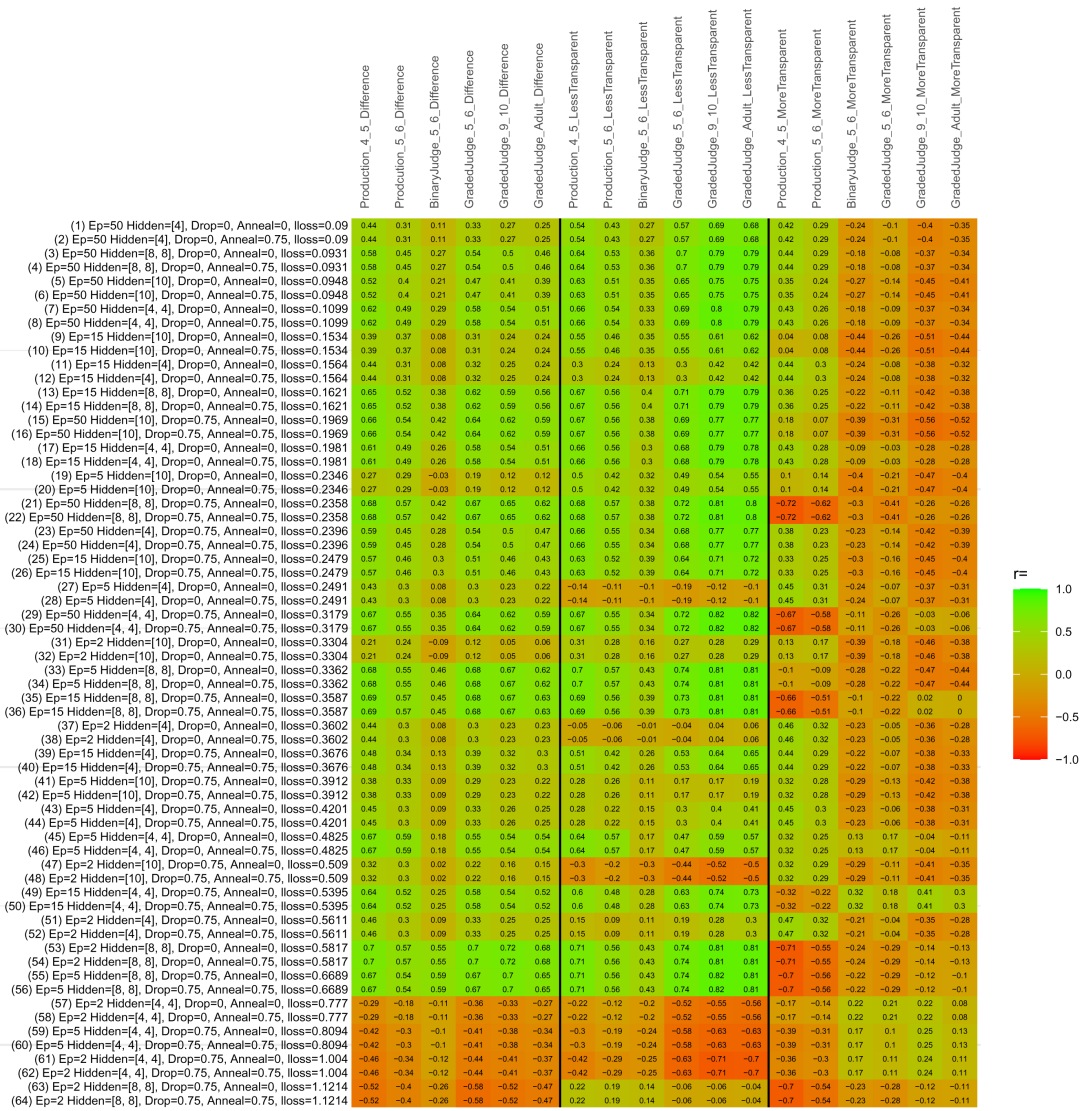
Hebrew: Model-human correlations (split-half test adult-directed corpora).

**Figure 19.**
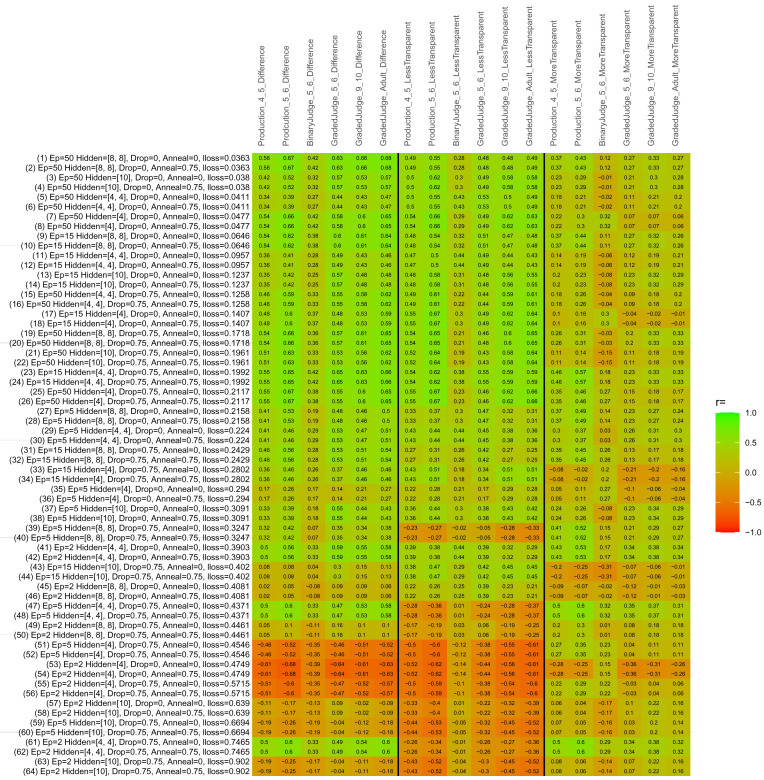
Hindi: Model-human correlations (split-half test, adult-directed corpora).

**Figure 20.**
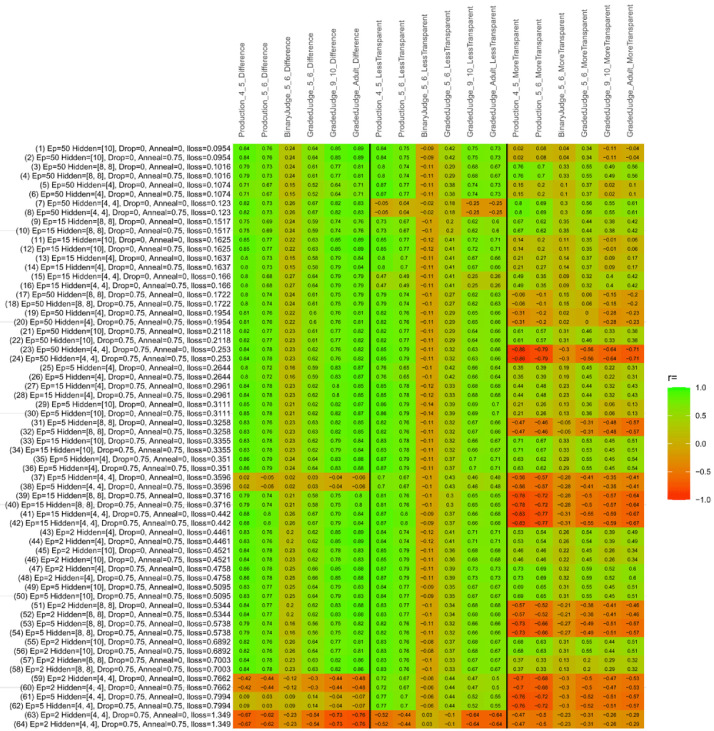
Japanese: Model-human correlations (split-half test, adult-directed corpora).

**Figure 21.**
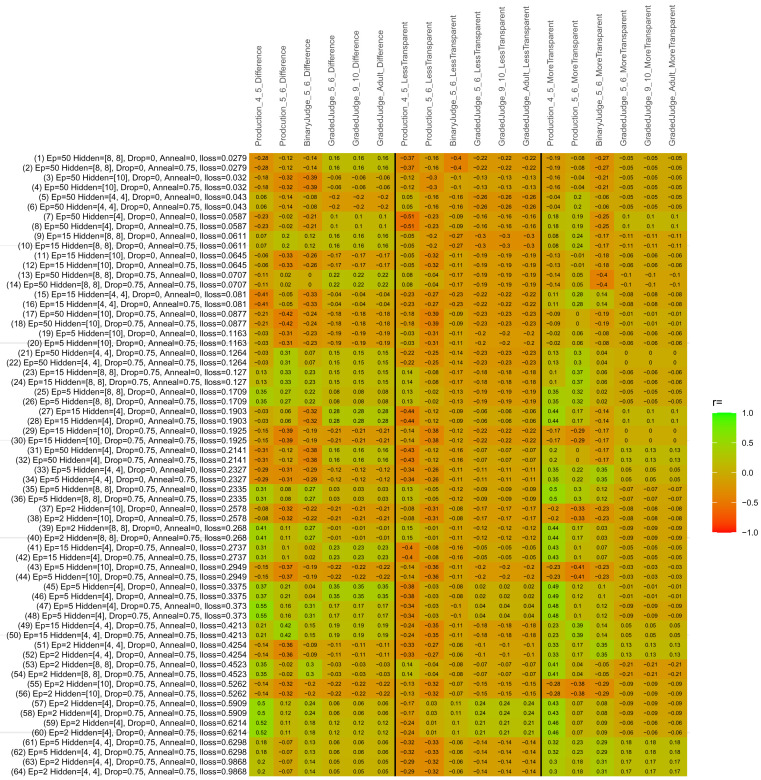
K’iche’: Model-human correlations (split-half test, adult-directed corpora).

**Figure 22.**
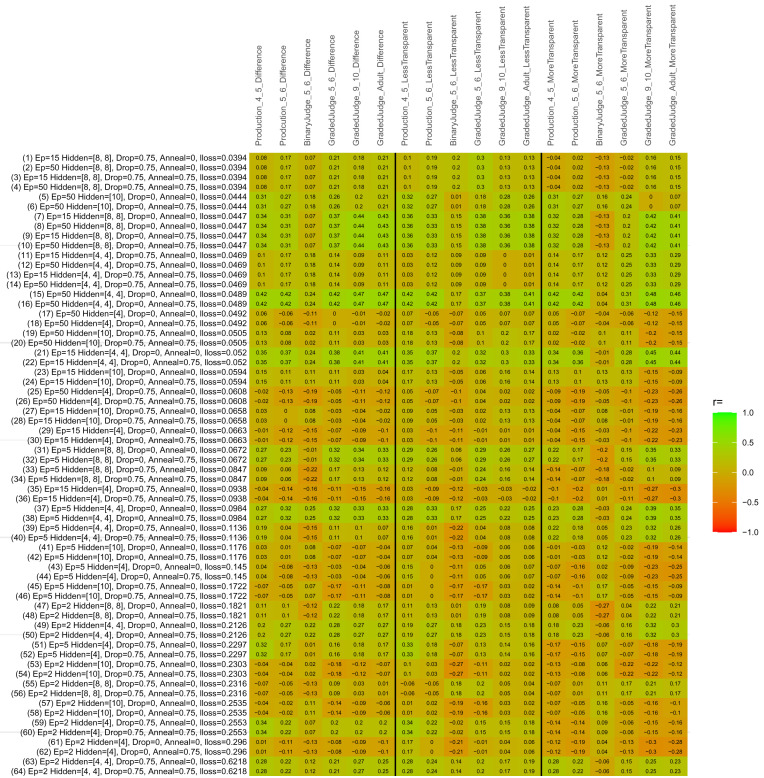
English: No-semantics-Model-human correlations (all verbs, adult-directed corpora).

**Figure 23.**
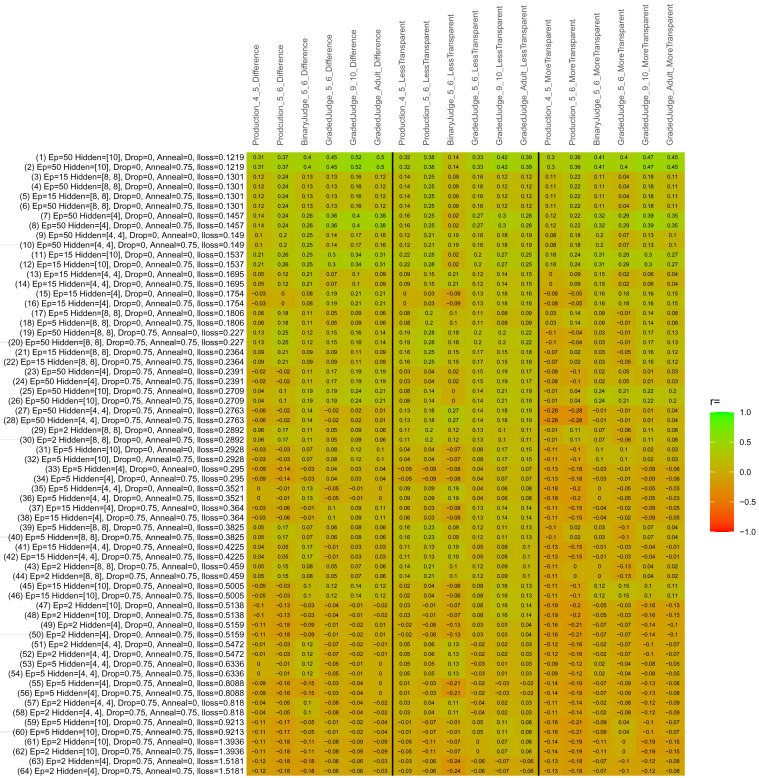
Hebrew: No-semantics-Model-human correlations (all verbs, adult-directed corpora).

**Figure 24.**
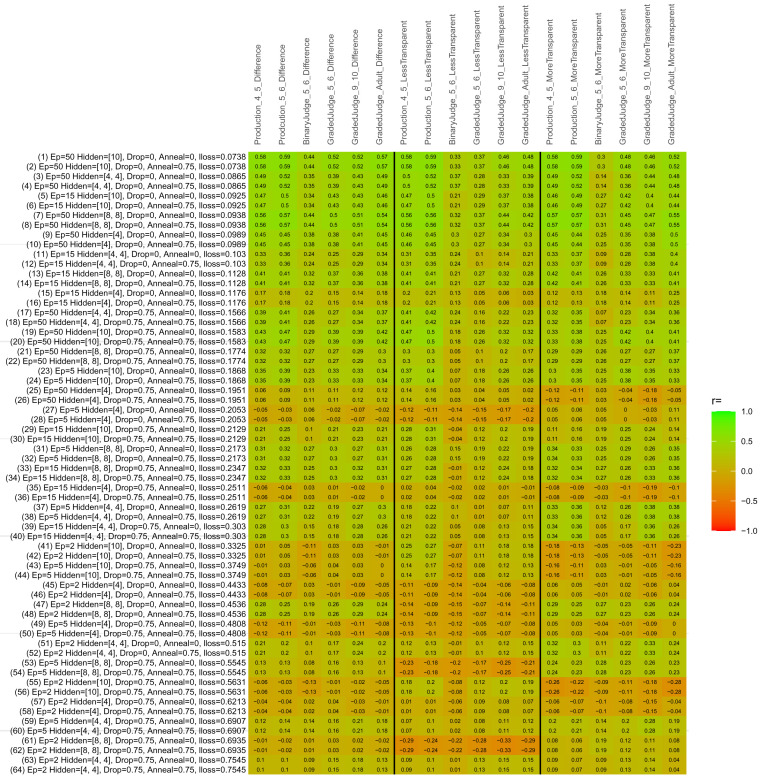
Hindi: No-semantics-Model-human correlations (all verbs, adult-directed corpora).

**Figure 25.**
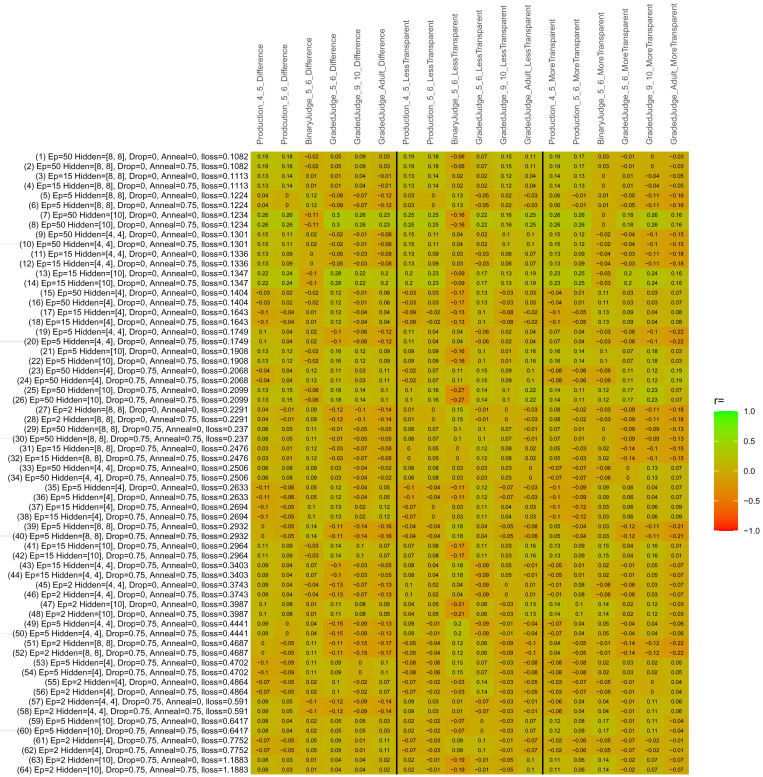
Japanese: No-semantics-Model-human correlations (all verbs, adult-directed corpora).

**Figure 26.**
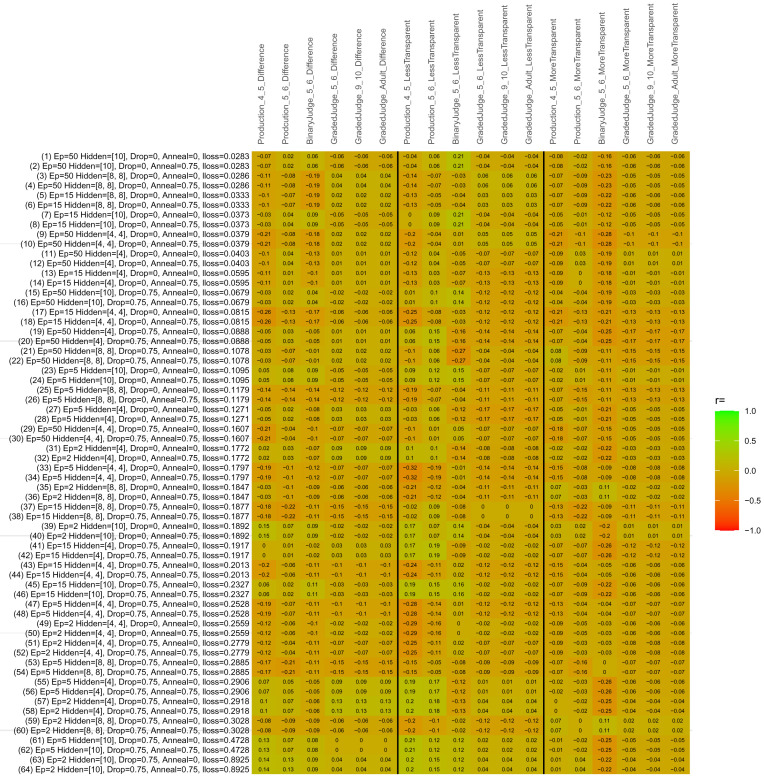
K’iche’: No-semantics-Model-human correlations (all verbs, adult-directed corpora).

**Figure 27.**
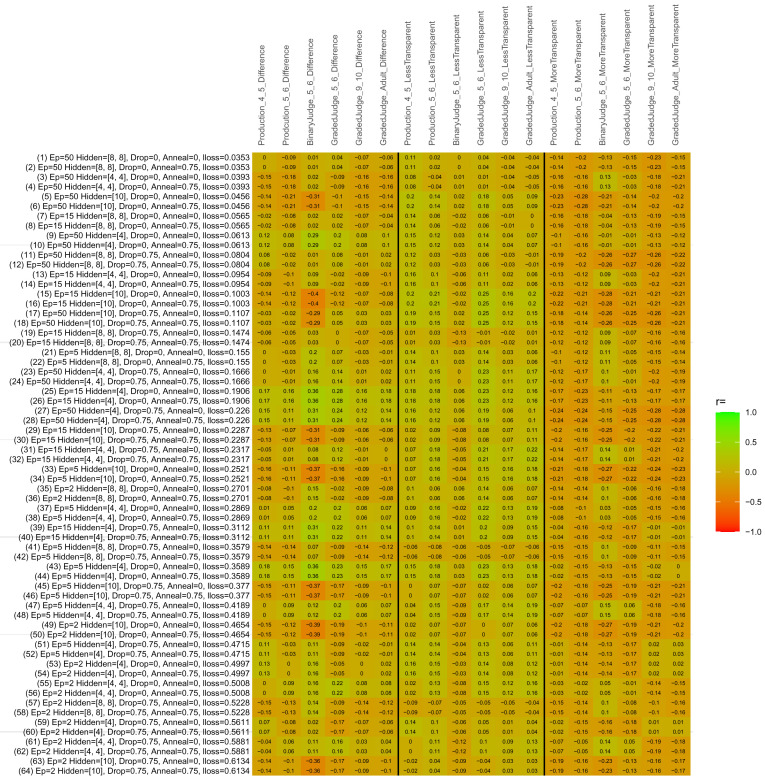
English: No-semantics-Model-human correlations (split-half test, adult-directed corpora).

**Figure 28.**
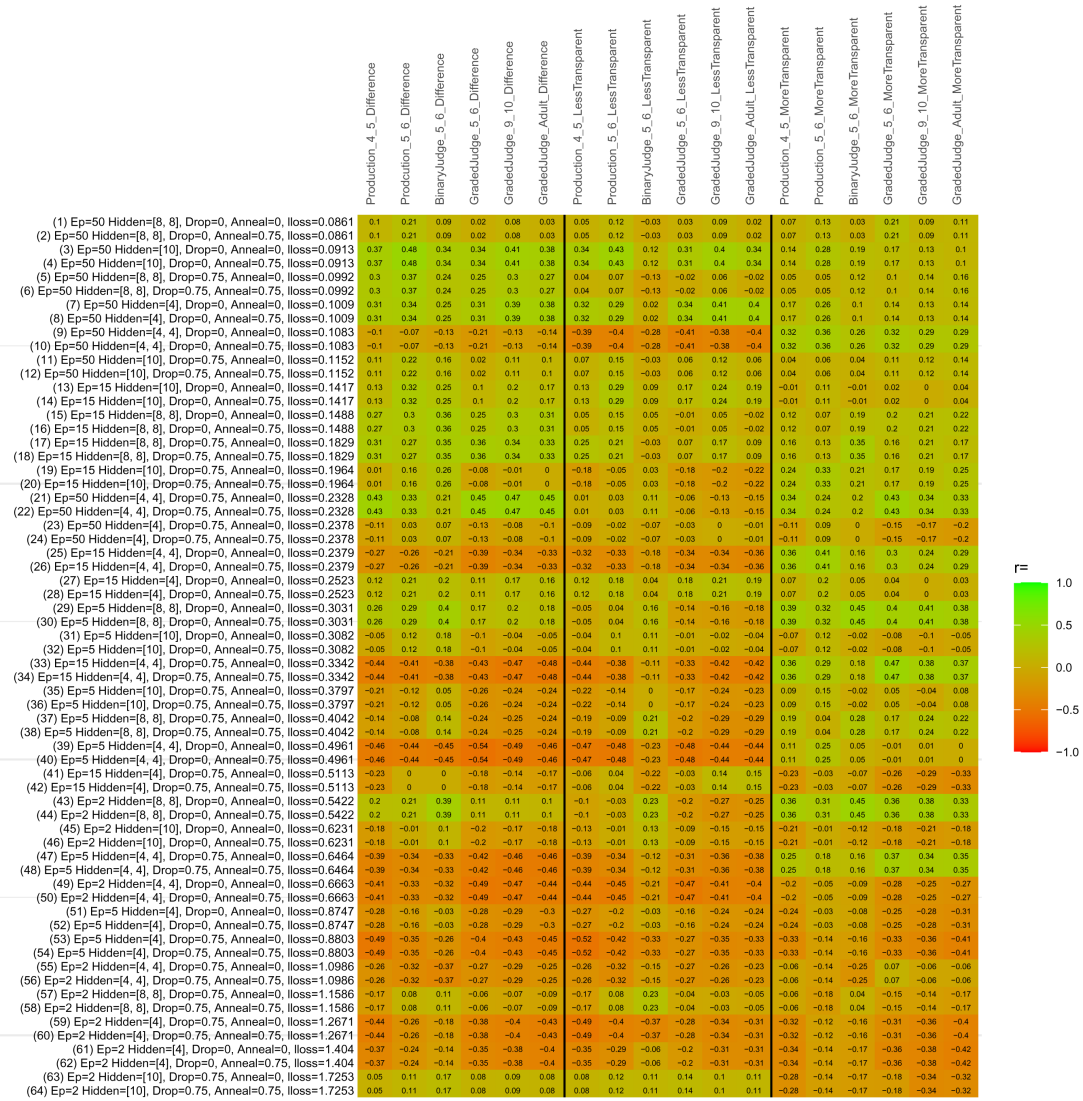
Hebrew: No-semantics-Model-human correlations (split-half test, adult-directed corpora).

**Figure 29.**
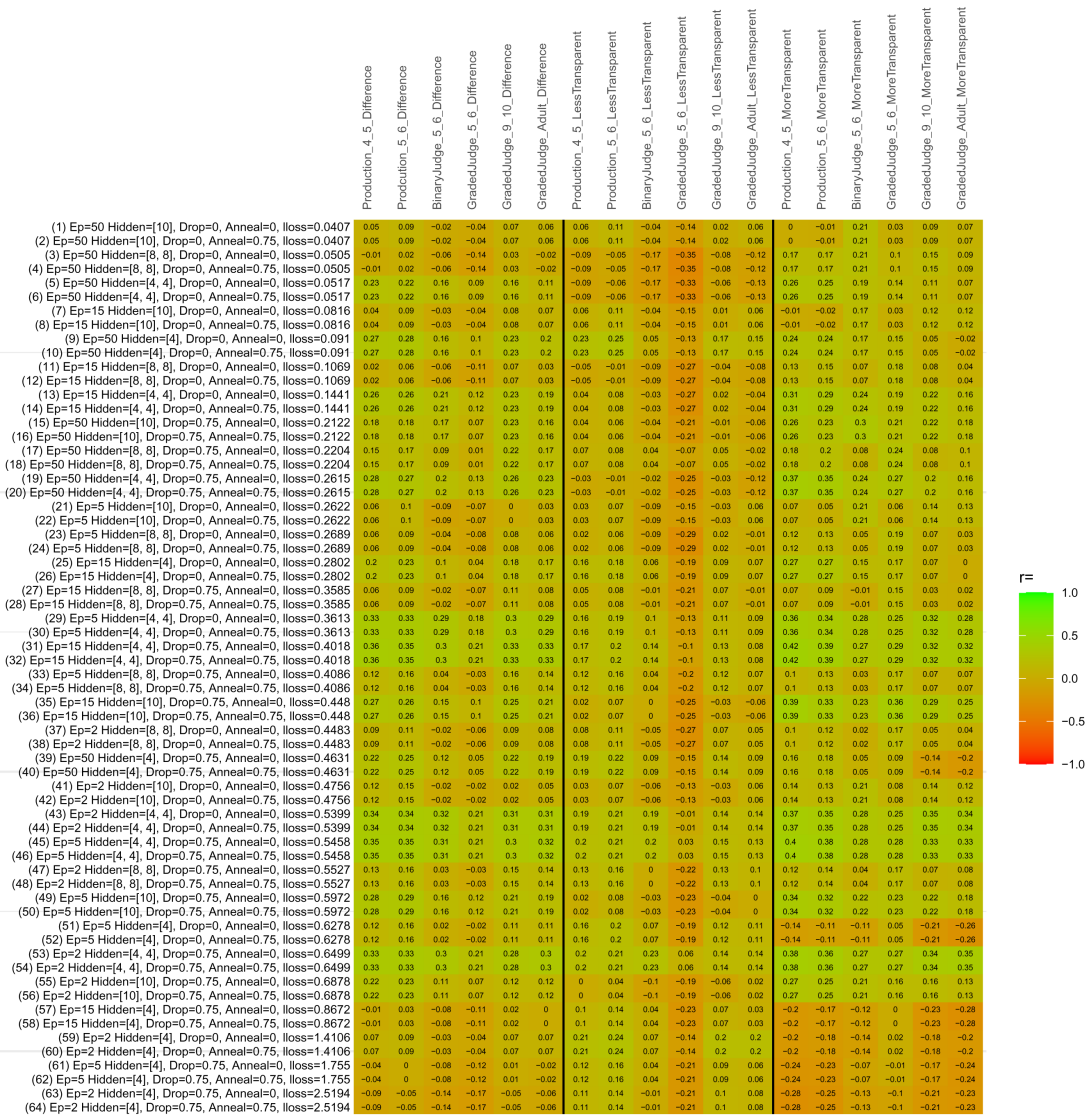
Hindi: No-semantics-Model-human correlations (split-half test, adult-directed corpora).

**Figure 30.**
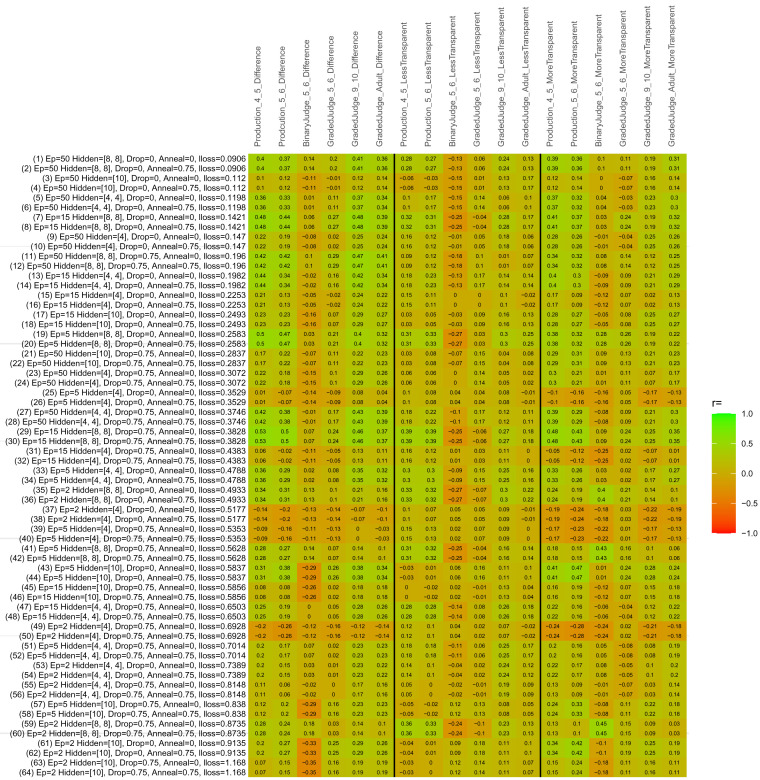
Japanese: No-semantics-Model-human correlations (split-half test, adult-directed corpora).

**Figure 31.**
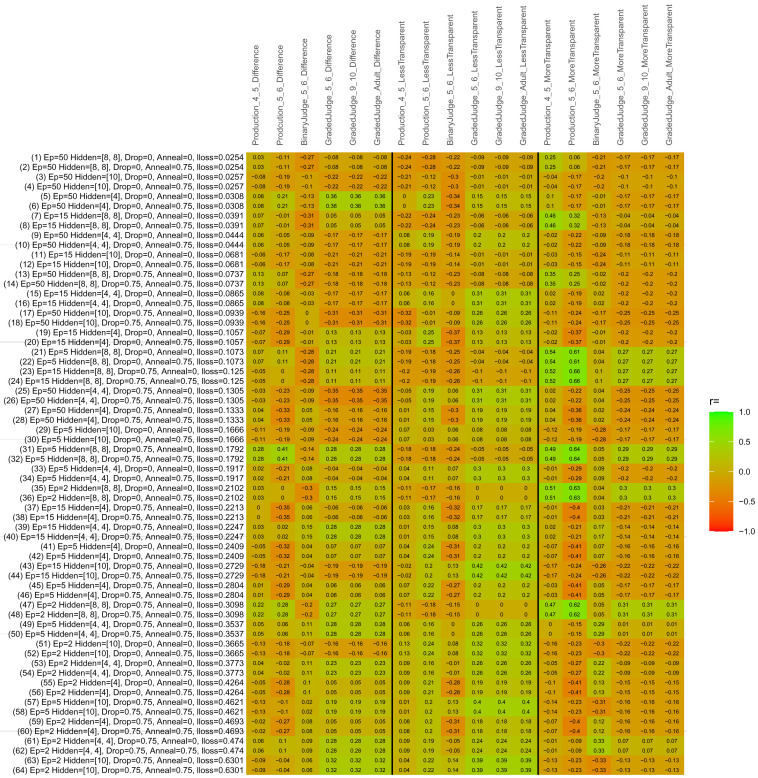
K’iche’: No-semantics-Model-human correlations (split-half test, adult-directed corpora).

**Figure 32.**
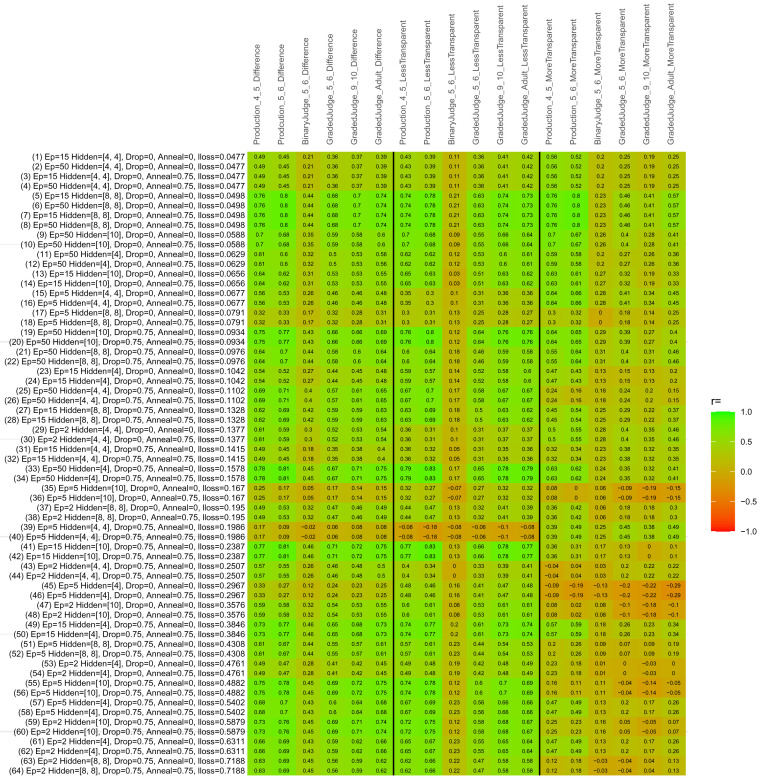
English: Model-human correlations (all verbs, child-directed corpora).

**Figure 33.**
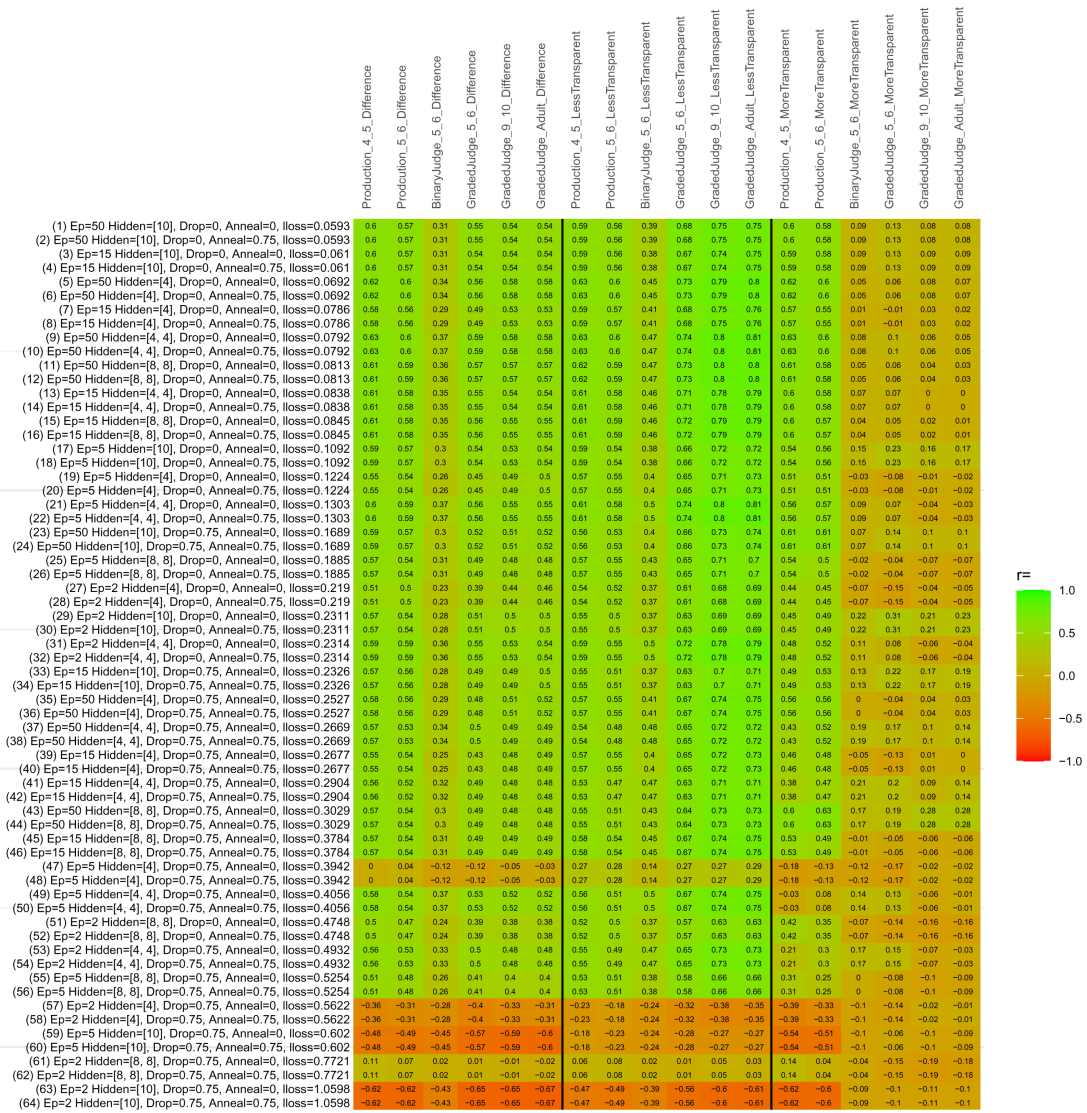
Hebrew: Model-human correlations (all verbs, child-directed corpora).

**Figure 34.**
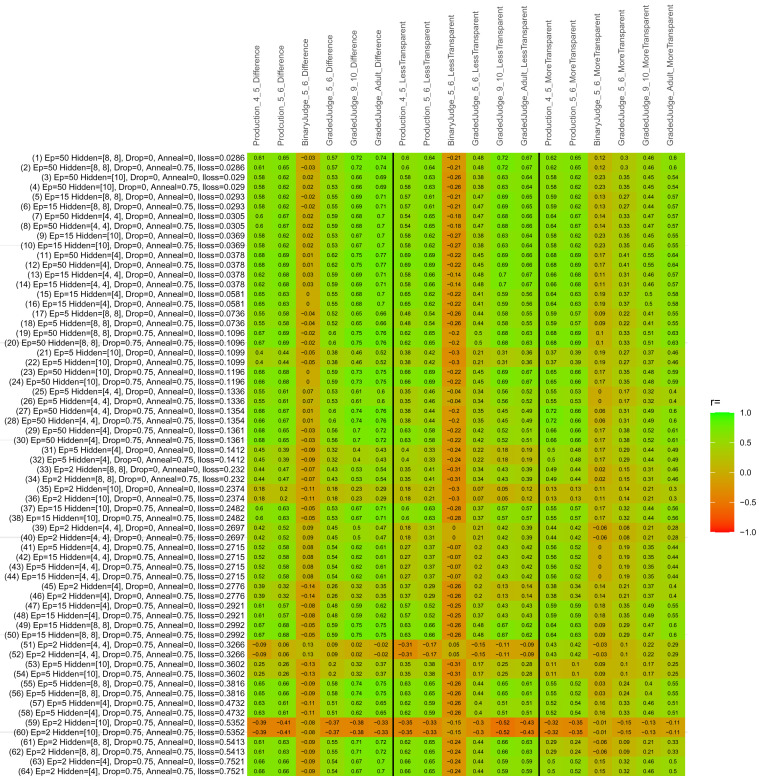
Japanese: Model-human correlations (all verbs, child-directed corpora).

**Figure 35.**
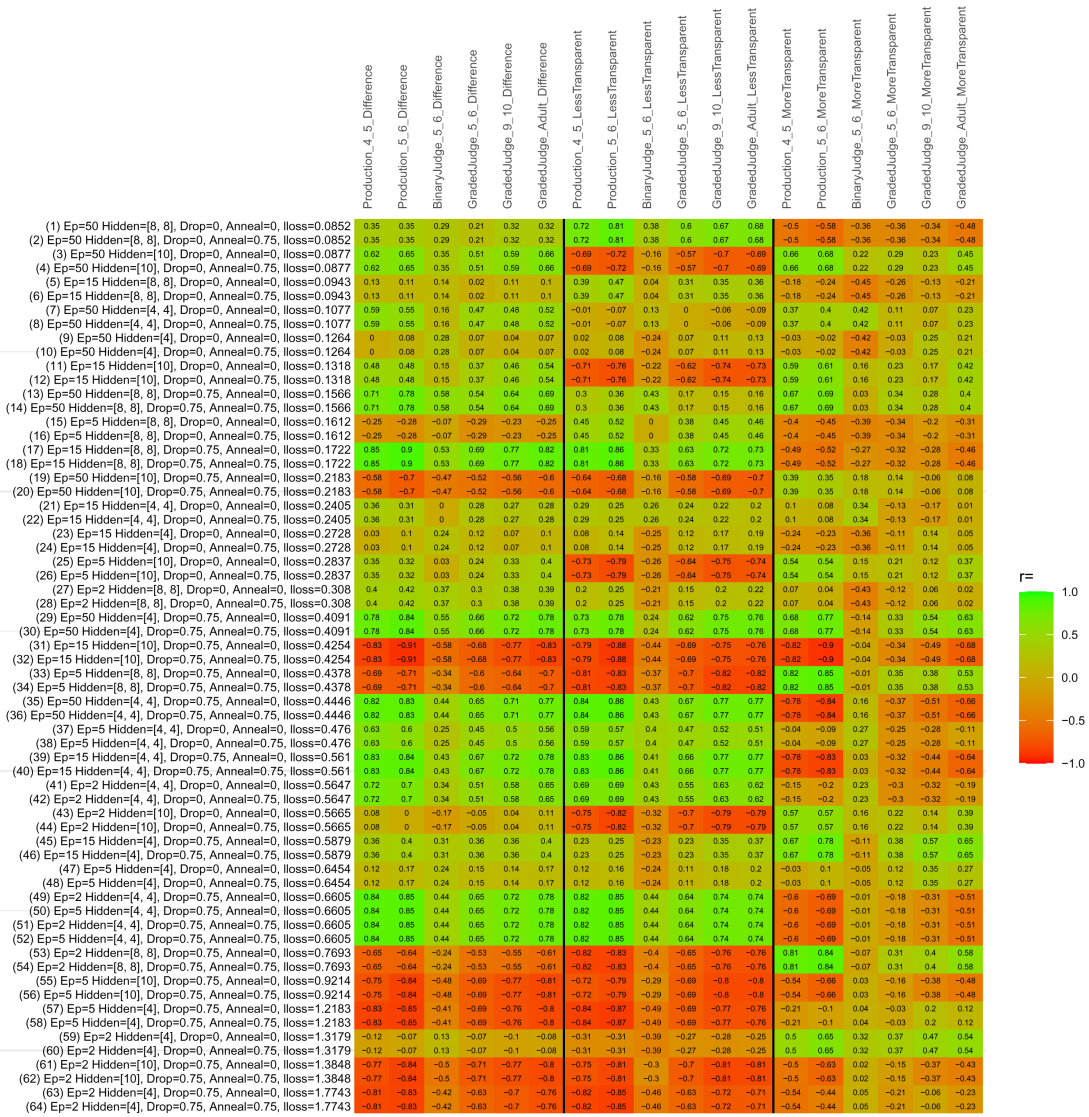
English: Model-human correlations (split-half test, child-directed corpora).

**Figure 36.**
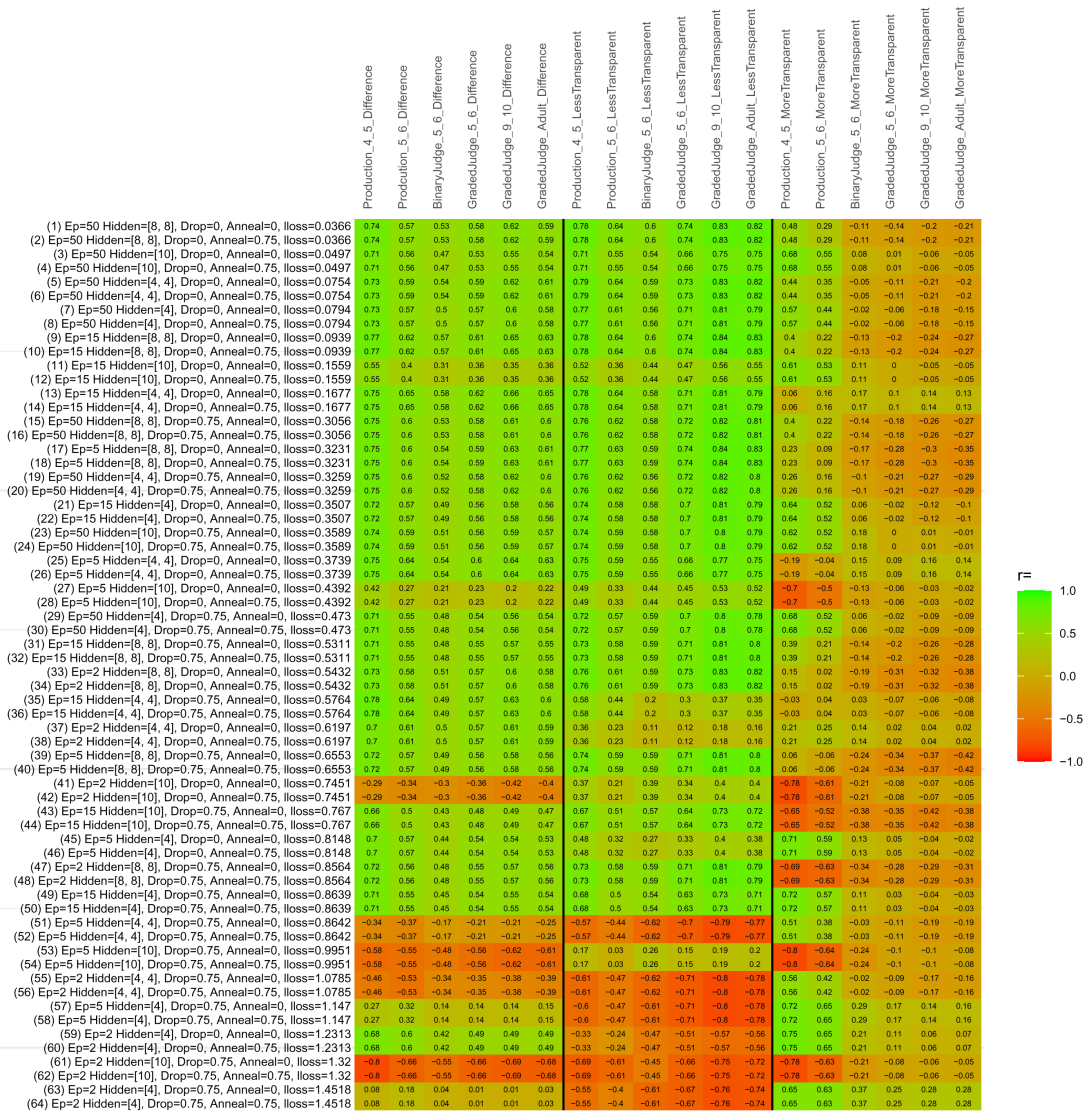
Hebrew: Model-human correlations (split-half test, child-directed corpora).

**Figure 37.**
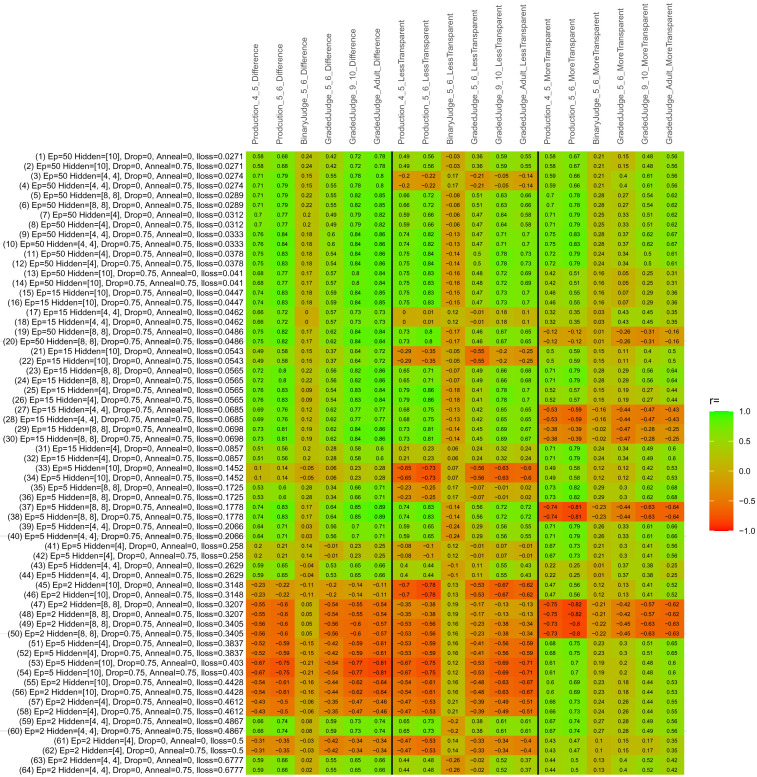
Japanese: Model-human correlations (split-half test, child-directed corpora).

**Figure 38.**
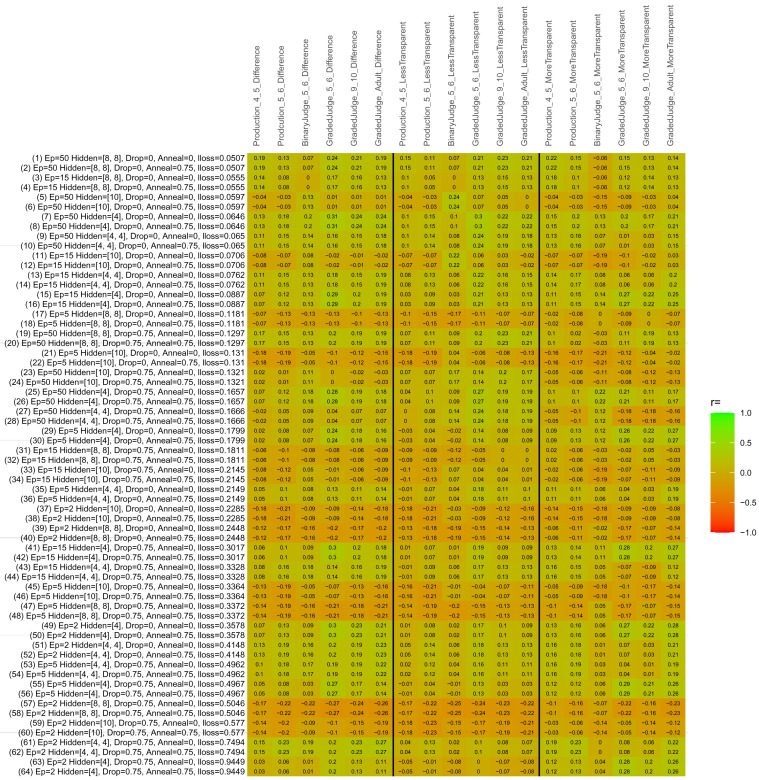
English: No-semantics-Model-human correlations (all verbs, child-directed corpora).

**Figure 39.**
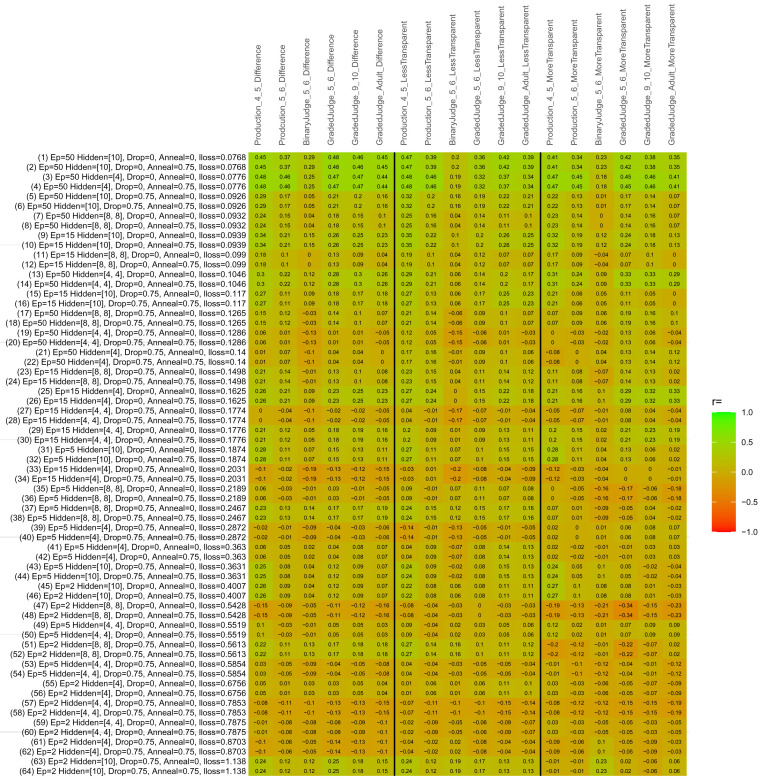
Hebrew: No-semantics-Model-human correlations (all verbs, child-directed corpora).

**Figure 40.**
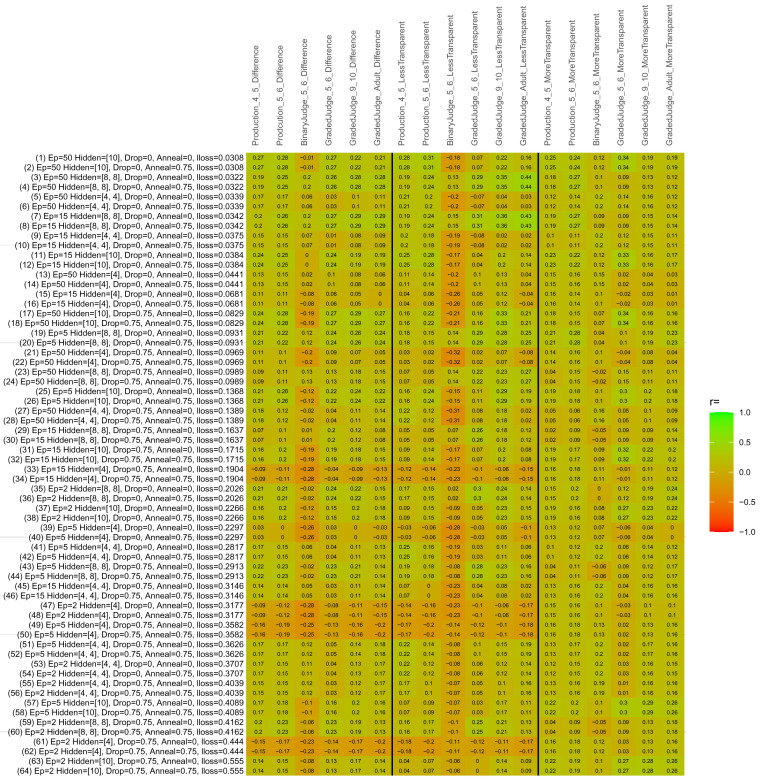
Japanese: No-semantics-Model-human correlations (all verbs, child-directed corpora).

**Figure 41.**
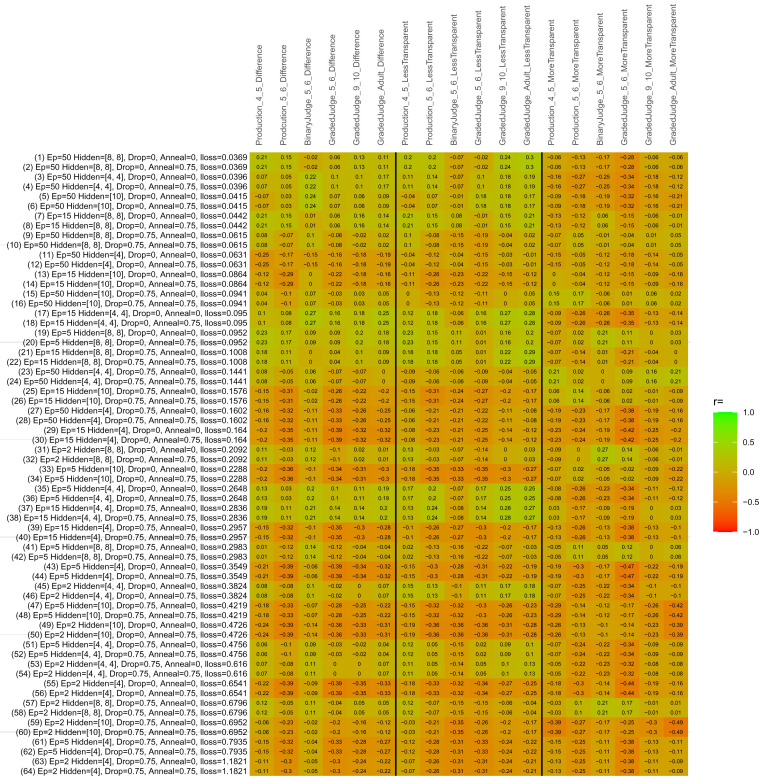
English: No-semantics-Model-human correlations (split-half test child-directed corpora).

**Figure 42.**
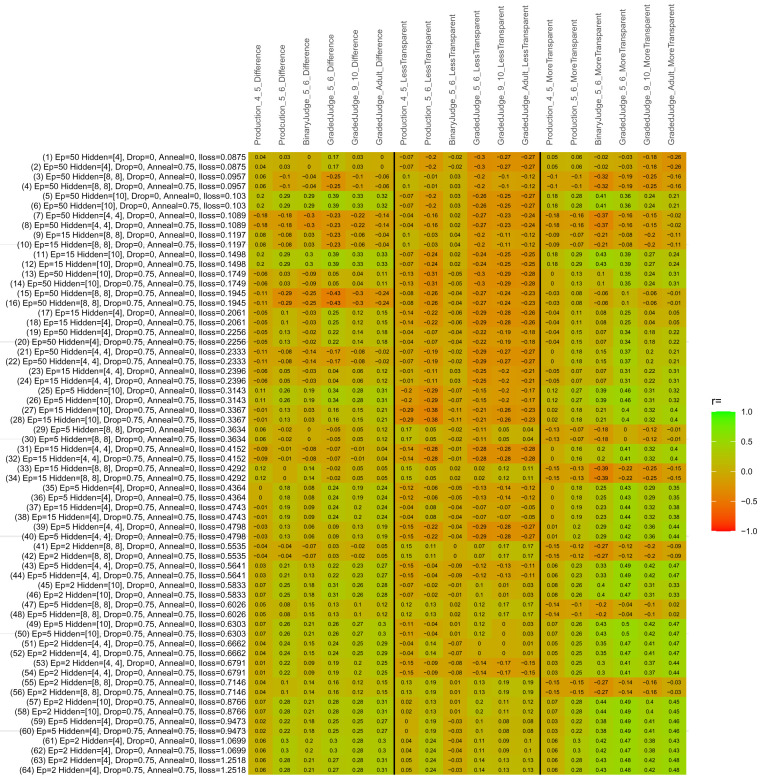
Hebrew: No-semantics-Model-human correlations (split-half test, child-directed corpora).

**Figure 43.**
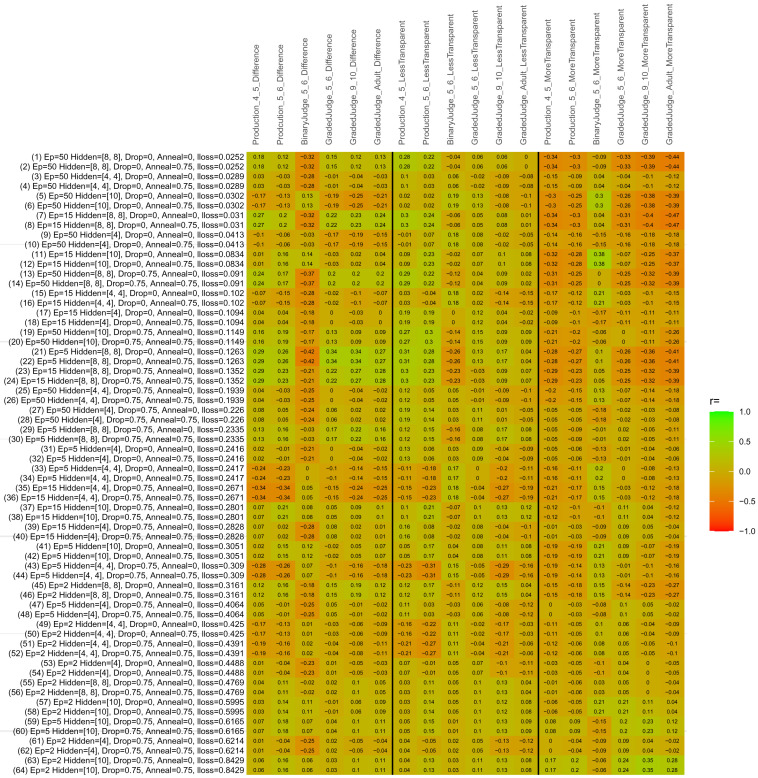
Japanese: No-semantics-Model-human correlations (split-half test child-directed corpora).

**Improved overall performance.** Overall, these more advanced models generally yield a better fit to the human data than do the simple two-layer models discussed above. That said, for the all-verb (c.f. split-half) models, the overall improvement is modest, and may result in part simply from the fact that so many different models were tried.

Better split-half performance. The general improvement is most noticeable for the split-half models which, for most languages, show only a small decrement compared to the all-verbs models. This is particularly the case for English (perhaps because the corpus data are so much more reliable), which showed essentially no decrement. For example, split-half English models showed correlations as high as *r*=0.92 and *r*=0.93 with 4–5 and 5–6-year olds’ production data (difference scores) versus *r*=0.83 and *r*=0.90 for the all-verbs (non-split half) models. Compared with the simpler two-layer models, then, these more advanced models do show better generalization. This suggests that human learners, too, are representing not just verbs and constructions (the input and output layers of the two-level models) but various intermediate-level abstractions (perhaps analogous to verb classes; Pinker, 1989).

**More hidden units equals better performance.** Although, in general, these more advanced models outperformed the previous two-layer models, some models with only a single hidden layer and/or only 4 hidden units per layer actually performed *worse* than the no-hidden-layer models (sometimes even yielding significant negative correlations with human data). This suggests that the intermediate abstractions that lie between verbs and constructions – for human learners as well as models – are complex and multifaceted (they are not, for example, simply four large verb classes). For most languages, best performance was achieved by models with two hidden layers each of 8 units, or a single hidden layer of 10 units.

**Binary judgment data are messy.** All models showed lower correlations with children’s binary grammaticality judgments (age 5–6) than with their production data (4–5 and 6–5) and with continuous grammaticality judgments (5–6, 9–10, adults). These findings suggest that – counter to theories that treat it as a binary construct – grammaticality is graded, and insisting on binary judgments is simply throwing away information.

**Verb semantics are crucial.** Removing verb semantics had a catastrophic effect on the model’s ability to generalize to unseen verbs in the split-half task, with most correlations close to zero and/or in the wrong direction. This is unsurprising, given that verb semantics is essentially the only valid basis on which the models could in principle generalize to unseen verbs. Absent semantics, the only meaningful “strategy” for the models is to over-predict the dominant response category (i.e., more-transparent or less-transparent causative form). More surprisingly, removing verb semantics also had a large deleterious effect for the all-verbs (i.e., non-split-half) models. This is surprising, as one would have expected the models to be able to learn verbs’ preferred causative constructions simply on a lexical-verb-by-verb basis. One possible explanation is that removing semantics, and hence forcing the models to do purely lexical learning, increases over-fitting of the corpus-based training set. That is, purely lexical models learn what individual verbs just so happen to do (in terms of their construction co-occurrences), in a particular corpus. Adding semantics allows models to learn what particular (semantic) types of verbs can do, smoothing out any verb-by-verb idiosyncrasies in the particular training corpus used.

**Dropout hinders, annealing is irrelevant.** We experimented with a rather high level of dropout (0.75), on the basis that children’s memory and processing limitations may effectively result in the loss of many learning trials. In fact, this high level of dropout (as compared to no dropout) only hurt the models, particularly for smaller models with lower numbers of epochs, particularly on the split-half test. We also experimented with a rather high level of annealing (0.75), on the basis that children’s learning slows over time. However, at least with these values (0 versus 0.75) we did not observe any relationship between annealing and model performance.

**Model performance is reassuringly consistent across architectures, human tasks and languages.** Overall, we ran 2,048 different models (98,304, counting the 48 different runs of each model). A concern, therefore, is that – if we were prepared to engage in some extreme cherry-picking – we could obtain almost any results we wanted. And perhaps by focussing on the best performing models for each language, we are overexaggerating the models’ overall performance. After all, if we generated 2,048 correlation coefficients purely at random, assuming a uniform distribution, we could expect more than 100 of at least r=0.93 (the highest observed). Reassuringly, therefore, model performance is generally consistent in three ways: (1) Given a particular human task to predict (e.g., 4–5 year-olds’ production data), most architectures within a given language show similar performance. For example, in the first column of Figure 12, the vast majority of correlations are in the region r=0.7–0.8. (2) Given a particular architecture and set of hyperparameter settings (e.g., Epochs=50 Hidden layers=[4, 4], Dropout=0, Annealing=0), the model shows comparable performance across all of the adult and child datasets (e.g., first row of Figure 12). (3) Given a particular architecture and set of hyperparameter settings the model shows comparable performance across languages (except for K’iche’, for which no model ever succeeds). For example, the architecture mentioned above (e.g., Epochs=50 Hidden layers=[4, 4], Dropout=0, Annealing=0) is one of the best performing across all languages.

**We failed to simulate children (including by reducing epochs or using child-directed speech).** Despite running over 2,000 different models, we failed to simulate child-like performance in this domain. That is, we found no combination of tasks, architectures and (hyper/)parameter settings where the model’s predictions correlated better with any of the child measures (production, binary/graded judgments) than with adults’ graded judgments. (The only possible contender is a handful of Hebrew split-half models – *r*=0.44 for 4–5-year-old production, *r*=0.25 for adult graded judgments, but the difference is small, and probably no more than a fluke). We certainly found plenty ways to break the models, by introducing capacity, memory or processing limitations that might be akin to those shown by children. But these broken models were equally broken when it came to predicting child and adult human data. Conversely, although we stumbled across some models that predicted children’s performance better than we could have dreamed (e.g., *r*=0.93 for English 5–6-year olds’ production data), these models provided a similarly excellent fit ( *r*=0.85) to adults’ graded judgment data. In particular, dramatically reducing the number of epochs to 2 or 5 (versus 15 or 50) to simulate lower levels of language exposure in young children certainly hurt model performance (particularly with high dropout and few hidden units/layers), but equally so for predicting child and adult data; it did not make the models more childlike. Similarly, basing the input sets on corpora of child-directed (versus adult-directed speech) hurt model performance (presumably because the relevant corpora are considerably smaller, and hence noisier), but equally so for predicting child and adult data. 

## General discussion

The question of how language learners (eventually) come to avoid the production of verb argument structure overgeneralization errors ( *The clown laughed the man) has long been seen as one that is both particularly central to acquisition research and particularly challenging ( Bowerman, 1988; Pinker, 1989). Focussing on causative overgeneralization errors of this type, Ambridge *et al.* (2020) built a computational model that learns, on the basis of corpus data and human-derived verb-semantic-feature ratings, to predict adults’ by-verb preferences for less- versus more-transparent causative forms (e.g, * *The clown laughed the man* vs *The clown made the man laugh*) across English, Hebrew, Hindi, Japanese and – to a lesser extent – K’iche. The aim of the present study was to investigate whether children learning these languages indeed produce such errors, and rate them as acceptable in a binary judgment task, and – if so – whether the computational model of Ambridge *et al.* (2020) can explain their patterning.

At one level, the answer to this question is a resounding “yes”. For example, English-speaking 4–5-year-olds produced errors like *Someone danced the boy and *Someone sang the boy (at rates of around 5% and 15% respectively), and the computational model of Ambridge *et al.* (2020) was able to predict their by-verb patterning with correlations in the region of *r*=0.75 (and *r*=0.5-0.6 for analogous errors in a binary judgment task). Similar results were observed across all languages (except K’iche’), and – with a few architectural tweaks – essentially the same underlying model was able to achieve correlations as high as *r*=0.8–0.9 with human judgment and production data, even when tested on unseen verbs. These correlations are all the more remarkable when it is born in mind that the model was trained only on semantics-augmented corpus data, and was never given access to the judgment or production data against which it was benchmarked.

At a developmental level, however, the answer is “no”. Despite the introduction of numerous limitations designed to mimic those facing child language learners, no model was able to simulate development, by providing a better fit to child than adult data; or to 4–5 than 5–6 year olds’ data. Thus, while the model offers an excellent mechanistic account of how learners (eventually) acquire verbs’ argument structure preferences and restrictions, it does not in fact explain why children make errors, or how they retreat from them. Why not?

One possibility is that the “retreat from overgeneralization” is largely accomplished by age 4–5; the youngest age-group in the present study. However, this does not seem likely, given that the relevant errors are attested amongst children aged 4 years and above in (a) the present study, (b) previous experimental studies ( Bidgood *et al.*, 2021; Fukuda & Fukuda, 2001), and (c) diary data ( Ambridge & Ambridge, 2020; Bowerman, 1988; Nakaishi, 2016).

A second possibility, and one that we have alluded to throughout, is that children’s underlying grammatical knowledge is essentially adultlike by this age, but children are more tolerant than adults of forms that deviate from that underlying grammar, in both judgments and production. If this is the case, then the solution to the retreat from overgeneralization would lie outside of the grammar; with – for example – increasing self-confidence that allows children to judge others’ utterances as unacceptable, or improvements in executive function that allow them to inhibit their own overgeneralizations.

A third possibility, discussed by Ambridge and Ambridge (2020), is that many of children’s “overgeneralization errors” are not in fact “errors” as such, but are well matched to the child’s communicative goals; indeed, better matched than the corresponding “grammatical” form would have been. The point is best made by some examples from Ambridge and Ambridge’s (2020: 126) diary study [notes added]:

- But what does Chloe [the diarized child] mean when she says, “Can you jump me off?”, “Jump me!”, “Jump me down (the slide)!”, “Jump me up there!”? She clearly does not mean ‘Do something that indirectly causes me to instigate an internally-caused jumping action’. She means ‘Pick me up and move me upwards’. The type of causation she has in mind is single-event, direct, external causation, of almost exactly the same type that is involved in breaking a cup. In short, she doesn’t mean ‘make me jump!’ [more-transparent causative], she means ‘jump me!’ [less-transparent causative]. (p.126)
- When Chloe says, “Mermaids have got special powers; they can die baddies”, she does not have in mind indirect, two-event causation [more-transparent causative; i.e., *make X die*], but direct, single-event causation [less-transparent causative]
- It is a similar story for *dance* (“I’m dancing it”, “I can dance it”, “Your turn to dance me, Dad”). The meaning is not ‘make me dance’ (e.g., by playing music) [more-transparent causative], but physically ‘dance me’ [less-transparent causative]. Likewise, for *eat* and *drink* (‘cause the food/liquid to go into my mouth’), *swim* (‘physically propel me through the water’), *reach* (‘lift me up’), *walk* (‘move my legs’), “go it faster”, “go[ing] them in”, *disappear* and *run*.

If this third possibility is correct, then the solution to the retreat from overgeneralization again lies outside the grammar: The reason adults don’t say things like “jump me”, “dance me” and “swim me” – and regard them as at least somewhat ungrammatical – is that adults generally do not enact single-event direct external causation on one another. And when they do these “ungrammatical” forms are allowed, or at least much improved ( Ambridge & Ambridge, 2020: 126–127):

As noted by, amongst others, Pinker (1989) the adult grammar allows transitivizations [i.e., less-transparent forms] that would otherwise be considered erroneous, when it is clear that the causation that the speaker has in mind is too direct to be properly conveyed by the [more-transparent] periphrastic causative; for example “when an advertisement for an amusement park says... *We’re gonna scream ya, and we’re gonna grin ya*” ( Pinker, 1989, p. 348). Similarly, although *disappear* is often discussed as a prototypical example of a verb that resists transitivization, it is not uncommon to read about dictators disappearing their enemies. While you can’t normally *walk* an adult, you can walk a dog and probably even a child (at least, you can *walk her to school*); and…a baseball pitcher can walk a batter.

Given, then, its impressive correlations with adult and non-developmental child data, perhaps the present model has taken us just about as far as we can go with solutions to the retreat from overgeneralization that are confined to “the grammar”. Perhaps to go the last mile, we will have to find solutions that lie *outside* the grammar, such as the speculative possibilities discussed above.

 A number of issues, however, do remain. First, despite its overall successes, the model did not significantly predict Japanese children’s binary grammaticality judgments or any of the K’iche’ data (for adults and children alike). While it is possible to come up with an apparently-reasonable explanation in each case, future work should investigate the alternative possibility that the computational model tested here perhaps does not apply universally. For Japanese binary judgments, the model’s failure is almost certainly due to a task effect, since the model does successfully predict both adults’ continuous judgments and children’s production data. For K’iche’ it is less clear. Although, as already noted, both the corpus and semantic-rating data are questionable, we should not discount the possibility that this model – and the account of causatives that it instantiates – is not well suited to languages like K’iche’ that have both transitivizing and intransitivizing morphological processes. For example, in English, Hebrew, Hindi and Japanese, *laugh* is perhaps the single most prototypical example of a highly intransitive verb that strongly prefers the less-direct, more transparent causative (e.g., Someone made the boy laugh > *Someone laughed the boy). Yet in K’iche’, intransitive *laugh* is derived from the transitive (though not transitive-causative) verb *laugh at*, and is – broadly speaking – acceptable in both causative forms; the same is true for *look* (derived from *look at*) and *speak* (from *speak about*). Perhaps, then, the crosslinguistic typology of causatives embodied by the computational model tested here is not quite accurate.

 This relates to a second issue: While it is certainly impressive that the model can account for adult and child data across – K’iche’ aside – four unrelated languages; these four languages hardly constitute a large or representative sample of all the languages of the world. Future work using the methods here should investigate whether this model generalizes to other languages.

 Third, future work using related methods should investigate whether an account of this type can explain speaker’s acquisition of verbs’ argument structure restrictions for a wide variety of syntactic and morphological constructions. We see no particular reason to believe that it cannot (e.g., see Ambridge & Blything, 2016; Li & MacWhinney, 1996, for similar models of the English *un-* prefixation and dative constructions), but, of course, the outcomes of such investigations cannot be anticipated.

Fourth, even for the restricted case of less- versus more-transparent causative forms, the model tested here does not solve the learning problem entirely, given that it starts from the point at which children have already acquired the relevant forms (e.g., the transitive-causative and *make* periphrastic causatives for English; lexical causatives and the – *(s)ase* causative marker for Japanese; the transitive and causative binyanim for Hebrew). Although the model learns a great deal about the meanings of these forms – i.e., the particular type of causation that is associated with each – the forms themselves are pre-given; and in most cases are highly abstract generalizations. In this respect, the account tested here is no different to all other accounts of this problem discussed in the Introduction. But until we have a model that can learn the generalizations in the first place, we cannot quite say that the problem of forming appropriately restricted generalizations has been solved.

 Finally, the present study has important methodological implications in that three different methods – continuous grammaticality judgments, binary grammaticality judgments and elicited production – have produced findings that are generally very highly correlated with one another. Indeed, we could – at a push – argue that five different methods have converged on similar conclusions, if we include both the diary data that first uncovered such errors (e.g., Bowerman, 1988; Table 1) and the corpus analysis used to derive the model’s training data. The methodological implications are – on the one hand – that triangulating different methods on the same set of stimuli provides a particularly detailed and robust test of a particular model; and – on the other – that where this is not possible, we can be reasonably confident that conclusions drawn on the basis of data collected using one method will generalize to another.

 In conclusion, while work remains to be done to extend this research to other constructions and other language families, the present findings that the computational model developed by Ambridge *et al.* (2020) explains both children’s binary grammaticality judgment and elicited production data across a range of languages suggest that a solution to the longstanding problem of learning verbs’ argument structure restrictions – and perhaps even the retreat from overgeneralization – is within our grasp.

## Data availability

### Underlying data

Open Science Framework: CLASS: Cross Linguistic Acquisition of Sentence Structure. https://doi.org/10.17605/OSF.IO/7F2DG ( Ambridge, 2021).

This project contains the following underlying data:

AAA_CLASS_R_Analyses (Zip file containing each of the following)

BinaryJudgmentsAndProduction (Folder containing each of the following)

BinaryModeling (Folder containing each of the following)

- Binary Correlations with Old Paper.r – R code for creating the figures that correlate the present binary judgment data with the adult continuous judgment data from Ambridge *et al.* (2020)
- Binary Modeling.R – R code for the computational modeling.
- BRM-emot-submit.csv – Valence norms from Warriner *et al.* (2013)
- ENG_Adults.csv – English grammaticality judgment data (from Ambridge *et al.*, 2020)
- ENG_Input.csv – English input file for the computational modeling
- ENG_Results.csv – English children’s binary judgment data – target for modeling
- English_Binary_Raw.csv – English children’s binary judgment data – raw data
- HEB_Adults.csv – Hebrew grammaticality judgment data (from Ambridge *et al.*, 2020)
- HEB_Input.csv – Hebrew input file for the computational modeling
- HEB_Results.csv – Hebrew children’s binary judgment data – target for modeling
- Hebrew_Binary_Raw.csv – Hebrew children’s binary judgment data – raw data
- HIN_Adults.csv – Hindi grammaticality judgment data (from Ambridge *et al.*, 2020)
- HIN_Input.csv – Hindi input file for the computational modeling
- HIN_Results.csv – Hindi children’s binary judgment data – target for modeling
- Hindi_Binary_Raw.csv – Hindi children’s binary judgment data – raw data
- JAP_Adults.csv – Japanese grammaticality judgment data (from Ambridge *et al.*, 2020)
- JAP _Input.csv – Japanese input file for the computational modeling
- JAP _Results.csv – Japanese children’s binary judgment data – target for modeling
- Japanese_Binary_Raw.csv – Japanese children’s binary judgment data – raw data
- KIC_Adults.csv – Kiche’ grammaticality judgment data (from Ambridge *et al.*, 2020)
- KIC_Input.csv – Kiche’ input file for the computational modeling
- KIC_Results.csv – Kiche’ children’s binary judgment data – target for modeling
- Kiche_Binary_Raw.csv – K’iche’ children’s binary judgment data – raw data
- Tables3-4.csv – Table 3– Table 4 from the present article
- Table5.csv – Table 5 from the present article
- XX_Not_Included_OriginalModelArchitecture.pdf – Figure showing the architecture of the original computational model (no longer included in the paper).

ProductionModeling (Folder containing each of the following)

- BRM-emot-submit.csv – Valence norms from Warriner *et al.* (2013)
- ENG_4_5 – English 4-5-year-olds’ raw production data
- ENG_5_6 – English 5-6-year-olds’ raw production data
- ENG_Adults – English adults’ judgment data (from Ambridge *et al.*, 2020)
- ENG_Input.csv – English input file for the computational modeling
- ENG_Results.csv – English children’s production data – target for modeling
- HEB_4_5 – Hebrew 4-5-year-olds’ raw production data
- HEB_5_6 – Hebrew 5-6-year-olds’ raw production data
- HEB_Adults – Hebrew adults’ judgment data (from Ambridge *et al.*, 2020)
- HEB_Input.csv – Hebrew input file for the computational modeling
- HEB_Results.csv – Hebrew children’s production t data – target for modeling
- HIN_4_5 – Hindi 4-5-year-olds’ raw production data
- HIN_5_6 – Hindi 5-6-year-olds’ raw production data
- HIN_Adults – Hindi adults’ judgment data (from Ambridge *et al.*, 2020)
- HIN_Input.csv – Hindi input file for the computational modeling
- HIN_Results.csv – Hindi children’s production data – target for modeling
- JAP_4_5 – Japanese 4-5-year-olds’ raw production data
- JAP_5_6 – Japanese 5-6-year-olds’ raw production data
- JAP_Adults – Japanese adults’ judgment data (from Ambridge *et al.*, 2020)
- JAP _Input.csv – Japanese input file for the computational modeling
- JAP _Results.csv – Japanese children’s production data – target for modeling
- KIC_4_5 – K’iche’ 4-5-year-olds’ raw production data
- KIC_5_6 – K’iche’ 5-6-year-olds’ raw production data
- KIC_Adults – K’iche’ adults’ judgment data (from Ambridge *et al.*, 2020)
- KIC_Input.csv – Kiche’ input file for the computational modeling
- KIC_Results.csv – Kiche’ children’s production data – target for modeling
- Table8.csv – Table 8 from the present article
- Tables6-7.csv – Table 6– Table 7 from the present article
- V5_Production_Modeling_Environment.RData – R Environment file for original computational modeling
- Production Correlations with Old Paper.R – R code for creating the figures that correlate the present binary judgment data with the adult continuous judgment data from Ambridge *et al.* (2020)
- V5_Production_Modeling.R – R code for original computational modeling

Figures (Folder containing Figure 1– Figure 43 from the present article)

ORE_Version3 (Folder containing each of the following, relating to Study 3: Further Computational Modeling; specifically the models trained on adult-directed corpora)

- English_Binary_Judge.csv – English binary judgment data from the present study (target for modeling)
- English_Cognition.csv – English adult judgment data from Ambridge *et al.* (2020) (target for modeling)
- English_Input.csv – English Input to the computational model
- English_Production.csv – English production data from the present study (target for modeling)
- Hebrew_Binary_Judge.csv – Hebrew binary judgment data from the present study (target for modeling)
- Hebrew_Cognition.csv – Hebrew adult judgment data from Ambridge *et al.* (2020) (target for modeling)
- Hebrew_Input.csv – Hebrew Input to the computational model
- Hebrew_Production.csv – Hebrew production data from the present study (target for modeling)
- Hindi_Binary_Judge.csv – Hindi binary judgment data from the present study (target for modeling)
- Hindi_Cognition.csv – Hindi adult judgment data from Ambridge *et al.* (2020) (target for modeling)
- Hindi_Input.csv – Hindi Input to the computational model
- Hindi_Production.csv – Hindi production data from the present study (target for modeling)
- Japanese_Binary_Judge.csv – Japanese binary judgment data from the present study (target for modeling)
- Japanese_Cognition.csv – Japanese adult judgment data from Ambridge *et al.* (2020) (target for modeling)
- Japanese_Input.csv – Japanese Input to the computational model
- Japanese_Production.csv – Japanese production data from the present study (target for modeling)
- Kiche_Binary_Judge.csv – K’iche’ binary judgment data from the present study (target for modeling)
- Kiche_Cognition.csv – K’iche’ adult judgment data from Ambridge *et al.* (2020) (target for modeling)
- Kiche_Input.csv – K’iche’ Input to the computational model
- Kiche_Production.csv – K’iche’ production data from the present study (target for modeling)
- V12_Deep_Learning.R – R code to run the computational models
- V12_Just_Heatmaps.R – R code to create Figure 12– Figure 31

ORE_Kids (Folder containing each of the following, relating to Study 3: Further Computational Modeling; specifically the models trained on child-directed corpora)

- English_Binary_Judge.csv – English binary judgment data from the present study (target for modeling)
- English_Cognition.csv – English adult judgment data from Ambridge *et al.* (2020) (target for modeling)
- English_Input.csv – English Input to the computational model
- English_Production.csv – English production data from the present study (target for modeling)
- Hebrew_Binary_Judge.csv – Hebrew binary judgment data from the present study (target for modeling)
- Hebrew_Cognition.csv – Hebrew adult judgment data from Ambridge *et al.* (2020) (target for modeling)
- Hebrew_Input.csv – Hebrew Input to the computational model
- Hebrew_Production.csv – Hebrew production data from the present study (target for modeling)
- Japanese_Binary_Judge.csv – Japanese binary judgment data from the present study (target for modeling)
- Japanese_Cognition.csv – Japanese adult judgment data from Ambridge *et al.* (2020) (target for modeling)
- Japanese_Input.csv – Japanese Input to the computational model
- Japanese_Production.csv – Japanese production data from the present study (target for modeling)
- V12_Deep_Learning.R – R code to run the computational models
- V12_Just_Heatmaps.R – R code to create Figure 32– Figure 43

### Extended data

Open Science Framework: CLASS: Cross Linguistic Acquisition of Sentence Structure. DOI 10.17605/OSF.IO/7F2DG ( Ambridge, 2021).

This project contains the following extended data:

- AAFinal_Sentence_Stimli(Version 2).xlsx (Final sentence stimuli)
- **Binary grammaticality instructions1.docx (Full text of instructions given to children completing the binary judgment task)**
- Binary Judgement.zip (Zip file containing all video and audio stimuli, blank participant record and key sheets, and the sticker grid completed by children)
- Binary Judgement procedure.mp4 (Video illustrating the binary judgment procedure)
- **Practice animations.zip (Folder containing practice animations for the judgment warm up)**
- **Child instructions production.docx (Full text of instructions given to children completing the production task)**
- CausativeAnimations.zip (Zip file containing all video and audio stimuli for the production task)
- EnglishJudgmentsPsychoPy.zip (Zip file containing the PsychoPy experiment to run the judgment task).
- JudgmentLists.zip (Zip file containing the different counterbalance lists for each language)
- Production procedure.mp4 (Video illustrating the elicited production procedure)
- Prereg Production and Binary Judgments.pdf (Preregistration of the methods used)

Data are available under the terms of the Creative Commons Zero "No rights reserved" data waiver (CC0 1.0 Public domain dedication).

## Footnotes

^1^The periphrastic causative form is termed the more-transparent form because it includes an overt causative marker ( *make*). More- and less-transparent causative forms for Hebrew, Hindi, Japanese and K’iche’ are set out in the Methods section.
