# Supplementary material for: Testing a computational model of causative overgeneralizations: Child judgment and production data from English, Hebrew, Hindi, Japanese and K’iche’
Source: Open Res Eur. 2022 Jan 12;1:1. Originally published 2021 Mar 24. [Version 2] doi: 10.12688/openreseurope.13008.2 (PMC10446094; doi:10.12688/openreseurope.13008.2)
Supplement: Peer review history

# Peer review: Reviewer response for version 2

**DOI:** 10.21956/openreseurope.15546.r28344

**Published:** 2022-02-14

**Authors:** Vsevolod Kapatsinski

**Affiliations:** 1 Department of Linguistics, University of Oregon, Eugene, OR, USA

This revision has admirably addressed my concerns. I appreciate the careful exploration of the space of model parameters that might account for retreat from overgeneralization.

 My one concern is that the authors might now be overly pessimistic about the ability of their model to capture the developmental change because a linear correlation with binarized/categorical data will always be lower than with an underlying continuous variable. Consider the following simulation:

 nReps<-1000

 nKids=32

 cor.sample.continuous<-rep(0,nReps)

 cor.sample.binary<-rep(0,nReps)

 for (i in 1:nReps)

 {

   prediction<-rnorm(mean=0,sd=1,n=nKids)

   rating<-prediction*.7+rnorm(mean=0,sd=.3,n=nKids)

   cor.sample.continuous[i]<-cor(prediction,rating)

   binary.rating<-rep(0,nKids)

   binary.rating[which(rating>0)]<-1

   cor.sample.binary[i]<-cor(prediction,binary.rating)

 }

 length(which(cor.sample.continuous>cor.sample.binary)) #continuous correlation always higher than binary in my run

 hist(cor.sample.continuous-cor.sample.binary)

 The authors note that binary judgments are messy compared to production (something we have also consistently observed in my lab). However, even production data are categorical: the speaker has to make a choice of what to produce, and if they maximize they will always choose the more acceptable option, even if the acceptability difference is small. So I think the loss of information that comes from turning continuous variables categorical might still explain the advantage of the model on the continuous adult data here.

 I would have also liked to see graphs that show the points that follow Figure 43 (showing model fit as a function of these parameters and maybe even a confidence region for a non-parametric regression thereof), and relegated Figs 12-43 to an appendix. It is rather hard to extract the patterns or evaluate their reliability across the huge number of tables.

If applicable, is the statistical analysis and its interpretation appropriate?

Partly

Is the study design appropriate and is the work technically sound?

Partly

Is the work clearly and accurately presented and does it engage with the current literature?

Yes

Are the conclusions drawn adequately supported by the results?

Partly

Are sufficient details of methods and analysis provided to allow replication by others?

Partly

Are all the source data and materials underlying the results available?

Yes

Reviewer Expertise:

My research deals with the role of domain-general learning mechanisms in language acquisition.

I confirm that I have read this submission and believe that I have an appropriate level of expertise to confirm that it is of an acceptable scientific standard.

# Peer review: Reviewer response for version 2

**DOI:** 10.21956/openreseurope.15546.r28345

**Published:** 2022-01-25

**Authors:** Lisa Pearl

**Affiliations:** 1 Language Science, University of California, Irvine, Irvine, CA, USA

I like to sign my reviews when possible.  ~~Lisa S. Pearl

 I think this revision has done a fine job addressing the comments that occurred to me during my reading of the previous manuscript version. I also really appreciate the motivation and implementation of Study 3, which I mention in the more-detailed comments below. Because the revision did such a fine job addressing my previous comments, I was then able to notice things in this current version that I don’t believe I noticed before (summarized below). All my current comments are basically suggestions for potential clarifications, especially a conceptual one involving what counts as being “part of the grammar” vs. not. I definitely support the indexing of this manuscript, though I do think it would be improved by incorporating some text that responds to the comments I raise below.

 Specific comments:

1.  
**What’s part of the grammar**
  When we start looking at results from the behavioral work (e.g., in Study 1), it’s the “difference” scores (the relative rate of less- vs. more-transparent forms) that we get the best results from. Given this, it may be helpful to talk about the adult-like “knowledge” children seem to have as the rate at which one form is preferred over another, given what Figure 3 is plotting. This finding is contrasted with children’s individual judgments of less- or more-transparent forms, which may “deviate from the underlying grammar”. So, here, “grammar” is about the relative rate on a verb-by-verb basis, rather than the acceptability of any particular form. 
  To play devil’s advocate, I could imagine someone thinking that the underlying grammar would also include the knowledge of individual verb form’s acceptability (e.g., that “Someone broke it” and “Someone made it break” are both pretty acceptable, even if one is used more than another). This issue of what’s part of the grammar vs. not comes up again when you discuss why children seem to over-accept forms that adults find less acceptable – you suggest one explanation is simply metacognition immaturity (i.e., something like developing process abilities, rather than developing knowledge), because children’s “grammatical knowledge” is essentially adultlike. "Adult-like" is true for the rate of form use, but not obviously so for the acceptability of a particular form for a specific verb.
  In a related vein, in the general discussion, you mention the possibility that children are sensitive to the internal-causation vs. external-causation meanings associated with the more- and less-transparent forms (which I love!), and you talk about this sensitivity as something outside the grammar. Playing devil’s advocate again, is it obviously outside the grammar? 
  That is, isn’t it still something about the language (and so about the language’s grammar) to learn which precise semantic meanings are allowed to be implemented in the language with the appropriate syntactic forms? The learnability issue then reminds me of the problem in lexical acquisition, where children need to learn which concepts are lexicalized with single words in their language.
  More generally, this issue of being part of (i.e., inside) vs. outside the grammar may be something that gets clarified by an earlier explicit discussion of what’s considered “grammar” vs. not, and why (e.g., the rate of less-transparent vs. more-transparent forms vs. a specific form for a specific verb being more vs. less acceptable). If what’s considered “grammar” is clarified there, it may be easier to refer back to whatever distinction in the general discussion when you delve into the third possible explanation about children overgeneralizing with respect to the mapping between causation semantics and causative forms.
2. **Introduction, the verb-semantics hypothesis**
  It seems that the verb-semantics hypothesis isn’t really a learning mechanism *per se*, as described, in contrast to the other two statistical approaches. Instead, verb semantics seems more like a feature that would be used in a learning mechanism – for instance, the mechanism would be that the learner recognizes the relevant semantic feature and then learns to map its various values (internal-causation vs. external-causation) to the appropriate forms (i.e., internal causation = more-transparent periphrastic vs. external-causation = less-transparent transitive in English). It may be useful to briefly note this when you introduce these three approaches, especially because your modeling work will use the semantic feature approach and embed it in a specific type of statistical learning mechanism via the computational model.
3. **Introduction, discussion of previous Ambridge et al. 2020 model**
  It may be helpful to explicitly connect the implementation of this model to the mechanisms you just introduced from prior approaches (i.e., preemption, entrenchment, verb semantics). In particular, the learning based on relevant input frequencies captures preemption, since preemption depends on relative input frequencies; having an “OTHER” node for utterances that don’t use either of the causative forms relates to entrenchment, since the total frequency of the verb usage (across all forms) matters; the input contains four “semantics” units, which captures the verb-semantics hypothesis.
4. **Study 1 results, where valence for Japanese and Ki’che’ matters more, in particular that the more-transparent form is preferred when there’s negative valence because the more-transparent form suggests unintentionally. **
  To me, this seems like a really interesting finding about the (potential) relationship between (un)intentionality, causative form, and valence. I wonder if this is something that might somehow be incorporated into future modeling work – that is, that the forms themselves (more- vs. less-transparent) would be associated with specific semantic features, rather than only the verbs being associated with semantic features. The idea that forms could be associated with semantic features comes back again when we think about the distinction of internal-causation vs. external-causation.
5. **Study 3**
  I think Study 3 is a really valuable contribution, with the goal of building processing limitations into the computational model, in order to try to capture some of the actual learning effects (i.e., what would cause the retreat from overgeneralization, if it actually hasn’t already occurred by age four). More generally, even if the over-acceptance of individual forms (either the less-transparent or the more-transparent) is due to some kind of immature processing, rather than immature knowledge of what’s acceptable, it seems like that could be captured in a model that also has immature processing, implemented in whatever way makes the most sense. And then, we would have a better idea about potential explanations for what the immature processing actually is in children that generates the observed behavior in the experiments (i.e., related to the immature processing implementations in the model vs. something else, like the executive function maturation suggested at various points).
6. **The model in Study 3**
  It might be helpful to note which option in these model parameter explorations you consider to be more child-like/immature processing  – you do this for dropout, but you can probably just hammer it home in each case.  For instance, in the case of the architecture, would it be having 1 hidden layer, since that represents a cognitive bottleneck (forcing more generalization)? (It may also help to mention some interpretation of what hidden layers could represent, as you do in your results discussion: they correspond to intermediate representations above the individual verb level.) For dropout, the 0.75 dropout is more child-like/immature; for annealing, .75 = starting out by exploring lots of possibilities – even less probable ones – and then becoming more conservative over time is (which means exploring only the more probable possibilities); this is more child-like/immature because there’s actual maturation of the “conservativity” in exploration. For the epochs, having fewer epochs means less data exposure, which is more child-like/immature. In the case of the epoch limitation, you may want to note that this is less a processing limitation, and instead a simple experience (input quantity) limitation.
7. **Organizational suggestion for results of study 3**
  It’s brilliant to have all the results available in Tables 12-43, but as you note in the text itself, the information is a little overwhelming, and can be summarized pretty simply. Maybe it’s better to have these in an appendix instead, so as not to break up the flow of the text so much?
8. **Study 3 interpretation and limitation for model exploration with respect to dropout and annealing**
  It might be helpful to mention that you only looked at one value for each of these (0.75 = severe dropoff and major annealing), and of course other values might have different effects. But this is for future work, if we believe that these parameters capture useful potential aspects of immature processing (in particular, immature memory/attention for dropout and immature “conservativity” for annealing).
9. **General discussion, executive function as potential future modeling work**
  I wonder if the executive function aspect could be incorporated into a future computational model. The way the process is described here, it would be a two-step model: step 1 is the learning process implemented in the current model to produce the probability of an output form for a given verb, and step 2 is an additional layer that controls actual output (how much inhibition there is for a generalization). If something like this is what you have in mind, it could be worth mentioning as a concrete future work possibility.

If applicable, is the statistical analysis and its interpretation appropriate?

Yes

Is the study design appropriate and is the work technically sound?

Yes

Is the work clearly and accurately presented and does it engage with the current literature?

Partly

Are the conclusions drawn adequately supported by the results?

Partly

Are sufficient details of methods and analysis provided to allow replication by others?

Partly

Are all the source data and materials underlying the results available?

Yes

Reviewer Expertise:

Computational cognitive models of language development.

I confirm that I have read this submission and believe that I have an appropriate level of expertise to confirm that it is of an acceptable scientific standard.

# Peer review: Reviewer response for version 1

**DOI:** 10.21956/openreseurope.14078.r26696

**Published:** 2021-05-14

**Authors:** Lisa Pearl

**Affiliations:** 1 Language Science, University of California, Irvine, Irvine, CA, USA

I like to sign my reviews when possible. ~~Lisa S. Pearl

 I think this manuscript makes great contributions in terms of addressing an interesting theoretical question about language acquisition: why children overgeneralize certain verb forms and how they recover from that overgeneralization. Moreover, the authors use multiple methods and look at data from multiple unrelated languages to assess the generalizability of their conclusions, and are pretty clear about the limitations of their current findings. As a computational cognitive modeler focusing on language acquisition, I especially appreciate the difficulties that go into (i) designing a computational model that connects concretely to empirical acquisition data, and (ii) interpreting model results in a cognitively-meaningful way. Because of this, I found myself deeply interested in what I would consider relevant modeling details, and unfortunately a bit confused by some of the main takeaways without those details. If possible, I’d love to see a revision that included some of that information, focusing on the aspects I mention in more detail below. Besides this, I had a few other specific thoughts that I discuss below.

**(1) The model**

*(a) Model input, especially for children of different ages*

 It seems important to use input data distributions from children of the ages intended to be modeled (4;0-5;0, 5;6-6;6), as it’s possible that input frequencies (particularly for uses of individual lexical items) would shift as children get older. Relatedly, children’s perception of the relevant semantic properties may also be developing over time -- right now, adult data are used as a standin because that’s the empirical data available (which is really great to have!). But that, along with the changing input frequencies, might explain why the model doesn’t capture younger (immature) child behavior.

 More generally, to capture children’s knowledge at different ages with the kind of incremental model you have, I think you’d want to start the model off with some base level of knowledge (the equivalent of an informed Bayesian prior) that corresponds to what a child of that age is meant to know. For instance, if you wanted to pursue this idea, I could imagine running the model for the 4;0-5;0 child data, and then using that end state of that model as the start state for running the 5;6-6;6 child data (assuming the input frequency data actually differed between these two groups).

 I don’t think the current manuscript needs to do this, but I think it’s worth discussing as a potential limitation and/or future work.

*(b) Model implementation*

 (i) The manuscript notes that the model used is the very same oneimplemented by Ambridge *et al*. 2020, but it would be helpful just to give a very basic sketch of some of the finer details when it’s first presented (e.g., how many input units are there, where the input data come from, etc.)

 More generally, given the explanatory goals you have with the input features you’re using, I’m curious about them motivation for using a neural network, rather than a more transparent classifier (like SVM, logistic regression) or cognitive model (like Bayesian inference). It seems like a more-transparent modeling methodology would speak to the goal of how predictive/explanatory the hypothesized features are. Of course, I realize you’ve already developed the model in the Ambridge *et al*. 2020 previous work, and want to test it here. But I do wonder if the explanatory goal would have been better-served by a different modeling choice. Perhaps the model incorporates information in a distributed way (e.g., like current word embedding approaches like GloVe or RoBERTa do), and these input representations would be hard to replicate in a different modeling type? At any rate, I do think a little more background on the modeling choice might be helpful somewhere in this paper for readers like me.

 (ii) Split-half validation: I think it’s good to see the split-half validation, even if you couldn’t do it for K’iche’ in Study 2. The results from split-half validation are much more believable, as opposed to the test-on-training when you use the full 60. That is, the split-validation captures what the model has learned more generally rather than what the model has learned (and potentially overfit) for these data. I know the full-60 correlations have higher values, but the split-half validation is more credible for the interesting explanatory claims you want to make about input frequency and the semantic features. Because of this, I’d be careful about playing up the full-60 modeling results compared with the split-half validation.

 (iii) The discussion currently says about the modeling approach: “Given that an identical model can predict...without having been trained on any of these datasets”. I think I may have misunderstood something fundamental about the model then (and this may make my previous comments about the full-60 vs. split-half validation make less sense). I thought the model, as a neural net, gets trained to predict the correct output value on the basis of the input, and is given pairs of input-output to learn from over time. So, because the model has seen all 60 verbs (for the full-60 models) or 30 (for the split-half validation models), the model has in fact been trained (for the full-60) or partially trained (for the split-half) on these datasets. 

 If this isn’t right, then I think the manuscript would definitely benefit from more description of the model itself and how it was trained, since this seems like a really important aspect of what you want to say in the general discussion. To me, the key idea is that certain aspects of the input have great explanatory power (with r around 0.5): the input frequencies of the forms, and the four semantic features. With these viewed as the relevant part of the input, a modeled learner can both overgeneralize and retreat from overgeneralization.

*(c) What the model is meant to do: *Related to the previous point, I think it may be helpful to draw out the explanatory power more of the frequency and semantic factors you investigate. If I’m understanding the goal of the modeling correctly, that’s really what the model is meant to do: predict when overgeneralization does and doesn’t happen, on the basis of these factors.

**(2) What counts as overgeneralization**

 (i) When I was looking at Figure 3, I had a minor point of confusion about what counts as an overgeneralization. Overgeneralizations are defined as items where kids use the forms more equally than adults (i.e., kids think the forms are less different than adults). But, why not focus on the more natural overgeneralizations for the causative, where adults prefer the more-transparent (“A made B VERB”) over the less-transparent (“A VERBed B”) form moreso than children do?

 In Hindi for example, it seems like overgeneralizations also include items where adults prefer the less-transparent (“A VERBed B”) over the more-transparent, because the adult difference score is positive.

 (ii) Study 2: 

 The current manuscript suggests that less-transparent forms (“A VERBed B”) are more frequent in children’s input, and that’s why children use the less-transparent forms more than adults. Is the input difference in general for causative verbs, or just for individual verbs? That is, is the less-transparent more common for causative verbs as a whole, and that’s what you think is causing the overgeneralization here? If so, then it means children have grouped together “causative verbs” as a class, and are tracking frequencies about that class. 

 If instead you mean that less-transparent forms are more common for individual verbs, then do you mean that “overgeneralization” (as defined here by using the less-transparent form more often than adults) is simply driven by the input? That is, it’s just a reflection of an input that supports overgeneralizations, when defined as using the less-transparent form more often than you should. (Side note: This seems a little different from allowing a form that adults categorically think is not allowed. It might be helpful to note this.)

 Followup if you meant children’s input has more less-transparent uses for individual verbs: Is this something you could test for explicitly, by just seeing how well input frequency accounts for child judgments, and not including the 4 semantic features?

**(3) Very minor thing: Figure resolution**

 Several figures in my pdf of the manuscript were rather fuzzy -- figures 1-3, an 9-11. It would be good to get better resolution versions of these.

If applicable, is the statistical analysis and its interpretation appropriate?

Yes

Is the study design appropriate and is the work technically sound?

Yes

Is the work clearly and accurately presented and does it engage with the current literature?

Partly

Are the conclusions drawn adequately supported by the results?

Partly

Are sufficient details of methods and analysis provided to allow replication by others?

Partly

Are all the source data and materials underlying the results available?

Yes

Reviewer Expertise:

Computational cognitive models of language development.

I confirm that I have read this submission and believe that I have an appropriate level of expertise to confirm that it is of an acceptable scientific standard, however I have significant reservations, as outlined above.

# Response

**Published:** 2022-01-07

**Authors:** Ben Ambridge

**Affiliations:** University of Liverpool, UK

We thank this reviewer for their very helpful comments. In particular, the prompt to investigate the extent to which the model can explain the *retreat* from overgeneralization developmentally inspired new modeling work that caused us to rethink exactly what the model is telling us. Reviewer comments in italics.

*I think this manuscript makes great contributions in terms of addressing an interesting theoretical question about language acquisition: why children overgeneralize certain verb forms and how they recover from that overgeneralization. Moreover, the authors use multiple methods and look at data from multiple unrelated languages to assess the generalizability of their conclusions, and are pretty clear about the limitations of their current findings. As a computational cognitive modeler focusing on language acquisition, I especially appreciate the difficulties that go into (i) designing a computational model that connects concretely to empirical acquisition data, and (ii) interpreting model results in a cognitively-meaningful way. Because of this, I found myself deeply interested in what I would consider relevant modeling details, and unfortunately a bit confused by some of the main takeaways without those details. If possible, I’d love to see a revision that included some of that information, focusing on the aspects I mention in more detail below. Besides this, I had a few other specific thoughts that I discuss below.*

*(1) The model*

*(a) Model input, especially for children of different ages*

*It seems important to use input data distributions from children of the ages intended to be modeled (4;0-5;0, 5;6-6;6), as it’s possible that input frequencies (particularly for uses of individual lexical items) would shift as children get older. Relatedly, children’s perception of the relevant semantic properties may also be developing over time -- right now, adult data are used as a standin because that’s the empirical data available (which is really great to have!). But that, along with the changing input frequencies, might explain why the model doesn’t capture younger (immature) child behavior.*

**Author Response: **We have now included an “Adult- versus Child-directed speech” manipulation (see subheading of this name) for English, Hebrew and Japanese (no suitable corpora were available for Hindi and K’iche’). We would certainly agree that children’s semantic knowledge is unlikely to be adultlike, so in the absence of semantic rating data from children we now include (“Study 3: Further computational modeling”) no-semantics models in which this information is removed, to investigate whether it is possible to learn these restrictions in a purely lexical way. Other manipulations in this new modeling section are also indicative here, including “dropout” which simulates – amongst other things – missing or incomplete semantic information.

*More generally, to capture children’s knowledge at different ages with the kind of incremental model you have, I think you’d want to start the model off with some base level of knowledge (the equivalent of an informed Bayesian prior) that corresponds to what a child of that age is meant to know. For instance, if you wanted to pursue this idea, I could imagine running the model for the 4;0-5;0 child data, and then using that end state of that model as the start state for running the 5;6-6;6 child data (assuming the input frequency data actually differed between these two groups).*

*I don’t think the current manuscript needs to do this, but I think it’s worth discussing as a potential limitation and/or future work.*

**Author Response:** We have added child-directed counts where we can but, as we note, these corpora are very small and so “were relatively impoverished in terms of the relevant forms”; And even this is collapsing across ALL available speech to children in the relevant language, regardless of age. So we have adopted R3’s suggestion of noting that “The lack of suitable child-directed corpora – and, in particular, corpora that are sufficiently dense as to capture any age-by-age variation in the relevant caregiver speech – is a limitation that should ideally be addressed in future research”

*(b) Model implementation*

*(i) The manuscript notes that the model used is the very same oneimplemented by Ambridge et al. 2020, but it would be helpful just to give a very basic sketch of some of the finer details when it’s first presented (e.g., how many input units are there, where the input data come from, etc.)*

**Author Response: **We have now added detail of this modeling (see section “Because we adopt the same model…”), and have now added a new modeling section (Study 3) in which we formally experiment with parameter settings.

*More generally, given the explanatory goals you have with the input features you’re using, I’m curious about them motivation for using a neural network, rather than a more transparent classifier (like SVM, logistic regression) or cognitive model (like Bayesian inference). It seems like a more-transparent modeling methodology would speak to the goal of how predictive/explanatory the hypothesized features are. Of course, I realize you’ve already developed the model in the Ambridge et al. 2020 previous work, and want to test it here. But I do wonder if the explanatory goal would have been better-served by a different modeling choice. Perhaps the model incorporates information in a distributed way (e.g., like current word embedding approaches like GloVe or RoBERTa do), and these input representations would be hard to replicate in a different modeling type? At any rate, I do think a little more background on the modeling choice might be helpful somewhere in this paper for readers like me.*

**Author Response: **The additional information about the modeling should hopefully make this clear. In particular, the reason we chose this methodology is because we believe that it best maps onto the task facing learners (although of course, no model is a perfect match). In production, learners have a message to convey (including causation or not) which includes the relevant caused event/verb (and the detailed semantic features of it), and must choose a construction to convey it. Of course, this is a vast oversimplification. But alternative tasks like Bayesian clustering, in our view, map less well onto this real-world learning scenario. Our main goal is not so much to pick apart different hypothesized features (e.g., semantics, entrenchment, preemption) but – almost the opposite – to show how these fall naturally out of a psychologically-plausible learning mechanism. That said, we do now pick apart the effects of semantics and lexical learning by running models in which the semantic information is removed. These demonstrate that the semantics is crucial to learning, and particularly in generalizing to novel verbs (see next comment).

*(ii) Split-half validation: I think it’s good to see the split-half validation, even if you couldn’t do it for K’iche’ in Study 2. The results from split-half validation are much more believable, as opposed to the test-on-training when you use the full 60. That is, the split-validation captures what the model has learned more generally rather than what the model has learned (and potentially overfit) for these data. I know the full-60 correlations have higher values, but the split-half validation is more credible for the interesting explanatory claims you want to make about input frequency and the semantic features. Because of this, I’d be careful about playing up the full-60 modeling results compared with the split-half validation*.

**Author Response: **This does seem to reflect – as per the next comment – a miscommunication on our part about how the split-half test works…

 ( *iii) The discussion currently says about the modeling approach: “Given that an identical model can predict...without having been trained on any of these datasets”. I think I may have misunderstood something fundamental about the model then (and this may make my previous comments about the full-60 vs. split-half validation make less sense). I thought the model, as a neural net, gets trained to predict the correct output value on the basis of the input, and is given pairs of input-output to learn from over time. So, because the model has seen all 60 verbs (for the full-60 models) or 30 (for the split-half validation models), the model has in fact been trained (for the full-60) or partially trained (for the split-half) on these datasets*. 

**Author Response: **As we now make explicit “note that this “split half validation” did NOT consist of training the model on half of the participants’ grammaticality judgments and having it predict the held-out half. Rather, it consisted of withholding half of the verbs from the corpus-derived training set, before interrogating it for its predictions for the held-out verbs”. We also give a similar reminder whenever we discuss the split-half test.

*If this isn’t right, then I think the manuscript would definitely benefit from more description of the model itself and how it was trained, since this seems like a really important aspect of what you want to say in the general discussion. To me, the key idea is that certain aspects of the input have great explanatory power (with r around 0.5): the input frequencies of the forms, and the four semantic features. With these viewed as the relevant part of the input, a modeled learner can both overgeneralize and retreat from overgeneralization.*

**Author Response: **Yes, we have now added a great deal more information about the model’s training; both for the original model (see above) and the new expanded modeling work(Study 3).

*(c) What the model is meant to do: Related to the previous point, I think it may be helpful to draw out the explanatory power more of the frequency and semantic factors you investigate. If I’m understanding the goal of the modeling correctly, that’s really what the model is meant to do: predict when overgeneralization does and doesn’t happen, on the basis of these factors.*

**Author Response:** Yes, this is now made explicit in the section “Verb semantics are crucial. “

*(2) What counts as overgeneralization*

*(i) When I was looking at Figure 3, I had a minor point of confusion about what counts as an overgeneralization. Overgeneralizations are defined as items where kids use the forms more equally than adults (i.e., kids think the forms are less different than adults). But, why not focus on the more natural overgeneralizations for the causative, where adults prefer the more-transparent (“A made B VERB”) over the less-transparent (“A VERBed B”) form moreso than children do? In Hindi for example, it seems like overgeneralizations also include items where adults prefer the less-transparent (“A VERBed B”) over the more-transparent, because the adult difference score is positive.*

**Author Response:** Sorry, this was due to confusing colour choices in Figure 3, which originally went from red (+2) to green (-2). We agree with R3 that Overgeneralizations occur in both directions, so now +2 and -2 are both red, grading into green at the midpoint (zero). So (perfect) green indicates that children’s preference for the more-over less transparent form (or vice versa) is the same as adults (i.e., zero degree of overgeneralization). Having red for maximal ovegeneralizations in both directions sounds confusing at first, but it is more intuitive when combined with the position of the verbs on the plot: Red items on the left of the plot mean that children’s preference for the less over more transparent form is smaller than it should be (e.g., English children fail to sufficiently dislike Someone danced the boy as compared to Someone made the boy dance). Red items on the right of the plot means that children’s preference for the more over less transparent form is smaller than it should be (e.g., English children fail to sufficiently dislike Someone made the room decorate as compared to Someone decorated the room). We also changed the colour scheme for Figures 1-2 to avoid suggesting incorrectly that Figures 1-2 and Figure 3 are comparable with regard to colour coding.

*(ii) Study 2:*

*The current manuscript suggests that less-transparent forms (“A VERBed B”) are more frequent in children’s input, and that’s why children use the less-transparent forms more than adults. Is the input difference in general for causative verbs, or just for individual verbs? That is, is the less-transparent more common for causative verbs as a whole, and that’s what you think is causing the overgeneralization here? If so, then it means children have grouped together “causative verbs” as a class, and are tracking frequencies about that class.  If instead you mean that less-transparent forms are more common for individual verbs, then do you mean that “overgeneralization” (as defined here by using the less-transparent form more often than adults) is simply driven by the input? That is, it’s just a reflection of an input that supports overgeneralizations, when defined as using the less-transparent form more often than you should. (Side note: This seems a little different from allowing a form that adults categorically think is not allowed. It might be helpful to note this.)*

**Author Response:** We have removed this section because further detailed investigation of our results, including via the new modeling, suggests that although the model correlates well with both child and adult data, it does not actually simulate specific verb-by-verb patterns in the retreat from overgeneralization.

*Follow up if you meant children’s input has more less-transparent uses for individual verbs: Is this something you could test for explicitly, by just seeing how well input frequency accounts for child judgments, and not including the 4 semantic features?*

**Author Response** :Yes, we have now investigated this (again see section “Verb semantics are crucial”).

*(3) Very minor thing: Figure resolution*

*Several figures in my pdf of the manuscript were rather fuzzy -- figures 1-3, an 9-11. It would be good to get better resolution versions of these.*

**Author Response:** Yes, we have now supplied these as PDFs instead which should address the issue.

# Peer review: Reviewer response for version 1

**DOI:** 10.21956/openreseurope.14078.r26710

**Published:** 2021-05-04

**Authors:** Vsevolod Kapatsinski

**Affiliations:** 1 Department of Linguistics, University of Oregon, Eugene, OR, USA

This article reports novel data on overgeneralization, a core topic in the acquisition of language. It expands a prior study by the researchers to a younger age range. An important strength of this paper is the cross-linguistic breadth of the investigation, which is unprecedented except for the paper's companion piece (Ambridge *et al*., 2020). The main weakness of the paper is that the authors do not show that their computational model can account for the developmental trajectory. This is important because the main claim of the paper is that the model provides an account of *retreat* from overgeneralization. A second, related weakness of the analyses is that the authors say very little about how performance changes with age. Third, assuming that the model does account for the developmental trajectory, it would be important to show *why* it does. I elaborate on these issues below.

**Major points:**

**1. What properties of the computational model are important to account for the behavior?**

 The computational model used in this paper is a simple two-layer connectionist network, in which the input consists of a local (one-hot) encoding of verb identity, and 4 continuous semantic parameters thought to be relevant to the choice between the causative constructions. Based on the preceding paper by the authors (Ambridge *et al*., 2020), the model also has an input node that represents whether the input is causative. The existence of this node is likely crucial for predicting that the more frequent causative construction will be overgeneralized, until the associations of specific semantic and lexical cues strengthen enough to override this initial bias. The output layer consists of three nodes for direct causative, indirect causative and 'other'. The learning rule is not described here, but is said to be a variant of Widrow-Hoff in Ambridge *et al*. (2020). It seems likely that the discriminative nature of this learning rule is crucial for the performance of the model, but this is not shown or discussed. It is also possible that a simple Hebbian learning rule would also do.

 It is important to provide a full description of the model here so that the work could be replicated, and the paper could be read as a stand-alone piece. A full description should include: the learning rule, the activation function on the output node, learning rate, and any other parameters that were set. The authors also need to describe whether they have attempted to use different learning rules, activation functions, or parameter settings. Such explorations would be very informative for determining what properties of the model are responsible for its ability to explain the human behavior. In particular, does it matter that the learning rule is discriminative?

**2. How does the behavior change with age?**

 It is not clear how the behavior in question changes with age. There are several possibilities, none of which are mutually exclusive. First, it could be that the children are more accepting of deviation from prior experience than adults (e.g., Kapatsinski *et al*., 2017) ^1^. It could also be that children are gradually picking up on the semantic predictors conditioning the choice of the construction (Goldberg, 2019). Finally, it is also possible that, with age, children become more confident in their estimates of how individual verbs behave (e.g., suggested by Erker & Guy, 2012) ^2^. Without knowing what changes with age, we cannot tell what the model should explain. I would like to see interactions between age and verb, and between age and the semantic predictors. According to p.20, "the main difference between 4;0-5;0 and 5;6-6;6 year olds is simply an across the board decrease in the production of overgeneralization errors, rather than any change in their by-verb patterning." I would like to see a statistical evaluation of this claim. 

**3. Does the model predict how the behavior changes with age?**

 Differences in the model's construction activations across verbs are shown to correlate with differences in ratings, judgments and production probabilities of both children and adults (in at least some languages). However, it is not clear how much of what the model is capturing here is variance shared between children and adults. That is, the model might be capturing semantic effects on construction choice that are equally robust in adults and children. If the model can account for retreat from overgeneralization, it is important to show that the model predicts how the behavior *changes* across age. The fact that the model does not show a better fit to kid data early in training and a better fit to adult data late in training is problematic if the correlations reliably change across development. If they don't, then the authors should show that the model captures what *does* change, even if this is only a simple increase in the use of the rarer construction with age. 

**4. How important are semantics, verbs, and the causative node?**

 Assuming the model can account for the changes in construction use with age, I would like to see what is responsible for those changes in the model. In particular, the model could be lesioned by removing the verb nodes, semantic nodes and/or the causative node, or injecting noise into the representations or the connections involving these different inputs. From the statement on p.5 that there is only a small decrement in performance when the model is tested on novel verbs, it appears that most of the model's performance comes from capturing the semantic influences on construction choice. Would removing the verbs altogether reduce the model's performance? The causative node seems necessary to predict overgeneralization of the frequent construction early on, though a bias node might also work for that purpose.

**Minor points:**

**5.** **Is there an interaction between directness and social desirability?**

 It seems to me that the social desirability effect would not disappear from calculating difference scores (p.7). The two constructions (at least in English) differ in directness of causation, so it seems plausible that socially undesirable actions would favor the periphrastic construction (as in "caused to die" vs. "killed"). Does social desirability not correlate with constructional choice? Would it make sense to include it in the model as another cue?

**6. Are the same semantic dimensions relevant to all languages?**

 There seems to be an assumption that the same four semantic dimensions should be relevant to the choice of the construction in all languages. I am curious as to whether this assumption is on a solid footing. It does not seem particularly surprising to me that there would be languages in which the choice of the causative construction is based on some variables other than the ones mentioned. Could this be the case in K'iche Mayan?

**7. More description of the training corpora**

 More details on the training corpora are needed to evaluate whether they are representative of the input to children.

If applicable, is the statistical analysis and its interpretation appropriate?

Partly

Is the study design appropriate and is the work technically sound?

Partly

Is the work clearly and accurately presented and does it engage with the current literature?

Yes

Are the conclusions drawn adequately supported by the results?

Partly

Are sufficient details of methods and analysis provided to allow replication by others?

Partly

Are all the source data and materials underlying the results available?

Yes

Reviewer Expertise:

My research deals with the role of domain-general learning mechanisms in language acquisition.

I confirm that I have read this submission and believe that I have an appropriate level of expertise to confirm that it is of an acceptable scientific standard, however I have significant reservations, as outlined above.

## References

[1] : Perceptual Learning of Intonation Contour Categories in Adults and 9- to 11-Year-Old Children: Adults Are More Narrow-Minded. *Cogn Sci* .41(2) : 10.1111/cogs.12345 383-415 10.1111/cogs.12345 26901251PMC4993691

[2] : The role of lexical frequency in syntactic variability: Variable subject personal pronoun expression in Spanish. *Language* .2012;88(3) : 10.1353/lan.2012.0050 526-557 10.1353/lan.2012.0050

# Response

**Published:** 2022-01-07

**Authors:** Ben Ambridge

**Affiliations:** University of Liverpool, UK

We thank this reviewer for their very helpful comments. In particular, the prompt to investigate the extent to which the model can explain the *retreat* from overgeneralization developmentally inspired new modeling work that caused us to rethink exactly what the model is telling us. Reviewer comments in italics.

*This article reports novel data on overgeneralization, a core topic in the acquisition of language. It expands a prior study by the researchers to a younger age range. An important strength of this paper is the cross-linguistic breadth of the investigation, which is unprecedented except for the paper's companion piece (Ambridge et al., 2020). The main weakness of the paper is that the authors do not show that their computational model can account for the developmental trajectory. This is important because the main claim of the paper is that the model provides an account of retreat from overgeneralization. A second, related weakness of the analyses is that the authors say very little about how performance changes with age. Third, assuming that the model does account for the developmental trajectory, it would be important to show why it does. I elaborate on these issues below. Major points: 1. What properties of the computational model are important to account for the behavior? The computational model used in this paper is a simple two-layer connectionist network, in which the input consists of a local (one-hot) encoding of verb identity, and 4 continuous semantic parameters thought to be relevant to the choice between the causative constructions. Based on the preceding paper by the authors (Ambridge et al., 2020), the model also has an input node that represents whether the input is causative. The existence of this node is likely crucial for predicting that the more frequent causative construction will be overgeneralized, until the associations of specific semantic and lexical cues strengthen enough to override this initial bias. The output layer consists of three nodes for direct causative, indirect causative and 'other'. The learning rule is not described here, but is said to be a variant of Widrow-Hoff in Ambridge et al. (2020). It seems likely that the discriminative nature of this learning rule is crucial for the performance of the model, but this is not shown or discussed. It is also possible that a simple Hebbian learning rule would also do. It is important to provide a full description of the model here so that the work could be replicated, and the paper could be read as a stand-alone piece. A full description should include: the learning rule, the activation function on the output node, learning rate, and any other parameters that were set. The authors also need to describe whether they have attempted to use different learning rules, activation functions, or parameter settings. Such explorations would be very informative for determining what properties of the model are responsible for its ability to explain the human behavior. In particular, does it matter that the learning rule is discriminative?*

**Author Response: **We have now added detail of this modeling (see section “Because we adopt the same model…”), which in fact involves clearing up some errors in the description of the old model (in fact, the learning rule is BFGS). We did not formally experiment with parameter settings in this model, but have now added a new modeling section (Study 3) in which we did this extensively.

*2. How does the behavior change with age? It is not clear how the behavior in question changes with age. There are several possibilities, none of which are mutually exclusive. First, it could be that the children are more accepting of deviation from prior experience than adults (e.g., Kapatsinski et al., 2017)1. It could also be that children are gradually picking up on the semantic predictors conditioning the choice of the construction (Goldberg, 2019). Finally, it is also possible that, with age, children become more confident in their estimates of how individual verbs behave (e.g., suggested by Erker & Guy, 2012) Without knowing what changes with age, we cannot tell what the model should explain. I would like to see interactions between age and verb, and between age and the semantic predictors. According to p.20, "the main difference between 4;0-5;0 and 5;6-6;6 year olds is simply an across the board decrease in the production of overgeneralization errors, rather than any change in their by-verb patterning." I would like to see a statistical evaluation of this claim.*

**Author Response: **This is a very important point. In the new version, we find that, actually, not much changes with age: The relevant interactions, which we have now added (see Table 6) show that only for English and Hebrew are older children’s production data more adultlike than younger children’s. This links to the next point…

*3. Does the model predict how the behavior changes with age? Differences in the model's construction activations across verbs are shown to correlate with differences in ratings, judgments and production probabilities of both children and adults (in at least some languages). However, it is not clear how much of what the model is capturing here is variance shared between children and adults. That is, the model might be capturing semantic effects on construction choice that are equally robust in adults and children. If the model can account for retreat from overgeneralization, it is important to show that the model predicts how the behavior changes across age. The fact that the model does not show a better fit to kid data early in training and a better fit to adult data late in training is problematic if the correlations reliably change across development. If they don't, then the authors should show that the model captures what does change, even if this is only a simple increase in the use of the rarer construction with age.* 

**Author Response: **Yes, the reviewer’s more pessimistic assessment is correct here. The model indeed seems to be capturing semantic (and lexical) effects on construction choice that are robust in adults and children. It does NOT seem to be able to account for development, even when it is extensively modified in ways that might allow it to do so (Study 3). Thus the modeling work suggests that although it provides a good account of how leaners ultimately acquire verbs’ restrictions, the model does not simulate the retreat from overgeneralization itself, which likely requires an explanation from outside the grammar. We discuss this extensively in the new version, but especially in the General Discussion.

*4. How important are semantics, verbs, and the causative node? Assuming the model can account for the changes in construction use with age, I would like to see what is responsible for those changes in the model. In particular, the model could be lesioned by removing the verb nodes, semantic nodes and/or the causative node, or injecting noise into the representations or the connections involving these different inputs. From the statement on p.5 that there is only a small decrement in performance when the model is tested on novel verbs, it appears that most of the model's performance comes from capturing the semantic influences on construction choice. Would removing the verbs altogether reduce the model's performance? The causative node seems necessary to predict overgeneralization of the frequent construction early on, though a bias node might also work for that purpose.*

**Author Response: **In the new modeling section (Study 3), we ran over 2,000 different models including randomizing the semantics nodes and impairing the model’s performance in various “childlike” ways. We did not, however, specifically try removing (a) the lexical verb nodes or (b) the causative node because this would correspond to scenarios in which the child (a) does not know the lexical form of the verb (which cannot be realistic, if the child is saying the verb) or (b) does not intend a causative message (which cannot be realistic, as the aim is to investigate what children say when they do have a causative message in mind). Importantly, NONE of our modifications allowed the model to predict the child data better than the adult data, which is why we conclude that the retreat from overgeneralization likely requires additional explanation from outside the grammar.

*Minor points: 5. Is there an interaction between directness and social desirability? It seems to me that the social desirability effect would not disappear from calculating difference scores (p.7). The two constructions (at least in English) differ in directness of causation, so it seems plausible that socially undesirable actions would favor the periphrastic construction (as in "caused to die" vs. "killed"). Does social desirability not correlate with constructional choice? Would it make sense to include it in the model as another cue?*

**Author Response:** In the new version we included valence ratings and R2 is exactly right here: “for Japanese and K’iche’… children are heavily influenced by valance (also significant for Hindi): The less acceptable the action, the more children prefer the more transparent form; (e.g.,  making something break, which hints at unintentionality, is more acceptable than breaking something, which suggests a more intentional act)”.

*6. Are the same semantic dimensions relevant to all languages? There seems to be an assumption that the same four semantic dimensions should be relevant to the choice of the construction in all languages. I am curious as to whether this assumption is on a solid footing. It does not seem particularly surprising to me that there would be languages in which the choice of the causative construction is based on some variables other than the ones mentioned. Could this be the case in K'iche Mayan?*

**Author Response: **This could well be the case, but we did not investigate any semantic dimensions other than those discussed here, it would not be particularly informative for us to speculate.

*7. More description of the training corpora More details on the training corpora are needed to evaluate whether they are representative of the input to children.*

**Author Response:**  The corpus data used in the previous version were not representative of speech to children, but we have now included an “Adult- versus Child-directed speech” manipulation (see subheading of this name) for English, Hebrew and Japanese (no suitable corpora were available for Hindi and K’iche’). References

# Peer review: Reviewer response for version 1

**DOI:** 10.21956/openreseurope.14078.r26712

**Published:** 2021-04-29

**Authors:** Yi Ting Huang, Zoe Ovans

**Affiliations:** 1 Department of Hearing and Speech Sciences, University of Maryland, College Park, College Park, MD, USA; 2 Neuroscience and Cognitive Science Program, University of Maryland, College Park, College Park, MD, USA

Summary

 In this work, the authors expand on a model presented in Ambridge *et al*., 2020, which predicts causative alternation acceptability on a verb-by-verb basis. This model takes as its input both corpus data as well as adults’ semantic feature ratings for the verbs in question. In the present paper, the model is expanded to account for young children’s overgeneralization errors (e.g. “I’m dancing it” to mean “I’m causing it to dance”), both in offline comprehension and production. Notably, the authors attempt to account for children’s overgeneralizations across five languages, and find that the Ambridge *et al*., 2020 model correlates with children’s performance in most cases. These findings, they conclude, provide evidence that the discriminative learning model in question is a plausible explanation for how children retreat from verb argument structure overgeneralization errors, on mechanistic level.

 Major Comments

 To begin with, the 23 authors of this study should undoubtedly be commended for the large-scale collaborative effort this study represents! It’s also exciting to see computational research that does not shy away from fine-grained cross-linguistic comparison. The question the authors target in this work, how do children retreat from overgeneralization errors, is timely and relevant to the field, and overall their methodology seems sound. That said, there are 4 major areas of concern the authors should address in order to both make their precise claims more intelligible in the current work and to make their conclusions more convincing:

 1. Disconnect between the data presented and the research questions.

 a) The authors state in their abstract that “the present study demonstrates that a simple discriminative learning model … constitutes a plausible mechanistic account of the retreat from overgeneralization.” (p2) and later that “the problem of how language learners come to appropriately constrain their argument structure generalization looks close to being solved” (p22) because the model results correlate with both children and adults’ causative judgment data and children’s causative productions. However, it’s not explicitly stated how the present model instantiates the mechanisms for learning argument structure. Looking at the 2020 paper, it seems that the answer is approximately that children are using a combination of lexical-semantic features, a causal/non-causal binary operator, and the ability to identify the particular lexical items to intuit which causative verbs ought to alternate which way. And this conclusion is reached because the model in question approximates human performance using these particular pieces of information as input. For this paper to function as a stand-alone work, these connections ought to be spelled out here as well.

 b) If the model presented is trained on the exact same data as the model in Ambridge *et al*., 2020, what changed between the two papers is just the values of the dependent measure the model is expected to approximate. If the model successfully predicts adult judgments, it may only explain child judgments to the extent that they mirror adult judgments. And these would be all the cases where children are not making overgeneralization errors. Therefore, to what extent does the model provide a mechanistic explanation for the retreat from overgeneralization? Perhaps a more direct measure of how well the model captures overgeneralization itself would be to correlate model performance, for each verb, with verb-level estimates of children's overgeneralizations (baselined to adults judgments). 

 c) Furthermore, the 2020 paper did test the model on data from children, just slightly older ones (5-6 year-olds and 9-10 year-olds). The contribution of the current work is that the children tested were a bit younger still, at 4;0-5;0. The question this raises is: What are the data from these slightly younger children adding to our inferences about underlying generalizations? The authors state that “the majority of [children’s] overgeneralization errors are produced before [they’re 5 years old]” (page 5). It would be helpful to more clearly spell out how testing children in the year before that allows us to make new inferences beyond those made in the 2020 paper.

 2. Concerns about children’s acceptability judgments and judgment data

 a) The authors note that “the researchers who worked with the children reported that… children rat[ed] sentences, to some extent, on the basis of the social desirability of the events described” (p7). This, they reason, may have accounted for some undergeneralization errors such as children’s low ratings for “someone broke the truck.” This raises questions about the validity of the grammaticality judgments and priming task in general. To what extent does the binary decision assessment conflate social desirability and grammatical acceptability? While the words in Figures 1-3 are a bit difficult to read, it seems possible that the more overgeneralized verbs correspond to more positively-valanced ones (e.g. dance, sing, play). It seems that the authors take the correlations with adult data to be evidence that children were attending to the grammatical acceptability on the whole, but it would be more convincing to show, say, a lack of correlation between child judgments and a valance measure.

 b) The authors convincingly demonstrate that children’s acceptability judgments correlate with those of adults. They take this to mean that “at least for English-, Hebrew-, Hindi- and K’iche’-speaking, children were indeed giving meaningful judgments” (p7). However, it should be noted that if children’s responses are highly similar to adults’, and the model used here has already been shown to reliably predict adults’ judgement data, the mere correlation of model results and child judgment data should not be taken as a necessarily new finding, though this correlation appears to be the main result for Experiment 1. Simply put – if the model predictions correlate with adult data, and adult response data correlates with child response data, why wouldn’t we expect the model predictions to correlate with the child data? Indeed, for the Japanese data where the child and adult responses do not correlate, the model does not appear to capture the child judgments. 

 Additionally, the semantic feature judgments that serve as input to the model appear to be judgments from adults. It seems reasonable to assume that children’s judgments will not match adults’ – even in this paper, there are instances where authors voice concerns about children not interpreting the valence of the sentences in an adult-like way (as just discussed). If the model is to be taken as a mechanistic explanation for how children retreat from their errors, it seems necessary to have its input parallel children’s mental state as much as possible. The worry is that since the model relies on adult judgments, it might be ascribing to children knowledge that they do not yet posses. The model may therefore approximate children’s binary judgment data for the wrong reason (it’s relying on semantic information adults are privy to but children aren’t). To avoid these concerns, the authors could provide some evidence for why they expect that children’s and adults’ semantic feature judgments would either match, or differ in a non-meaningful way. 

 3. Concerns about how to interpret the correlations. 

 The verb-level correlations from child and adult performance ignore the subject-level variability of estimates. If the data were analyzed instead with mixed-effects models (where verb is a random-effects variable), would adult ratings predict children's (and vice versa)? 

 4. Concerns about the model data being run using adult corpus data

 a) For both studies, it appears that the authors ran the Ambridge *et al*., 2020 model on causative alternation data gleaned from adult speech, and not speech to children. Given that the model is concluded to provide a potential mechanistic explanation for children’s retreat from overgeneralization, it seems right to include causative alternation frequencies present in the speech that children themselves hear. Or at least, some evidence that the frequencies don’t differ in the two types of speech is needed. 

 b) Finally, it’s not clear from the present work that the semantic input nodes were necessary aspects of the model. Is it possible that the corpus frequencies just mapped on well to adults’ and children’s judgments? The opposite could also be asked – was the corpus frequency data needed in addition to the semantic features? 

 Minor Comments

 1. Where possible, it’d be helpful to have vectorized images for the figures, or at least larger files. As is, it is difficult to read the individual words in Figures 1-3 and 9-10. Figure 11, while legible, is also a bit blurry. Once the figures are reproduced in higher definition it may additionally be useful to display the words in such a way that they don’t overlap on Figures 1-3 & 9-10 (perhaps with a slight jitter).

 2. Page 3-4 – A list of citations is given for papers and books that have investigated overgeneralization errors in English. While it’s helpful to have a collection of citations for the past work on these errors, there is no explicit characterization of what this work has concluded. In order to show that it’s relevant to the specific question of how learners overcome these errors, some curation of these citations is necessary. 

 3. Page 4 – A trio of theories are introduced here “preemption, conservatism via entrenchment (both statistical-learning theories) and verb semantics.” It seems to be a main goal of this work to show that these theories (or at least the unified version) provide the correct account of children’s retreat from overgeneralization errors, so it would be helpful to give a precise definition for them here, as well as provide citations for their use. While this section is summarizing Ambridge *et al*., 2020, it becomes difficult to evaluate the main conclusions in the present paper on its own without (re)articulating these theories and how the model instantiates a combination of the three.

 4. Page 11 – The authors make reference to the notion that “social desirability may be particularly salient in the more collectivist Japanese culture” in their explanation for the lack of correlation between the child and adult judgments for the Japanese data. While they provide a citation to this effect, this explanation appears to be a bit vague. In order to avoid being reductive in including cultural collectivism as a possible explanation, it’d be necessary to first establish that the cultures of other children in this study are less collectivist (and there is at least some evidence that this is a false assumption, e.g. Oyserman, Coon, & Kemmelmeier, 2002). Barring evidence to this effect, the cultural explanation doesn’t convincingly explain the data pattern.

 5. Page 20 – The authors state: “Comparison of Figure 9 (4;0-5;0) and Figure 10 (5;6-6;6) indicates that, by this later age, overgeneralization errors have all but ceased for English, Hindi and Japanese, and decreased considerably for Hebrew.” However, this difference is not particularly obvious from cross-figure comparison. A single figure comparing the results from both age groups on one graph (or at least closer together on a page) for each language would highlight this better. 

 Typesetting

 For Figures 4-8 & 11, the labels could be cleaned up a bit (e.g. spaces added instead of underscores).

If applicable, is the statistical analysis and its interpretation appropriate?

Partly

Is the study design appropriate and is the work technically sound?

Yes

Is the work clearly and accurately presented and does it engage with the current literature?

Yes

Are the conclusions drawn adequately supported by the results?

Partly

Are sufficient details of methods and analysis provided to allow replication by others?

Partly

Are all the source data and materials underlying the results available?

Yes

Reviewer Expertise:

My co-reviewer and I have expertise in language development and psycholinguistics, specifically in the area of argument structure.

We confirm that we have read this submission and believe that we have an appropriate level of expertise to confirm that it is of an acceptable scientific standard, however we have significant reservations, as outlined above.

# Response

**Published:** 2022-01-07

**Authors:** Ben Ambridge

**Affiliations:** University of Liverpool, UK

We thank these reviewers for their very helpful comments. In particular, the prompt to investigate the extent to which the model can explain the *retreat* from overgeneralization developmentally inspired new modeling work that caused us to rethink exactly what the model is telling us. Reviewer comments in italics.

*Summary: In this work, the authors expand on a model presented in Ambridge et al., 2020, which predicts causative alternation acceptability on a verb-by-verb basis. This model takes as its input both corpus data as well as adults’ semantic feature ratings for the verbs in question. In the present paper, the model is expanded to account for young children’s overgeneralization errors (e.g. “I’m dancing it” to mean “I’m causing it to dance”), both in offline comprehension and production. Notably, the authors attempt to account for children’s overgeneralizations across five languages, and find that the Ambridge et al., 2020 model correlates with children’s performance in most cases. These findings, they conclude, provide evidence that the discriminative learning model in question is a plausible explanation for how children retreat from verb argument structure overgeneralization errors, on mechanistic level. Major Comments To begin with, the 23 authors of this study should undoubtedly be commended for the large-scale collaborative effort this study represents! It’s also exciting to see computational research that does not shy away from fine-grained cross-linguistic comparison. The question the authors target in this work, how do children retreat from overgeneralization errors, is timely and relevant to the field, and overall their methodology seems sound. That said, there are 4 major areas of concern the authors should address in order to both make their precise claims more intelligible in the current work and to make their conclusions more convincing: 1. Disconnect between the data presented and the research questions. a) The authors state in their abstract that “the present study demonstrates that a simple discriminative learning model … constitutes a plausible mechanistic account of the retreat from overgeneralization.” (p2) and later that “the problem of how language learners come to appropriately constrain their argument structure generalization looks close to being solved” (p22) because the model results correlate with both children and adults’ causative judgment data and children’s causative productions. However, it’s not explicitly stated how the present model instantiates the mechanisms for learning argument structure. Looking at the 2020 paper, it seems that the answer is approximately that children are using a combination of lexical-semantic features, a causal/non-causal binary operator, and the ability to identify the particular lexical items to intuit which causative verbs ought to alternate which way. And this conclusion is reached because the model in question approximates human performance using these particular pieces of information as input. For this paper to function as a stand-alone work, these connections ought to be spelled out here as well.*

**Author Response:** We have added detail about how the model worked, and how it instantiates mechanisms for learning argument structure; See sections beginning “Rather, the model was trained to “predict” the forms…” and “Because we adopt the same model in the present article, it is important to fully set out here the details of its architecture.”

*b) If the model presented is trained on the exact same data as the model in Ambridge et al., 2020, what changed between the two papers is just the values of the dependent measure the model is expected to approximate. If the model successfully predicts adult judgments, it may only explain child judgments to the extent that they mirror adult judgments. And these would be all the cases where children are not making overgeneralization errors. Therefore, to what extent does the model provide a mechanistic explanation for the retreat from overgeneralization? Perhaps a more direct measure of how well the model captures overgeneralization itself would be to correlate model performance, for each verb, with verb-level estimates of children's overgeneralizations (baselined to adults judgments).*

**Author Response:** In the previous version of this paper, and in Ambridge *et al* (2020), we rather overstated the success off the model by conflating two questions (1) Does the model eventually come to mirror adults’ judgments and (2) Does it, along the way, show a similar by-verb overgeneralization-than-retreat pattern to children. In fact, although the answer to the first question is yes, the answer to the second question is no. We discuss this throughout the new version, but most explicitly in the General Discussion (section beginning “At one level…”)

*c) Furthermore, the 2020 paper did test the model on data from children, just slightly older ones (5-6 year-olds and 9-10 year-olds). The contribution of the current work is that the children tested were a bit younger still, at 4;0-5;0. The question this raises is: What are the data from these slightly younger children adding to our inferences about underlying generalizations? The authors state that “the majority of [children’s] overgeneralization errors are produced before [they’re 5 years old]” (page 5). It would be helpful to more clearly spell out how testing children in the year before that allows us to make new inferences beyond those made in the 2020 paper.*

**Author Response:** The most important contribution in the present article is not so much the binary grammaticality judgments – which, we acknowledge do not really do much more than bring the age down a bit – but the production data. These data are important to address the potential concern that acceptability judgments are a meta-linguistic task that do not provide a reliable measure of children’s knowledge and/or performance. It is therefore important to show, as the new version does, that the model (and the new models) predict production data as well as just judgment data.

*2. Concerns about children’s acceptability judgments and judgment data a) The authors note that “the researchers who worked with the children reported that… children rat[ed] sentences, to some extent, on the basis of the social desirability of the events described” (p7). This, they reason, may have accounted for some undergeneralization errors such as children’s low ratings for “someone broke the truck.” This raises questions about the validity of the grammaticality judgments and priming task in general. To what extent does the binary decision assessment conflate social desirability and grammatical acceptability? While the words in Figures 1-3 are a bit difficult to read, it seems possible that the more overgeneralized verbs correspond to more positively-valanced ones (e.g. dance, sing, play). It seems that the authors take the correlations with adult data to be evidence that children were attending to the grammatical acceptability on the whole, but it would be more convincing to show, say, a lack of correlation between child judgments and a valance measure.*

**Author Response:** We have added analyses showing that, in many cases there IS indeed a correlation with a valence measure (see “Verb valance ratings (from Warriner…)”. This is particularly the case for Japanese and K’iche’ binary judgment data, which could well be a reason why children’s binary judgments do not correlate well with adults’ for these languages). However, for English, Hebrew and Hindi the significant correlations for binary judgment data reported in the previous version (now “upgraded” to mixed-effects models) survive the introduction of valence ratings as a control predictor. No valance effects are observed for the production data; it seems they are a consequence of asking children to rate forms for “acceptability” – and failing to fully ensure that they understand this to mean purely “grammatical” acceptability.

*b) The authors convincingly demonstrate that children’s acceptability judgments correlate with those of adults. They take this to mean that “at least for English-, Hebrew-, Hindi- and K’iche’-speaking, children were indeed giving meaningful judgments” (p7). However, it should be noted that if children’s responses are highly similar to adults’, and the model used here has already been shown to reliably predict adults’ judgement data, the mere correlation of model results and child judgment data should not be taken as a necessarily new finding, though this correlation appears to be the main result for Experiment 1. Simply put – if the model predictions correlate with adult data, and adult response data correlates with child response data, why wouldn’t we expect the model predictions to correlate with the child data? Indeed, for the Japanese data where the child and adult responses do not correlate, the model does not appear to capture the child judgments.*

**Author Response:** Certainly, given that (a) the model predicts adult data and (b) children’s data are highly correlated with adult data, we would certainly expect to see that the model predicts children’s data. The more interesting question is not simply DOES the model predict children’s data, but the EXTENT to which it predicts children’s data. The fact that the model (including in all its additional versions; new for this revised version) predicts children’s data LESS well than adults is important and interesting, as it suggests that – as we note throughout this new paper – the solution to the retreat from overgeneralization seems to live outside the grammar.

*Additionally, the semantic feature judgments that serve as input to the model appear to be judgments from adults. It seems reasonable to assume that children’s judgments will not match adults’ – even in this paper, there are instances where authors voice concerns about children not interpreting the valence of the sentences in an adult-like way (as just discussed). If the model is to be taken as a mechanistic explanation for how children retreat from their errors, it seems necessary to have its input parallel children’s mental state as much as possible. The worry is that since the model relies on adult judgments, it might be ascribing to children knowledge that they do not yet possess. The model may therefore approximate children’s binary judgment data for the wrong reason (it’s relying on semantic information adults are privy to but children aren’t). To avoid these concerns, the authors could provide some evidence for why they expect that children’s and adults’ semantic feature judgments would either match, or differ in a non-meaningful way.* 

**Author Response:** We would certainly agree that children’s semantic knowledge is unlikely to be adultlike, but we are not aware of any directly relevant evidence on this issue. So, rather than simply speculate, we have introduced a new modeling section (“Study 3: Further computational modeling”) in which we attempt to “instantiate various limitations that correspond to those facing children”. Most relevantly, we include no-semantics models in which this information is removed, to investigate whether it is possible to learn these restrictions in a purely lexical way. Other manipulations are also indicative here, including “dropout” which simulates – amongst other things – missing or incomplete semantic information.

*3. Concerns about how to interpret the correlations.  The verb-level correlations from child and adult performance ignore the subject-level variability of estimates. If the data were analyzed instead with mixed-effects models (where verb is a random-effects variable), would adult ratings predict children's (and vice versa)?* 

**Author Response: **Yes, this is an important point: In the new version we now report mixed-effects models rather than simple by-verb correlations, for both the binary-judgment and production studies.

*4. Concerns about the model data being run using adult corpus data a) For both studies, it appears that the authors ran the Ambridge et al., 2020 model on causative alternation data gleaned from adult speech, and not speech to children. Given that the model is concluded to provide a potential mechanistic explanation for children’s retreat from overgeneralization, it seems right to include causative alternation frequencies present in the speech that children themselves hear. Or at least, some evidence that the frequencies don’t differ in the two types of speech is needed.*

**Author Response:** We have now introduced versions of the model based on child-directed speech (see “Adult- versus Child-directed speech”), for the languages for which suitable corpora were available (English, Hebrew and Japanese).

*b) Finally, it’s not clear from the present work that the semantic input nodes were necessary aspects of the model. Is it possible that the corpus frequencies just mapped on well to adults’ and children’s judgments? The opposite could also be asked – was the corpus frequency data needed in addition to the semantic features?*

**Author Response:** As noted above, we have now run models with neutralized semantic information, but it is not possible to remove corpus frequency information because training the model on a flat distribution would involve training it on ungrammatical utterances with the same frequency as grammatical utterances. This is not only only unrealistic, but changes the “right answer” that the model is attempting to learn. E.g., if we presented “Someone made the boy laugh” and “*Someone laughed the boy” with equal frequency, we are effectively telling the model that the two forms are equally grammatical, which would entail specifically designing it to learn a language with different properties to the natural languages included in the present study.

*Minor Comments 1. Where possible, it’d be helpful to have vectorized images for the figures, or at least larger files. As is, it is difficult to read the individual words in Figures 1-3 and 9-10. Figure 11, while legible, is also a bit blurry. Once the figures are reproduced in higher definition it may additionally be useful to display the words in such a way that they don’t overlap on Figures 1-3 & 9-10 (perhaps with a slight jitter).*

**Author Response:**  Yes, we have fixed this and now supply PDF files instead.

*2. Page 3-4 – A list of citations is given for papers and books that have investigated overgeneralization errors in English. While it’s helpful to have a collection of citations for the past work on these errors, there is no explicit characterization of what this work has concluded. In order to show that it’s relevant to the specific question of how learners overcome these errors, some curation of these citations is necessary.*

**Author Response:** We have now done this in the section beginning “Previous studies had attempted to explain this phenomenon…”

*3. Page 4 – A trio of theories are introduced here “preemption, conservatism via entrenchment (both statistical-learning theories) and verb semantics.” It seems to be a main goal of this work to show that these theories (or at least the unified version) provide the correct account of children’s retreat from overgeneralization errors, so it would be helpful to give a precise definition for them here, as well as provide citations for their use. While this section is summarizing Ambridge et al., 2020, it becomes difficult to evaluate the main conclusions in the present paper on its own without (re)articulating these theories and how the model instantiates a combination of the three.*

**Author Response:** The same section now also defines these terms.

*4. Page 11 – The authors make reference to the notion that “social desirability may be particularly salient in the more collectivist Japanese culture” in their explanation for the lack of correlation between the child and adult judgments for the Japanese data. While they provide a citation to this effect, this explanation appears to be a bit vague. In order to avoid being reductive in including cultural collectivism as a possible explanation, it’d be necessary to first establish that the cultures of other children in this study are less collectivist (and there is at least some evidence that this is a false assumption, e.g. Oyserman, Coon, & Kemmelmeier, 2002). Barring evidence to this effect, the cultural explanation doesn’t convincingly explain the data pattern.*

**Author Response: **We have removed this passage because in fact the newly-introduced valence predictor predicts children’s data (on the binary judgment task) for Hindi and K’iche’ as well as Japanese, so a cultural explanation does not seem likely here.

*5. Page 20 – The authors state: “Comparison of Figure 9 (4;0-5;0) and Figure 10 (5;6-6;6) indicates that, by this later age, overgeneralization errors have all but ceased for English, Hindi and Japanese, and decreased considerably for Hebrew.” However, this difference is not particularly obvious from cross-figure comparison. A single figure comparing the results from both age groups on one graph (or at least closer together on a page) for each language would highlight this better.* 

**Author Response: **The figures are already very busy, so we have not been able to combine them into a single plot; but we hope the typesetter will align them closely on the page to allow for such comparison to be possible.

 ​​​​​​​

 Typesetting For Figures 4-8 & 11, the labels could be cleaned up a bit (e.g. spaces added instead of underscores).

**Author Response: **Done – thanks!
